# Rapidly Adaptive All‐covalent Nanoparticle Surface Engineering

**DOI:** 10.1002/chem.202101042

**Published:** 2021-05-21

**Authors:** Marta Diez‐Castellnou, Rongtian Suo, Nicolas Marro, Saphia A. L. Matthew, Euan R. Kay

**Affiliations:** ^1^ EaStCHEM School of Chemistry University of St Andrews North Haugh St Andrews KY16 9ST UK

**Keywords:** acetals, adaptive colloidal nanoparticles, dynamic covalent chemistry, gold nanoparticles, hydrazones

## Abstract

Emerging nanotechnologies demand the manipulation of nanoscale components with the same predictability and programmability as is taken for granted in molecular synthetic methodologies. Yet installing appropriately reactive chemical functionality on nanomaterial surfaces has previously entailed compromises in terms of reactivity scope, functionalization density, or both. Here, we introduce an idealized dynamic covalent nanoparticle building block for divergent and adaptive post‐synthesis modification of colloidal nanomaterials. Acetal‐protected monolayer‐stabilized gold nanoparticles are prepared via operationally simple protocols and are stable to long‐term storage. Tunable surface densities of reactive aldehyde functionalities are revealed on‐demand, leading to a wide range of adaptive surface engineering options from one nanoscale synthon. Analytically tractable with molecular precision, interfacial reaction kinetics and dynamic surface constitutions can be probed in situ at the ensemble level. High functionalization densities combined with rapid equilibration kinetics enable environmentally adaptive surface constitutions and rapid nanoparticle property switching in response to simple chemical effectors.

## Graphical abstract

Acetal‐terminated surface ligands stabilize idealized colloidal nanoparticle building blocks. On‐demand activation of the masked aldehyde functionality occurs rapidly under mild conditions with precise stoichiometric control, revealing a versatile platform for all‐covalent post‐synthesis surface engineering. On‐surface dynamic covalent transformations lead to diverse structural and functional modifications including rapidly adaptive environment‐responsive nanomaterials.

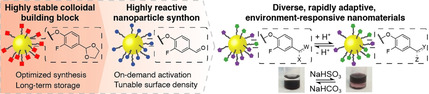

## Introduction

Nanoscale components with programmable chemical reactivity can form the basis of a comprehensive synthetic toolkit that fully integrates nanomaterials with current‐day (macro)molecular and supramolecular technologies. Such capabilities will afford enabling methods for nanoscience by facilitating the chemical transformation of nanomaterials without requiring specialist expertise or extensive methodology optimization. Ideally, a relatively small number of nanoscale building blocks displaying predictable reactivities may be divergently modified, manipulated and combined to produce a wide variety of end‐products using simple chemospecific transformations.^[1]^

Dynamic covalent exchange reactions are emerging as a powerful tool for post‐synthesis surface engineering of nanoparticles in colloidal solution.[^1c^, ^1d^] We have recently developed metal nanoparticles stabilized by hydrazone‐functionalized surface monolayers. On‐demand chemospecific transformations direct these dynamic covalent nanoparticle (DCNP) building blocks down different reaction pathways to access a variety of surface‐bound molecular structures,^[2]^ manipulate and tune nanoparticle physicochemical properties,[^2^, ^3^] and construct adaptive nanoparticle assemblies.^[2b]^ Alternative adaptive functionalization strategies have typically relied on noncovalent interactions between environmentally sensitive oligonucleotides,^[4]^ or artificial host‐guest systems,^[5]^ which are by comparison relatively weak and often non‐specific. Conversely, permanent surface modifications are often achieved via ligand exchange, but this requires optimization for each different nanoparticle core material, can introduce surface defects, reconstructions or core size changes and produces mixed‐monolayer compositions that are governed by system‐specific nonlinear relationships.^[6]^ DCNP synthons offer the opportunity to combine the structural and functional robustness of abiotic molecules with chemospecific and adaptive transformations under thermodynamic control. The stability of covalently bonded systems, however, is accompanied by relatively slow exchange kinetics, which we have found to be further retarded by surface confinement when applied for nanoparticle surface engineering.^[2b]^ We now report a “best‐of‐both‐worlds” DCNP synthon that addresses the shortcomings of both our previous technology and the alternative functionalization approaches. With this new DCNP design, rapid surface transformations are combined with a high degree of predictive control and the structural and functional stability of covalent bonds, enabling adaptive and environment‐responsive tuning of nanomaterial characteristics.

Carbonyl functional groups are attractive for their rich reversible and irreversible covalent chemistry, which has been widely exploited in bioconjugation,^[7]^ polymer ligation,^[8]^ interfacial‐functionalization strategies,^[9]^ and countless dynamic molecular networks, assemblies and machines.^[10]^ For metal nanoparticle cores, incorporating aldehyde functional groups is particularly challenging, as these are unstable under the reducing conditions required for nanoparticle synthesis, while also being sensitive to oxygen‐based radicals that can be generated by small metallic clusters.^[11]^ Consequently, aldehydes have previously been incorporated on metal nanoparticle cores using post‐synthesis ligand exchange, achieving only very low functional group densities.[^8c^, ^12^]

High‐density nanoparticle‐stabilizing monolayers comprising low molecular‐weight reactive ligands and free from any non‐reactive stabilizer or surfactant components provide the ideal starting point for post‐synthesis modifications of function and properties, guided by robust understanding of the underlying molecular‐level changes.[^2^, ^3^, ^13^] We can achieve this by adopting a “direct synthesis” approach whereby dynamic covalent functionality is incorporated as the only surface‐active ligand during the nanoparticle preparation step.[^2^, ^13^] However, the relatively harsh nanoparticle preparation conditions present a significant challenge when it comes to incorporating highly reactive surface monolayer functionality. We previously accessed high‐density aldehyde‐derived electrophilic monolayers on metal (Au) nanoparticles using hydrazones, which could be modified post synthesis with either nucleophilic hydrazides to achieve dynamic covalent hydrazone exchange, or with an electrophilic scavenger to reveal surface‐bound aldehydes.^[2b]^ Here, we report that introducing the carbonyl species masked as an acetal provides a convenient, rapid and tunable route to dense, single‐component monolayers of nanoparticle‐bound acetals. From these optimized electrophilic nanoparticle starting points, precisely controlled surface densities of aldehydes can be rapidly revealed without requiring addition (and subsequent removal) of scavengers. Exploiting the rich reactivity of aldehydes – which spans highly labile reversible modifications to irreversible transformations – diverse nanoparticle‐bound monolayer constitutions can be easily accessed, including rapidly equilibrating environment‐responsive imines, or instantaneous solubility switching via reversible formation of negatively charged bisulfite adducts. Facile synthetic access to high densities of kinetically labile exchangeable units opens up a new domain of nanoparticle‐bound dynamic covalent reactivity for tuning functionalization, properties and assembly.

## Results and Discussion

**Scheme 1:**
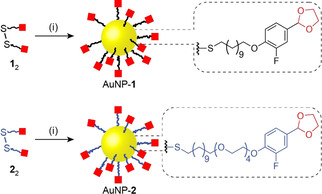
Preparation of acetal‐functionalized dynamic covalent nanoparticles AuNP‐**1** (black monolayer) and AuNP‐**2** (blue monolayer). Red squares represent acetal functional groups. Reagents and conditions: (i) PPh_3_AuCl, *t*‐BuNH_2_⋅BH_3_, BHT, THF/DMF 9 : 1 *v*/*v*, 50 °C, 6 h.

Acetal‐terminated ligands **1** and **2** were designed for stabilizing gold nanoparticles AuNP‐**1** and AuNP‐**2**, respectively (Scheme 1). In each case, a fluorine atom was incorporated to facilitate quantitative characterization of modifications at the monolayer periphery by ^19^F NMR spectroscopy. Starting from the disulfide precursors **1**_2_ or **2**_2_, gold nanoparticles were prepared in one step using a modification of previously reported protocols.[^2a^, ^3^, ^14^] The excellent stability of the acetal‐protected ligands allowed optimization of the reaction temperature and duration to maximize mass recovery. Typically ca. 25 mg purified acetal‐protected AuNP products could be isolated starting from 50 mg gold salt precursor; a significant improvement over recoveries of only ca. 6 mg using the previously reported hydrazone‐stabilized systems on the same reaction scale.[^2a^, ^3^] Imaging by transmission electron microscopy (TEM) revealed low polydispersity monomodal size distributions of <*d*>=5.6±0.5 nm (AuNP‐**1**, Figure S5) and <*d*>=4.9±0.6 nm (AuNP‐**2**, Figure S9). Detailed synthetic protocols can be found in the Supporting Information.

Following nanoparticle isolation and purification by several rounds of precipitation and washing, we obtained AuNP‐**1** and AuNP‐**2**, which formed colloidally stable solutions in a variety of organic solvents. AuNP‐**1** are soluble in CHCl_3_, CH_2_Cl_2_, THF, DMF, H_2_O/DMF (≤4 % *v*/*v* H_2_O) while AuNP‐**2** are soluble in CHCl_3_, CH_2_Cl_2_, acetone, THF, DMF, H_2_O/DMF (≤15 % *v*/*v* H_2_O). In situ characterization of AuNP‐**1** and AuNP‐**2** using ^1^H and ^19^F NMR spectroscopies showed only broad signals corresponding to the resonances expected for a single‐component monolayer of **1** and **2**, respectively (Figures S2, S6). The absence of nonsolvent unbound contaminants was confirmed by *T*_2_‐filtered ^1^H NMR spectroscopy using the CPMG‐*z* pulse sequence (Figures S2, S6).^[15]^ Iodine‐induced oxidative ligand desorption from AuNP‐**1** and AuNP‐**2** generated ^1^H and ^19^F NMR spectra consistent with homogeneous monolayers of **1** and **2**, respectively (Figures S3, S7), confirming the absence of any low‐concentration surface‐bound impurities. The wide chemical shift dispersity of ^19^F NMR spectroscopy provides a particularly sensitive probe of nanoparticle‐bound molecular structure – a critical tool for tracking post‐synthesis transformations (see below) as well as rigorously assessing monolayer structural purity. During optimization of the synthetic protocol, this analytical handle was crucial to identifying and eliminating monolayer impurities to produce a compositionally pure stabilizing monolayer on AuNP‐**1** (see Supporting Information, Section 4). Thermal analysis confirmed a high density of the surface‐bound reactive organic ligands (6.4 ligands nm^−2^ and 6.8 ligands nm^−2^ for AuNP‐**1** and AuNP‐**2**, respectively, Tables S1, S2).

In contrast to the established hydrazone‐functionalized DCNPs,[^2^, ^3^] nanoparticle‐bound acetals are rapidly converted into reactive aldehydes under aqueous acidic conditions, without the need for scavengers and producing only easily removed ethylene glycol as by‐product. The nanoparticle‐bound reaction can be monitored in situ by ^19^F NMR spectroscopy (Figures S12, S13) and arrested at any point, simply by removing or neutralizing the acid catalyst (Figures S14, S15). Thus, from either of the single‐component monolayer starting points, the density of aldehyde functionalization can be tuned precisely and predictably across the full continuum of mixed aldehyde/acetal compositions. This capability confers a powerful level of control over the stoichiometry and proportions of functionalities that can be introduced in subsequent reactions of the nanoparticle‐bound aldehyde reactive sites (see below).

**Figure 1:**
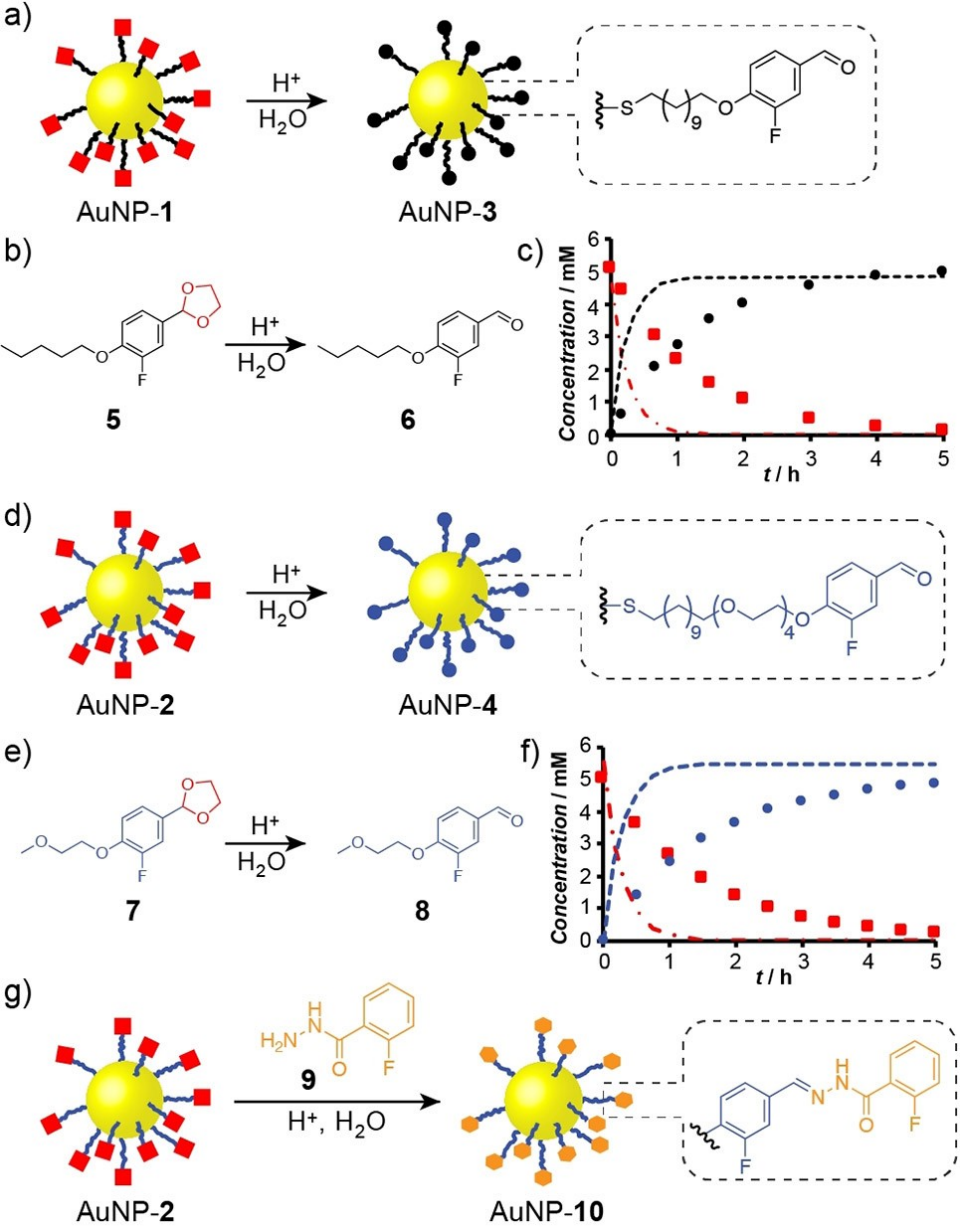
Efficient conversion of acetal‐protected DCNP monolayers to highly reactive aldehyde, or structurally diverse hydrazone DCNP building blocks. a) Acetal deprotection of AuNP‐**1**. b) Acetal deprotection of model compound **5**. c) Representative kinetic profiles for nanoparticle‐bound acetal hydrolysis (AuNP‐**1**, symbols) compared to the analogous reaction in bulk solution (**5**, dashed lines). d) Acetal deprotection of AuNP‐**2**. e) Acetal deprotection of model compound **7**. f) Representative kinetic profiles for nanoparticle‐bound acetal hydrolysis (AuNP‐**2**, symbols) compared to the analogous reaction in bulk solution (**7**, dashed lines). For c), f): Acetal AuNP‐**1** or AuNP‐**2** (red squares, ▪), aldehyde AuNP‐**3** (black circles, •) or AuNP‐**4** (blue circles, •), aldehyde model compounds **5**, **7** (red dot‐dash lines), aldehyde model compounds **6**, **8** (black, blue dashed lines, respectively). For a)–f), conditions: CF_3_CO_2_H (4 equiv. with respect to acetal), D_2_O/DMF 3 : 97 *v*/*v*, 50 °C. g) One‐pot preparation of hydrazone‐functionalized AuNP‐**10** from acetal‐protected AuNP‐**2**. Conditions: hydrazide **9** (1.5 equiv. with respect to **2**), CF_3_CO_2_H (4 equiv. with respect to **2**), D_2_O/DMF 1 : 9 *v*/*v*, r.t., 24 h.

Exhaustive acid‐catalyzed deprotection of acetal‐functionalized AuNP‐**1** and AuNP‐**2** in 3 % D_2_O/DMF at 50 °C rapidly generated homogeneous aldehyde‐functionalized AuNP‐**3** and AuNP‐**4** (Figure 1).^[16]^ Quantitative assessment of reaction kinetics confirmed similar reactivity for both monolayer designs (see Supporting Information, Section 7). Consistent with our previous observations on nanoparticle‐bound reaction kinetics,^[2]^ acetal hydrolysis is significantly retarded (by a factor of ca. ×0.18) compared to the solution‐phase hydrolysis of analogous model compounds **5** and **7** (Figure 1 and Table S3). The more hydrophilic AuNP‐**2** remain colloidally stable at higher concentrations of water, allowing even faster deprotection and direct comparison with the previously reported hydrazone systems:^[2b]^ full conversion of AuNP‐**2** was achieved in 10 % D_2_O/DMF after only 100 min, a remarkable improvement over the previous hydrazone‐protected DCNPs for which exhaustive hydrolysis required >24 h in the presence of an electrophilic scavenger species that must subsequently be removed.^[16]^ Quantitative kinetic analysis (see Supporting Information, Section 7) revealed that nanoparticle‐bound acetal hydrolysis kinetics follow a first‐order dependence on the concentrations of both water and acid. Thus, deprotection could also be achieved within reasonable timescales under milder conditions of lower acid catalyst concentration (Figure S19, Table S5).

Nanoparticle‐bound acetals proved to be highly stable in the absence of acid catalyst – AuNP‐**1**/**2** showing no change on storage as a solid in air for at least 15 months. Furthermore, following deprotection, aldehyde‐functionalized AuNP‐**3**/**4** remained stable in the reaction solution (D_2_O/DMF 3 : 97 *v*/*v*, 20 mM CF_3_CO_2_H) for at least 20 days.

We have demonstrated the programmable dynamic reactivity and broad structural diversity accessible for hydrazone DCNPs.^[2]^ Previously, direct synthesis of a very small number of hydrazone DCNP building blocks has granted access to all derivatives of this family in a highly divergent manner. Acetals, however, are less sensitive to reducing conditions and elevated temperatures, offering increased scope for optimizing nanoparticle preparation conditions (see above) or transfer to alternative core materials. The flexibility of reversible hydrazone conjugation at electrophilic carbonyl centers, and the ready availability of a wide structural variety of simple hydrazide modifier units, as we exemplified in our previous approaches,^[2]^ are both directly transferrable to acetal‐protected building blocks. Any number of hydrazone‐functionalized monolayers may be accessed by reacting the appropriate hydrazide modifier with either the deprotected aldehyde DCNP (for example, reaction of AuNP‐**3** with 2‐fluorobenzohydrazide **9**, Figures S21, S22), or directly from the acetal‐protected building blocks in a one‐pot process. For example, AuNP‐**2** reacted with 2‐fluorobenzohydrazide **9** to produce hydrazone‐functionalized AuNP‐**10** (Figure 1g). As for the hydrolysis reaction, tracking the nanoparticle‐bound conversion of acetal to hydrazone by in situ ^19^F NMR spectroscopy (for example, Figure S20) enables precise control over the extent of functionalization, giving access to any intermediate mixed‐ligand monolayer composition.^[17]^ Although this one‐pot process is operationally simple, an advantage of the two‐step route is that no water is required for the conjugation to nanoparticle‐bound aldehydes AuNP‐**3**/**4**, which extends the scope of compatible hydrazide functionality.

Even more attractive are the opportunities offered by AuNP‐**3** and AuNP‐**4** for accessing the rich dynamic reactivity of aldehydes within a dense well‐defined nanoparticle‐stabilizing monolayer. We are particularly interested in adaptive systems that can be created using rapidly equilibrating reactions of labile functional groups, but which were not accessible directly from the previous hydrazone‐terminated building blocks.

Aldehyde‐functionalized DCNPs can react with primary amines to generate imine‐functionalized monolayers. Being highly sensitive to environmental parameters, imines are ideal for adaptive tuning of surface monolayer composition – and hence physicochemical and functional characteristics – in response to external stimuli.^[18]^ Over a number of years, Lehn and coworkers have explored the constitutionally dynamic behavior of solution‐phase imine libraries in response to a number of environmental parameters, including acidity and temperature.^[19]^ More recently, pioneering studies have examined imine exchange reactions taking place at interfaces using fluorescence or probe microscopies.[^18^, ^20^] Our densely functionalized aldehyde DCNP platforms allow direct interrogation of the surface‐bound mixtures by techniques that combine molecular structural detail with ensemble measurement (for example, NMR spectroscopy) while avoiding indirect reporters or derivatization protocols such as imine reduction that risk skewing the library composition.

**Figure 2:**
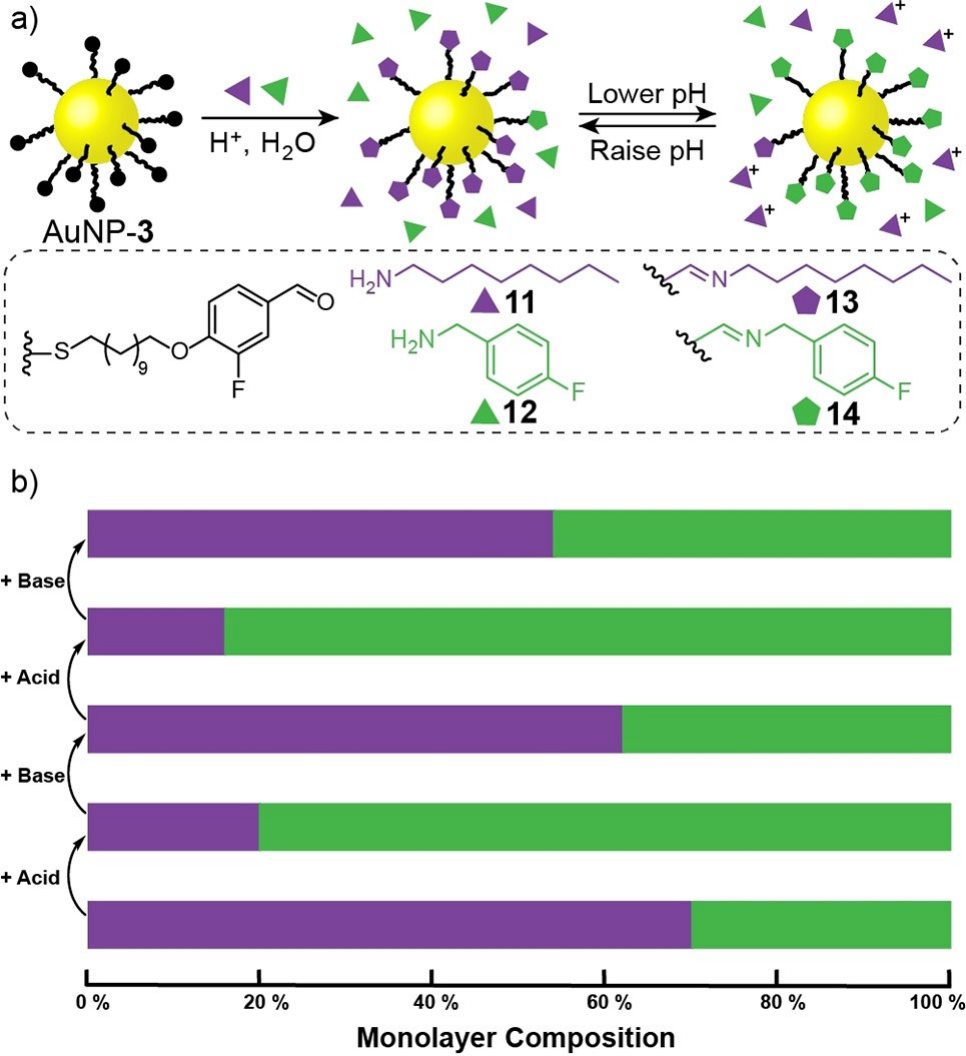
a) Rapidly adaptive monolayer constitutions based on environment‐responsive imine functionalization of AuNP‐**3**. b) Constitutional reorganization of imine‐functionalized monolayers in response to acid and base stimuli. Imine formation conditions: CF_3_CO_2_H (0.1 equiv. with respect to **3**), amine **11** (2.0 equiv. with respect to **3**), amine **12** (2.0 equiv. with respect to **3**), DMF/THF 1 : 1 *v*/*v*, 50 °C, 38 h. pH‐Responsive compositional switching conditions: ‘Acid’=CF_3_CO_2_H (2.5 equiv. with respect to **3**); ‘Base’=1,8‐diazabicyclo[5.4.0]undec‐7‐ene (2.5 equiv. with respect to **3**), DMF/THF 1 : 1 *v*/*v*, 50 °C, 1 h.

Treating AuNP‐**3** with octylamine **11** and 4‐fluorobenylamine **12** produced mixed‐ligand imine‐functionalized DCNPs AuNP‐**13**_x_ **14**_y_ (Figure 2). In situ analysis by ^19^F NMR spectroscopy revealed the mixed‐ligand monolayer composition in the presence of equimolar quantities of **11** and **12** to be AuNP‐**13**_0.7_ **14**_0.3_ (Figure 2b),^[17]^ reflecting the higher nucleophilicity of aliphatic amine **11**. Constitutional reorganization of the nanoparticle‐bound monolayer could be induced by simple chemical stimuli (acids and bases). On adding acid, the more basic amine **11** is more readily protonated, dramatically reducing its nucleophilicity, resulting in a shift in monolayer composition to favor the imine of less basic amine **12** (AuNP‐**13**_0.2_ **14**_0.8_). Compositional switching is entirely reversible – the initial monolayer composition is restored on neutralizing the acid stimulus with base, and the switching process repeatable on subsequent acid‐base cycles (Figure 2b). Comparison to solution‐phase experiments on model aldehyde **6** (see Supporting Information, Section 11) indicates that, in contrast to the kinetic effects observed above, surface confinement of the aldehyde component does not affect the thermodynamically controlled composition of dynamic imines.

**Figure 3:**
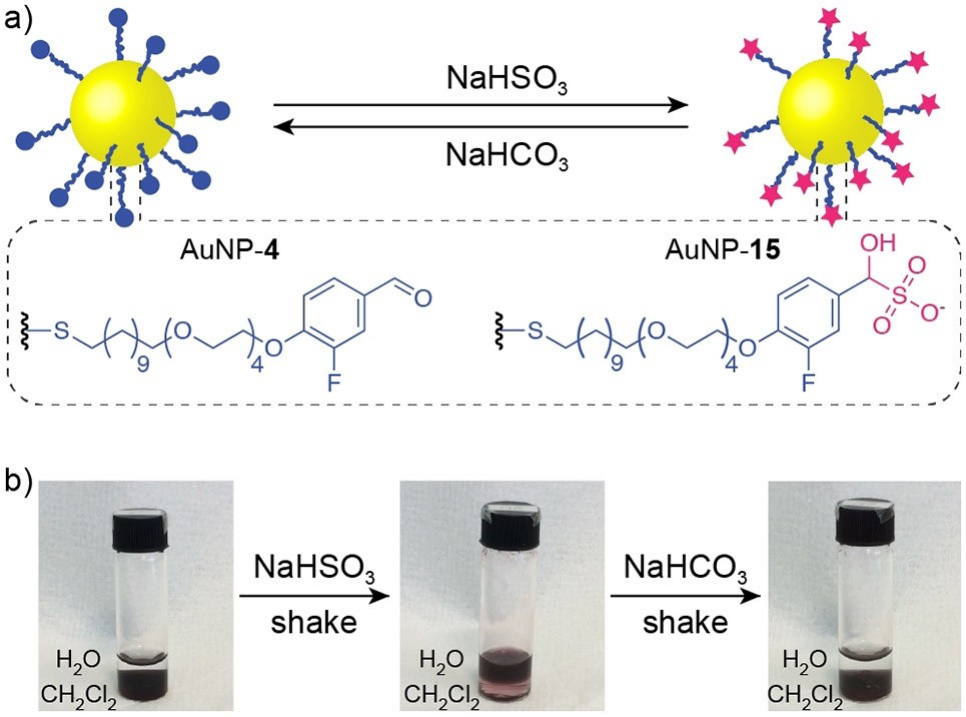
Rapid solvophilicity switching via reversible formation of nanoparticle‐bound bisulfite adducts. a) Interconversion between hydrophobic neutral AuNP‐**4** and hydrophilic negatively charged bisulfite adduct AuNP‐**15**. b) Reversible phase transfer between dichloromethane and water on sequential treatment with NaHSO_3_ then NaHCO_3_.

Inspired by the traditional derivatization method for purifying hydrophobic aldehydes via reversible formation of water‐soluble bisulfite adducts,^[21]^ we explored rapid and reversible solubility switching of aldehyde‐functionalized AuNP‐**4** (Figure 3a). These nanoparticles are entirely insoluble in water, but adding solid NaHSO_3_ (2 mg per 1 mg of AuNPs) to a precipitate of AuNP‐**4** in D_2_O followed by agitation (5 min sonication) resulted in dissolution of all nanoparticle material. Analysis by ^1^H NMR spectroscopy (Figure S26) indicated quantitative formation of negatively charged bisulfite adduct AuNP‐**15**. Rapid decomposition back to the starting aldehyde was achieved on addition of solid NaHCO_3_ (4 mg per 1 mg of AuNPs), resulting in re‐precipitation of all nanoparticle material after 5 min. After washing with water to remove the excess salts, the resulting black solid was solubilized in CDCl_3_ and shown to be spectroscopically identical to the starting material (Figure S26).

In a biphasic system of dichloromethane and water, nanoparticle phase switching could be achieved rapidly by reversible bisulfite adduct formation (Figure 3b). In the aldehyde state, hydrophobic AuNP‐**4** are soluble in the organic phase only. On adding solid NaHSO_3_ to the aqueous phase, followed by brief agitation (shaking by hand, 2 min), quantitative transfer of nanoparticles into the aqueous phase was observed. Subsequently, adding solid NaHCO_3_ and again shaking (5 min) promoted transfer of all nanoparticle material back to the organic layer. Although post‐synthesis modification of nanoparticle solvophilicity has previously been achieved using either host–guest chemistry^[22]^ or dynamic covalent exchange,[^2a^, ^3^] irreversible switching mechanisms, slow kinetics (hours to days) or trapping at the biphasic interface tend to hamper rapid and reversible phase transfer in biphasic systems.^[23]^ Therefore, fast and reversible solubility switching, requiring only simple inorganic salts, is attractive for a variety of practical applications.^[23]^

## Conclusions

We have demonstrated that acetal protection overcomes the incompatibility of sensitive carbonyl functionalities with the harsh conditions required for AuNP preparation. This new ligand design enables optimized direct synthesis of metallic nanoparticles stabilized by a high density of reactive electrophilic surface ligands. On‐nanoparticle acetal hydrolysis is rapid under mild, scavenger‐free conditions, generating only easily removed by‐products. A spectroscopic handle facilitates in situ real‐time monitoring, allowing the surface density of reactive aldehyde units to be precisely and reliably tuned across a broad continuum. These synthetic and analytical advantages can subsequently be applied to a wide variety of transformations for nanoparticle surface engineering, giving full constitutional control over both structure and composition of any number of mixed‐ligand monolayers using structurally simple modifier units. This contrasts with alternative approaches including ligand exchange for which often only a subset of mixed‐ligand compositions are accessible after system‐specific optimization and preparation of pro‐ligand structures in their entirety.

The kinetic stability and on‐demand reactivity of acetals, allied with the rich reversible and irreversible covalent reactivity of aldehydes, endow this DCNP building block with the characteristics to access a huge range of complementary and orthogonal modifications. The programmable options span labile stimuli‐responsive reversible transformations, to completely irreversible changes – all with precise control over the composition of mixed‐ligand monolayers. Revealing highly reactive surface‐bound aldehydes gives access to rapidly adaptive nanoparticle‐stabilizing monolayers, which were previously only accessible at low functionalization densities. Combined with the analytical tractability of this high‐density colloidally stable nanoparticle platform, we can directly probe the constitutional dynamics of adaptive surface‐bound imine libraries at the ensemble level. Alternatively, rapid and reversible nanoparticle phase transfer between organic and aqueous solvents is achieved using only simple inorganic salts.

Prepared via optimized operationally simple protocols, stable to long‐term storage, requiring no activation prior to use, acetal‐protected AuNPs represent the idealized DCNP building block. Transformations at the monolayer periphery should be independent of the nanoparticle core, and thus this strategy should be generalizable for efficient divergent functionalization of a variety of colloidal nanomaterials using covalent exchange processes spanning a remarkably wide range of reactivities, guided by in situ spectroscopic tracking. These building blocks will open up a new domain of nanoparticle‐bound dynamic covalent reactivity for nanoparticle surface engineering applicable to a wide range of nanomaterials applications.^[24]^

## Conflict of interest

The authors declare no conflict of interest.

## Supporting information

As a service to our authors and readers, this journal provides supporting information supplied by the authors. Such materials are peer reviewed and may be re‐organized for online delivery, but are not copy‐edited or typeset. Technical support issues arising from supporting information (other than missing files) should be addressed to the authors.

Supplementary
