# Supplementary material for: Rapidly Adaptive All‐covalent Nanoparticle Surface Engineering
Source: Chemistry. 2021 May 21;27(38):9948–53. doi: 10.1002/chem.202101042 (PMC8362155; doi:10.1002/chem.202101042)
Supplement: Supplementary file 1 — Supplementary [file CHEM-27-9948-s001.pdf]

Chemistry–A European Journal

Supporting Information

Rapidly Adaptive All-covalent Nanoparticle Surface Engineering

Marta Diez-Castellnou[+], Rongtian Suo[+], Nicolas Marro, Saphia A. L. Matthew, and Euan R. Kay*

Table of contents

1. General experimental procedures S1
2. Synthesis of molecular species S2
3. Synthesis and characterization of gold nanoparticles S9
   3.1 AuNP-**1** S9
   3.2 AuNP-**2** S13
4. Identification and elimination of acyclic acetal monolayer impurity S17
5. Exhaustive and partial on-nanoparticle acetal hydrolysis S19
   5.1 Exhaustive acetal hydrolysis from AuNP-**1** S19
   5.2 Exhaustive acetal hydrolysis from AuNP-**2** S20
   5.3 Partial acetal hydrolysis from AuNP-**1** S21
   5.4 Partial acetal hydrolysis from AuNP-**2** S22
6. Hydrolysis of acetal model compounds S24
   6.1 Hydrolysis of acetal model compound **5** S24
   6.2 Hydrolysis of acetal model compound **7** S25
7. Kinetic studies of acetal hydrolysis S26
   7.1 Experimental protocol for kinetic experiments S26
   7.2 Summary of kinetic data S27
8. One-step dynamic covalent modification of acetal-functionalized nanoparticles with nucleophilic modifiers S29
9. Two-step dynamic covalent modification of acetal-functionalized nanoparticles with nucleophilic modifiers S31
10. Imine constitutional reorganization on AuNP-**3** S33
11. Imine constitutional reorganization in model system S35
12. Generation and characterization of negatively charged AuNP-**15** S38
    12.1 Rapid reversible solvophilicity switching in one phase S38
    12.2 Rapid reversible biphasic switching S38
    12.3 NMR characterization of reversible biphasic switching S39
13. ^{1}H and ^{13}C NMR spectra of organic compounds S40
14. Supplementary references. S52

1. General experimental procedures

Unless stated otherwise, all reagents were purchased from commercial sources and used without further purification. Dry solvents were obtained by means of a MBRAUN MB SPS-800TM solvent purification system, where solvents were passed through filter columns and dispensed under an argon atmosphere. Flash column chromatography was performed using Geduran® Si60 (40–63 μm, Merck, Germany) as the stationary phase. Thin-layer chromatography (TLC) was performed on pre-coated silica gel plates (0.25 mm thick, 60F254, Merck, Germany) and observed under UV light (λ_{max} 254 nm) or visualized by staining with a basic potassium permanganate solution, followed by heating. Nanoparticle micrographs were obtained using a JEM 2010 transmission electron microscope (TEM) on samples prepared by deposition of one drop of nanoparticle suspension on holey carbon films supported on a 300 mesh Cu grid (Agar Scientific®). Nanoparticle diameters were measured automatically using the software ImageJ. The images were first converted to black and white images using the "Threshold" function; the area of each nanoparticle was measured using the "Analyze particles" function; particles on edges were excluded. ^{1}H, ^{13}C, and ^{19}F NMR spectra were recorded on Bruker Avance II 300, 400 and 500 instruments, at a constant temperature of 25 °C. All ^{19}F and ^{13}C spectra were recorded using proton decoupling. Chemical shifts are reported in parts per million (ppm) from low to high field; ^{1}H and ^{13}C chemical shifts are referenced to the literature values for chemical shifts of residual non-deuterated solvent, with respect to tetramethylsilane;^{19}F Chemical shifts are referenced to CF_3Cl (0.00 ppm) as external standard. Standard abbreviations indicating multiplicity are used as follows: bs (broad singlet), d (doublet), dd (doublet of doublets), dt (doublet of triplets), m (multiplet), s (singlet), t (triplet), tt (triplet of triplets), q (quartet), quint (quintuplet), J (coupling constant). All spectra were analyzed using MestReNova (Version 9.0.0–12.0.02). All melting points were determined using a Stuart SMP30 Melting Point Apparatus and are reported uncorrected. A Beckman Coulter Avanti J-25 centrifuge was used equipped with the JA-25.50 rotor. Thermogravimetric analysis is performed using a Staton Redcroft STA-780 simultaneous TG-DTA: room temperature to 900 ºC, which is connected to a Rheometric Scientific STA SID System Interface.

2. Synthesis of molecular species

Chloro(triphenylphophine)gold(I), **S1**, **S2**, **S5** and **S6** were prepared as previously described.[1]

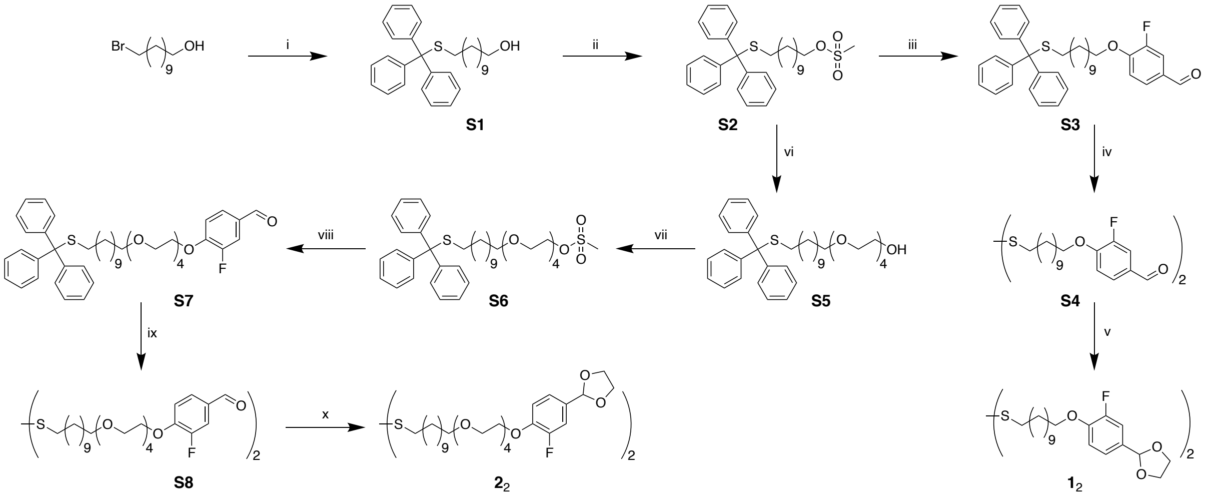

Scheme S1. Summary of synthetic procedures. Reagents and conditions: (i) Triphenylmethanethiol, NaOH, H_2O, EtOH, toluene, r.t., 3.5 h, 94%; (ii) methanesulfonyl chloride, Et_3N, CH_2Cl_2, 0 °C / r.t., 4.5 h, 93%; (iii) 3-fluoro-4-hydroxybenzaldehyde, K_2CO_3, KI, DMF, 80 °C, overnight, 56%; (iv) I_2, CH_2Cl_2, r.t., 2 h, 76%; (v) ethylene glycol, *p*-toluenesulfonic acid, 80 °C, reduced pressure 2.5 h, 65%; (vi) tetraethylene glycol, H_2O, 90 °C, 18 h, 82%; (vii) methanesulfonyl chloride, Et_3N, CH_2Cl_2, 0 °C / r.t., 4.5 h, 75%; (viii) 3-fluoro-4-hydroxybenzadehyde, K_2CO_3, DMF, 90 °C, 16 h, 91; (ix) I_2, MeOH, rt, 1 h, 99%; (x) ethyleneglycol, *p*-toluenesulfonic acid, 80 °C, reduced pressure, 2 h, 88%.

3-Fluoro-4-((11-(tritylthio)undecyl)oxy)benzaldehyde (S3)

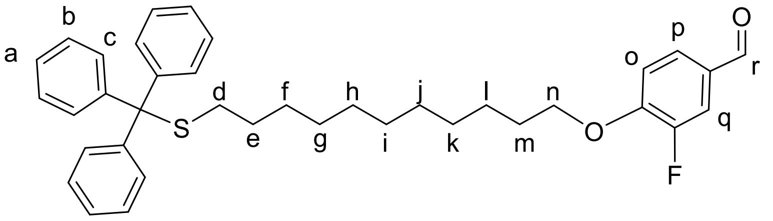

3-Fluoro-4-hydroxybenzaldehyde (1.42 g, 10.1 mmol), K_2CO_3 (4.19 g, 30.3 mmol) and KI (1.17 g, 7.08 mmol) were placed in a two-necked round-bottomed flask. The system was flushed with argon, and DMF (60 mL) was added to the flask before the temperature was raised to 80 °C. Compound **S2** dissolved in DMF (10 mL) was added dropwise to the reaction mixture, which was then left stirring overnight (21 h). Once the reaction had gone to completion (TLC, cyclohexane/EtOAc 5:1), the solvent was removed under reduced pressure and the crude product was re-dissolved in EtOAc (150 mL) then washed with water (1 × 150 mL) and brine (3 × 50 mL). The organic layer was dried over $MgSO_4$ then volatiles removed under reduced pressure. The crude product was purified by flash column chromatography (SiO_2, cyclohexane/EtOAc 5:1) to give **S3** as a pale-yellow oil (3.04 g, yield: 56%).

^{1}H NMR (400 MHz, $CDCl_3$) δ 9.85 (d, *J* = 2.1 Hz, 1H, r), 7.64–7.58 (m, 2H, p, q), 7.44–7.38 (m, 6H, b), 7.31–7.24 (m, 6H, c), 7.23–7.17 (m, 3H, a), 7.05 (t, *J* = 8.1 Hz, 1H, o), 4.10 (t, *J* = 6.6 Hz, 2H, n), 2.13 (t, *J* = 7.3 Hz, 2H, d), 1.85 (dt, *J* = 14.6, 6.7 Hz, 2H, m), 1.52–1.09 (m, 16H, e, f, g, h, i, j, k, l).

^{13}C NMR (126 MHz, $CDCl_3$) δ 190.0, 152.6 (d, *J* = 249.1 Hz), 152.6 (d, *J* = 10.7 Hz), 145.1, 129.8 (d, *J* = 5.8 Hz), 129.6, 127.8, 126.5, 126.5 115.7 (d, *J* = 18.8 Hz), 113.5, 69.5, 66.4, 32.1, 29.5, 29.5, 29.5, 29.5, 29.4, 29.3, 29.0, 28.6, 25.9.

^{19}F NMR (470 MHz, $CDCl_3$) δ –133.0.

HRMS (ESI$^+$) *m/z* calculated for $C_{37}H_{41}FO_2S$ $[M+H]^+$ 591.2704, found 591.2694.

4,4'-((Disulfanediylbis(undecane-11,1-diyl))bis(oxy))bis(3-fluorobenzaldehyde) (S4)

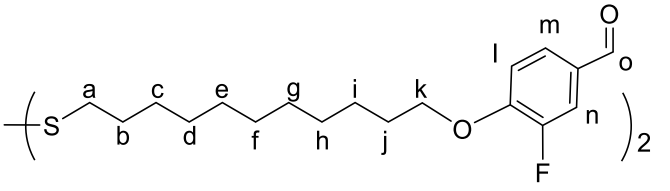

Compound **S3** (3.04 g, 5.34 mmol) and iodine (1.76 g, 6.94 mmol) were dissolved in CH_2Cl_2 (90 mL) in a round-bottomed flask. The reaction mixture was stirred at room temperature and monitored using TLC (cyclohexane/EtOAc 5:1). As soon as the starting material was consumed (2 h after adding iodine), the reaction was stopped by washing with a saturated aqueous solution of $NaHSO_3$ (70 mL) to remove the excess of iodine. The aqueous layer was extracted with CH_2Cl_2 (3 × 90 mL). The organic layers were then combined and dried over $MgSO_4$ and solvent was removed under reduced pressure. The crude product was purified by flash column chromatography (SiO_2, cyclohexane/EtOAc 5:1 to 3:1) to obtain **S4** as a white solid (1.32 g, yield: 76%).
^{1}H NMR (500 MHz, $CDCl_3$) δ 9.85 (d, *J* = 2.1 Hz, 2H, o), 7.64–7.58 (m, 4H, m, n), 7.05 (t, *J* = 8.2 Hz, 2H, l), 4.11 (t, *J* = 6.6 Hz, 4H, k), 2.68 (t, 4H, *J* = 7.3 Hz, a), 1.89–1.82 (m, 4H, j), 1.70–1.62 (m, 4H, b), 1.51–1.44 (m, 4H, i), 1.55–1.24 (m, 24H, c, d, e, f, g, h).
^{13}C NMR (126 MHz, $CDCl_3$) δ 190.0, 153.7 (d, *J* = 249.8 Hz), 152.8 (d, *J* = 10.9 Hz), 129.9 (d, *J* = 5.3 Hz), 128.2 (d, *J* = 3.0 Hz), 115.98 (d, *J* = 19.1 Hz), 113.6, 69.6, 39.3, 29.6, 29.6, 29.6, 29.6, 29.3, 29.3, 29.0, 28.6, 26.0.
^{19}F NMR (470 MHz, $CDCl_3$) δ –133.0.
HRMS (ESI$^+$) *m/z* calculated for $C_{36}H_{52}F_2O_4S_2$ $[M+H]^+$ 651.3348, found 651.3340.

1,2-Bis(11-(4-(1,3-dioxolan-2-yl)-2-fluorophenoxy)undecyl)disulfane (1$_2$)

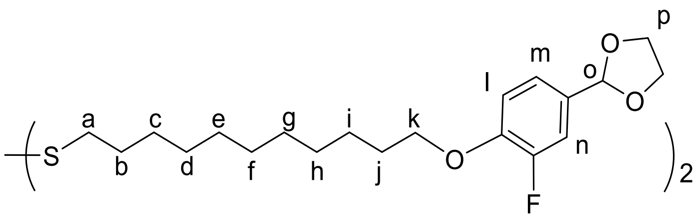

Compound **S4** (1.24 g, 1.90 mmol) was dissolved in CH_2Cl_2 (10 mL) in a round-bottomed flask. The solvent was removed under reduced pressure to leave an oil. Ethylene glycol (45 mL) was added to the flask, the mixture stirred to give a suspension, and then *p*-toluenesulfonic acid (0.289 g, 1.52 mmol) added. The flask was heated to 80 °C in the water bath of a rotary evaporator and carefully evacuated for short periods to remove water while retaining ethylene glycol. The reaction was monitored by ^{1}H and ^{19}F NMR spectroscopy. When the reaction had reached completion (2.5 h), the solution was dissolved in CH_2Cl_2 (200 mL) and washed with saturated aqueous $NaHCO_3$ (3 × 150 mL) and brine (3 × 150 mL). The organic layer was dried over $MgSO_4$, then volatiles removed under reduced pressure to obtain **1**$_2$ as a white solid (0.910 g, yield: 65%).
^{1}H NMR (400 MHz, $CDCl_3$) δ 7.21 (dd, *J* = 11.9, 2.0 Hz, 2H, m), 7.18–7.13 (m, 2H, n), 6.94 (t, *J* = 8.3 Hz, 2H, l), 5.76 (s, 2H, o), 4.15–4.07 (m, 4H, k), 4.06–3.97 (m, 8H, p), 2.68 (t, *J* = 7.5 Hz, 4H, a), 1.85–1.76 (m, 4H, j), 1.71–1.62 (m, 4H, b), 1.51–1.22 (m, 28H, c, d, e, f, g, h, i).
^{13}C NMR (126 MHz, $CDCl_3$) δ 152.6 (d, *J* = 248.3 Hz), 148.0 (d, *J* = 10.9 Hz), 130.9 (d, *J* = 5.4 Hz), 122.6 (d, *J* = 3.5 Hz), 115.8 (d, *J* = 19.4 Hz), 114.5 (d, *J* = 19.7 Hz), 114.4, 103.1, 69.6, 65.4, 65.4, 39.3, 29.6, 29.5, 29.5, 29.4, 29.3, 29.1, 28.7, 26.0.
^{19}F NMR (377 MHz, $CDCl_3$) δ –134.2.
HRMS (ESI$^+$) *m/z* calculated for $C_{40}H_{60}F_2O_6S_2$ $[M+H]^+$ 739.3872, found 739.3872.

3-Fluoro-4-((1,1,1-triphenyl-14,17,20,23-tetraoxa-2-thiapentacosan-25-yl)oxy)benzaldehyde (S7)

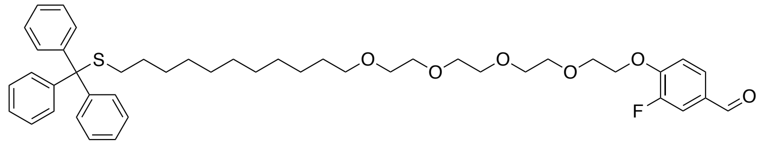

To a solution of 3-fluoro-4-hydroxybenzaldehyde (910 mg, 6.49 mmol) in DMF (40 mL) in a round-bottom flask, K_2CO_3 (2.07 g, 15.0 mmol) and KI (580 mg, 3.50 mmol) were added. The mixture was stirred for 40 min at 90 °C, then a solution of mesylate **S6** (3.50 g, 4.99 mmol) in DMF (10 mL) was added dropwise and stirring continued at 90 °C for 16 h. Reaction progress was monitored by TLC and on completion, the mixture was allowed to cool to room temperature. DMF was removed under reduced pressure. The residue was dissolved in ethyl acetate (100 mL) and washed with brine (2 x 50 mL). The organic layer was dried over $MgSO_4$, filtered and evaporated under reduced pressure. The crude oil was purified by flash column chromatography (SiO_2, ethyl acetate/ petroleum ether 1:1 to 3:1) to afford **S7** as a colourless oil (3.72 g, 91%).

^{1}H NMR (500 MHz, $CDCl_3$): δ 1.05–1.30 (m, 14H, 7 x CH_2), 1.38 (quint, *J* = 7.5 Hz, 2H, CH_2), 1.56 (quint, *J* = 7.2 Hz, 2H, CH_2), 2.12 (t, *J* = 7.4 Hz, 2H, CH_2S), 3.43 (t, *J* = 6.9 Hz, 2H, CH_2O), 3.57 (t, *J* = 5.4 Hz, 2H, CH_2O), 3.60–3.69 (m, 8H, 4 x CH_2O), 3.74 (t, *J* = 5.2 Hz, 2H, CH_2O), 3.92 (t, *J* = 4.8 Hz, 2H, CH_2O), 4.28 (t, *J* = 4.6 Hz, 2H, CH_2O), 7.10 (t, *J* = 8.3 Hz, 1H, HAr), 7.20 (m, 2H, HAr), 7.24–7.32 (m, 6H, HAr), 7.37–7.44 (m, 6H, HAr), 7.5–7.64 (m, 2H, HAr), 9.85 (bs, 1H, CHO) ppm.

^{13}C NMR (100.6 MHz, $CDCl_3$): δ 26.2, 28.7, 29.2, 29.3, 29.5, 29.6, 29.7, 29.8, 32.2, 66.5, 69.1, 69.5, 70.2, 70.7, 70.8, 71.2, 71.7, 114.1, 115.9, 116.0, 126.6, 127.4, 127.9, 128.1, 128.1, 128.2, 128.2, 145.2, 147.0, 151.8, 152.5, 152.6, 153.8, 190.1 ppm.

^{19}F NMR (470.6 MHz, $CDCl_3$): δ –132.77 (s, 1F) ppm.

HRMS (ESI$^+$) m/z calculated for $C_{45}H_{57}FNaO_6S$ $[M+Na]^+$ 767.3752, found 767.3733.

4,4'-((3,6,9,12,37,40,43,46-Octaoxa-24,25-dithiaoctatetracontane-1,48-diyl)bis(oxy))bis-(3-fluorobenzaldehyde) (S8)

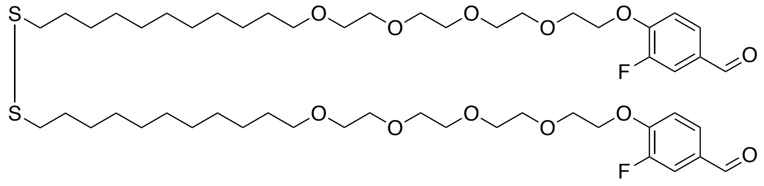

Iodine (1.27 g, 5.02 mmol) was added to a solution of compound **S7** (3.40 g, 4.56 mmol) in a mixture of MeOH and DCM (3:1 *v*/*v*, 120 mL). The mixture was stirred at rt for 1 h. Then, the solution was quenched by adding a saturated solution of $NaHSO_3$, and extracted with CH_2Cl_2 (3 x 100 mL). The organic layers were dried over $MgSO_4$, filtered and evaporated under reduced pressure. The crude oil was purified by flash column chromatography (SiO_2, CH_2Cl_2/MeOH 2%) to afford **S8** as a colourless oil (2.35 g, 99%).

^{1}H NMR (500.1 MHz, $CDCl_3$): δ 1.22–1.40 (m, 28H, 14 x CH_2), 1.52–1.62 (m, 4H, 2 x CH_2), 1.62–1.70 (m, 4H, 2 x CH_2), 2.67 (t, *J* = 7.35 Hz, 4H, 2 x CH_2S), 3.43 (t, *J* = 6.8 Hz, 4H, 2 x CH_2O), 3.57 (t, *J* = 5.4 Hz, 4H, 2 x CH_2O), 3.62–3.69 (m, 16H, 12 x CH_2O), 3.74 (t, *J* = 5.3 Hz, 4H, 2 x CH_2O), 3.92 (t, *J* = 4.9 Hz, 4H, 2 x CH_2O), 4.29 (t, *J* = 4.7 Hz, 4H, 2 x CH_2O), 7.10 (t, *J* = 8.3 Hz, 2H, HAr), 7.58–7.64 (dt, *J* = 10.8, 4H, HAr), 9.85 (bs, 2H, CHO) ppm.

^{13}C NMR (125.8 MHz, $CDCl_3$): δ 26.2, 28.7, 29.6, 29.4, 29.6, 29.7, 29.7, 29.7, 29.8, 39.3, 69.1, 69.5, 70.2, 70.7, 70.8, 71.2, 71.7, 114.1, 115.9, 116.0, 128.2, 128.2, 130.3, 130.3, 151.8, 152.5, 152.6, 153.8, 190.1 ppm.

^{19}F NMR (470.6 MHz, $CDCl_3$): δ –132.77 (s, 2F) ppm.

HRMS (ESI$^+$) m/z calculated for $C_{52}H_{84}F_2NaO_{12}S_2$ $[M+Na]^+$ 1025.5264, found 1025.5241.

1,48-Bis(4-(1,3-dioxolan-2-yl)-2-fluorophenoxy)-3,6,9,12,37,40,43,46-octaoxa-24,25-di-thiaoctatetracontane (2_2)

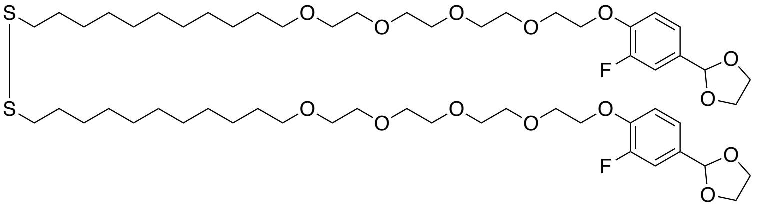

p-Toluenesulfonic acid (133 mg, 700 µmol) was added to a solution of compound **S8** (1.00 g, 1.00 mmol) in ethyleneglycol (50 mL). The reaction mixture was heated to 80 °C under reduced pressure (approx. 10 mbar) for 2 h. The reaction was then cooled to room temperature and extracted with CH_2Cl_2 containing Et_3N (3 x 50 mL). The organic layers were dried over $MgSO_4$, filtered and evaporated under reduced pressure to give product **2**$_2$ as a colourless oil (1.09 g, 88%).
^{1}H NMR (500.1 MHz, $CDCl_3$): δ 1.22–1.40 (m, 28H, 14 x CH_2), 1.52–1.62 (m, 4H, 2 x CH_2), 1.62–1.70 (m, 4H, 2 x CH_2), 2.67 (t, *J* = 7.4 Hz, 4H, 2 x CH_2S), 3.43 (t, *J* = 6.9, 4H, 2 x CH_2O), 3.55–3.58 (m, 4H, 2 x CH_2O), 3.62–3.68 (m, 16H, 8 x CH_2O, 3.71–3.74 (m, 4H, 2 x CH_2O), 3.87 (t, *J* = 5.1, 4H, 2 x CH_2O), 4.01 (m, 4H, 2 x CH_2O), 4.11 (m, 4H, 2 x CH_2O), 4.19 (t, *J* = 4.8, 4H, 2 x CH_2O), 5.73 (s, 2H, 1 x CH), 6.97 (t, *J* = 8.3, 2H, HAr), 7.16 (d, *J* = 8.5 Hz, 2H, HAr), 7.21 (dd, *J* = 11.7, 2H, HAr) ppm.
^{13}C NMR (125.8 MHz, $CDCl_3$): δ 26.2, 28.7, 29.4, 29.4, 29.6, 29.6, 29.7 29.7, 29.8 29.8, 39.3, 65.4, 69.2, 69.7, 70.2, 70.7, 70.7, 71.1, 71.7, 103.0, 114.5, 114.6, 115.0, 122.7, 122.7, 129.0, 131.1, 131.5, 147.7, 147.7, 151.7, 153.7 ppm
^{19}F NMR (470.6 MHz, $CDCl_3$): δ –133.95 (s, 2F) ppm.
HRMS (ESI$^+$) m/z calculated for $C_{52}H_{84}F_2NaO_{12}S_2$ $[M+Na]^+$ 1113.5789, found 1113.5777.

3-Fluoro-4-(pentyloxy)benzaldehyde (6)

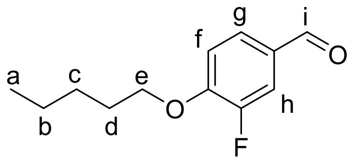

3-Fluoro-4-hydroxybenzaldehyde (1.95 g, 13.9 mmol), K_2CO_3 (5.76 g, 41.7 mmol) and KI (1.62 g, 9.76 mmol) were placed in a two-necked round-bottomed flask. The system was flushed with argon, and DMF (85 mL) was added to the flask before the temperature was raised to 80 ºC. 1-Bromopentane (1.65 mL, 2.00 g, 13.2 mmol) dissolved in DMF (15 mL) was added dropwise to the reaction mixture, and the reaction left to stir for 20 h. Once the reaction had gone to completion (TLC, cyclohexane/CH_2Cl_2 1:2), the solvent was removed under reduced pressure and the residue was re-dissolved in EtOAc (200 mL) then washed with water (1 × 200 mL) and brine (3 × 70 mL). The organic layer was dried over $MgSO_4$ and volatiles were removed under reduced pressure. The crude product was purified by flash column chromatography (SiO_2, cyclohexane/CH_2Cl_2 1:2) to give **6** as a pale-yellow oil (1.49 g, yield: 54%).
^{1}H NMR (400 MHz, $CDCl_3$) δ 9.85 (d, *J* = 2.1 Hz, 1H, i), 7.63–7.58 (m, 2H, g, h), 7.06 (t, *J* = 8.2 Hz, 1H, f), 4.11 (t, *J* = 6.6 Hz, 2H, e),1.87 (m, 2H, d), 1.51–1.35 (m, 4H, b, c), 0.94 (t, *J* = 7.2 Hz, 3H, a).
^{13}C NMR (101 MHz, $CDCl_3$) δ 190.1, 152.7 (d, *J* = 249.4 Hz), 152.9 (d, *J* = 11.0 Hz), 129.8 (d, *J* = 5.2 Hz), 128.3 (d, *J* = 3.0 Hz), 115.7 (d, *J* = 18.2 Hz), 113.6, 69.6, 28.7, 28.1, 22.5, 14.1.
^{19}F NMR (377 MHz, $CDCl_3$) δ –133.0.
HRMS (ESI$^+$) *m/z* calculated for $C_{12}H_{15}FO_2$ $[M+H]^+$ 211.1129, found 211.1129.

2-(3-Fluoro-4-(pentyloxy)phenyl)-1,3-dioxolane (5)

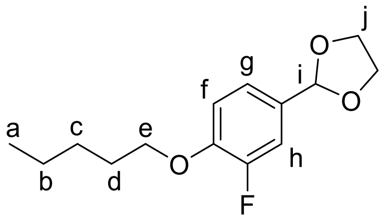

Compound **6** (0.380 g, 1.80 mmol) was dissolved in CH_2Cl_2 (10 mL) in a round-bottomed flask. The solvent was removed under reduced pressure to afford an oil. Ethylene glycol (40 mL) was added to the flask. The mixture was stirred to give a suspension, and then *p*-toluenesulfonic acid (0.270 g, 1.44 mmol) was added to the solution. The flask was heated to 80 °C in the water bath of a rotary evaporator and carefully evacuated for short periods to remove water while retaining ethylene glycol. The reaction was monitored by ^{1}H and ^{19}F NMR spectroscopy. Once the reaction had gone to completion, the solution was dissolved in CH_2Cl_2 (150 mL) and washed with saturated aqueous $NaHCO_3$ (3 × 150 mL) and brine (3 × 150 mL). The organic layer was dried over $MgSO_4$, then volatiles were removed under reduced pressure to obtain the final product **5** as a white solid (0.375 g, yield: 82%).
^{1}H NMR (400 MHz, $CDCl_3$) δ 7.21 (dd, *J* = 11.8, 2.0 Hz, 1H, g), 7.17–7.13 (m, 1H, h), 6.94 (t, *J* = 8.4 Hz, 1H, f), 5.73 (s, 1H, i), 4.16–4.07 (m, 2H, e), 4.06–3.98 (m, 4H, j), 1.86–1.77 (m, 2H, d), 1.49–1.32 (m, 4H, b,c), 0.93 (t, *J* = 7.2 Hz, 3H, a).
^{13}C NMR (101 MHz, $CDCl_3$) δ 152.6 (d, *J* = 246.3 Hz), 148.0 (d, *J* = 10.7 Hz), 130.9 (d, *J* = 5.7 Hz), 122.6 (d, *J* = 3.6 Hz), 114.4 (d, *J* = 19.6 Hz), 114.4, 103.0, 69.5, 65.3, 65.3, 28.9, 28.1, 22.5, 14.1.
^{19}F NMR (470 MHz, $CDCl_3$) δ –134.2.
HRMS (ESI$^+$) *m/z* calculated for $C_{14}H_{19}FO_3$ $[M+H]^+$ 255.1391, found 255.1384.

1-(3-Fluoro-4-(pentyloxy)phenyl)-N-octylmethanimine (S9)

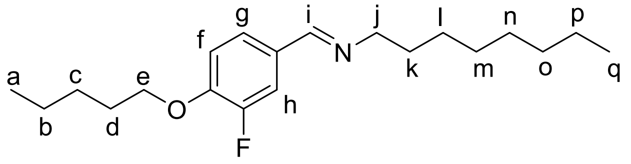

Compound **6** (0.300 g, 1.43 mmol) and octylamine (0.185 g, 1.43 mmol) were dissolved in MeOH (6 mL). The mixture was heated at 50 °C overnight. MeOH was then removed under reduced pressure and the residue obtained was re-dissolved in CH_2Cl_2 and dried over $MgSO_4$. Volatiles were then removed under reduced pressure to obtain the final product as a yellow oil (0.390 g, yield 85%).
^{1}H NMR (400 MHz, $CDCl_3$) δ 8.14 (q, *J* = 1.4 Hz, 1H, i), 7.51 (dd, *J* = 12.0, 2.0 Hz, 1H, h), 7.34 (ddd, *J* = 8.4, 2.0, 1.2 Hz, 1H, g), 6.94 (t, J = 8.3 Hz, 1H, f), 4.04 (t, *J* = 6.6 Hz, 2H, e), 3.55 (td, *J* = 7.1, 1.3 Hz, 2H, j), 1.88–1.77 (m, 2H, d), 1.71–1.61 (m, 2H, k) 1.52–1.16 (m, 14H, b, c, l, m, n, o, p), 0.92 (t, *J* = 7.1 Hz, 3H, a), 0.87 (t, *J* = 6.8 Hz, 3H, q).
^{13}C NMR (101 MHz, $CDCl_3$) δ 159.2 (d, *J* = 2.5 Hz), 152.8 (d, *J* = 246.6Hz), 149.3 (d, *J* = 10.9 Hz), 129.8 (d, *J* = 6.0 Hz), 124.9 (d, *J* = 3.3 Hz), 114.9 (d, *J* = 19.2 Hz), 114.0 (d, *J* = 1.9 Hz), 69.4, 61.7, 31.9, 31.0, 29.5, 29.4, 28.9, 28.2, 27.5, 22.8, 22.5, 14.2, 14.1.
^{19}F NMR (376 MHz, $CDCl_3$) δ –134.8.
HRMS (ESI$^+$) *m/z* calculated for $C_{20}H_{32}FNO$ $[M+H]^+$ 322.2541, found 322.2534.

1-(3-Fluoro-4-(pentyloxy)phenyl)-N-(4-fluorobenzyl)methanimine (S10)

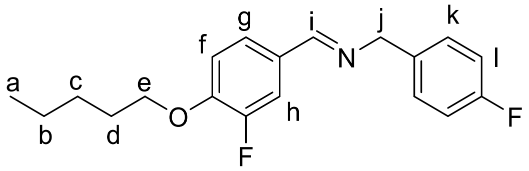

Compound **6** (0.300 g, 1.43 mmol) and 4-fluorobenzylamine (0.180 g, 1.43 mmol) were dissolved in MeOH (6 mL). The mixture was heated at 50 °C overnight. Volatiles were then removed under reduced pressure and the residue obtained was re-dissolved in CH_2Cl_2 and dried over $MgSO_4$. Volatiles were again removed under reduced pressure and the solid obtained was re-dissolved in hexane. The solution was cooled to –18 °C in a freezer, during which time a pale-yellow solid precipitated from the solution. The mixture was filtered to remove hexane and obtain the final product as a pale-yellow solid (0.170 g, yield 38%).
^{1}H NMR (400 MHz, $CDCl_3$) δ 8.26 (q, *J* = 1.5 Hz, 1H, i), 7.58 (dd, *J* = 12.0, 2.0 Hz, 1H, h), 7.40 (ddd, *J* = 8.4, 2.0, 1.2 Hz, 1H, g), 7.32–7.26 (m, 2H, k), 7.06–6.99 (m, 2H, l), 6.96 (t, J = 8.3 Hz, 1H, f), 4.74 (s, 2H, j), 4.06 (t, *J* = 6.6 Hz, 2H, e), 1.89–1.79 (m, 2H, d), 1.51–1.33 (m, 4H, b, c), 0.93 (t, *J* = 7.1 Hz, 3H, a).
^{13}C NMR (101 MHz, $CDCl_3$) δ 162.1 (d, *J* = 244.6 Hz), 160.5 (d, *J* = 2.5 Hz), 152.8 (d, *J* = 247.3 Hz), 149.7 (d, *J* = 11.1 Hz), 135.2 (d, *J* = 3.2 Hz), 129.6 (d, *J* = 8.0 Hz), 125.3 (d, *J* = 3.2 Hz), 122.6 (d, *J* = 3.6 Hz), 115.4 (d, *J* = 21.4 Hz), 115.0 (d, *J* = 19.2 Hz), 113.9 (d, *J* = 2.2 Hz), 69.4, 64.1, 28.9, 28.1, 22.5, 14.1.
^{19}F NMR (376 MHz, $CDCl_3$) δ –116.6, –134.6.
HRMS (ESI$^+$) *m/z* calculated for $C_{19}H_{21}F_2NO$ $[M+H]^+$ 318.1664, found 318.1656.

3-Fluoro-4-(2-methoxyethoxy)benzaldehyde (8)

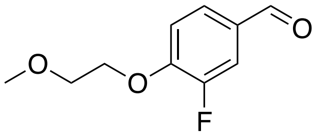

3-Fluoro-4-hydroxybenzaldehyde (1.00 g, 7.14 mmol), K_2CO_3 (2.96 g, 21.4 mmol) and KI (830 mg, 5.00 mmol) were placed in a two-necked round-bottomed flask. The system was flushed with argon and DMF (70 mL) was added to the flask. The mixture was heated to 90 °C for 1 h, then 2-bromoethylmethyl ether (740 μL, 7.85 mmol) dissolved in DMF (5 mL) was added dropwise. The reaction was heated at 90 °C for 16 h. Once the reaction was complete, volatiles were removed under reduced pressure, then the residue was re-dissolved in EtOAc (100 mL) and washed with water (1 × 100 mL) and brine (3 × 70 mL). The organic layer was dried over $MgSO_4$, filtered and evaporated under reduced pressure. The crude product was purified by flash column chromatography (SiO_2, Petroleum ether/EtOAC 2:1) to give **8** as a white solid (1.05 g, yield: 75 %).
^{1}H NMR (400 MHz, $CDCl_3$) δ 9.85 (d, *J* = 2.20 Hz, 1H), 7.64–7.58 (m, 2H), 7.10 (t, *J* = 8.20 Hz, 1H), 4.27 (t, *J* = 4.60 Hz, 2H), 3.81 (t, *J* = 4.70 Hz, 2H), 3.46 (s, 3H).
^{13}C NMR (101 MHz, $CDCl_3$) δ 190.1, 153.7–151.7 (d, *J* = 200.9), 152.5 (d, *J* = 8.80 Hz), 130.3 (d, *J* = 4.1, 128.2 (d, *J* = 2.3 Hz), 115.9 (d, *J* = 15.0 Hz), 114.0, 70.7, 69.0, 59.5.
^{19}F NMR (377 MHz, $CDCl_3$) δ –132.64.
HRMS (ESI$^+$) *m/z* calculated for $C_{10}H_{11}FNaO_3$ $[M+H]^+$ 199.0765, found 199.0766.

2-(3-fluoro-4-(2-methoxyethoxy)phenyl)-1,3-dioxolane (7)

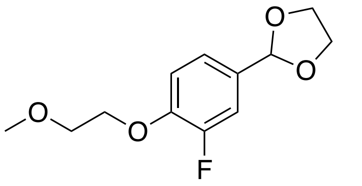

Compound **8** (1.00 g, 1.00 mmol) was dissolved in CH_2Cl_2 (10 mL) in a round-bottomed flask. The solvent was removed under reduced pressure to afford an oil. Ethylene glycol (50 mL) was added to the flask. The mixture was stirred to give a suspension, and then *p*-toluenesulfonic acid (133 mg, 700 μmol) was added to the solution. The flask was heated to 80 °C in the water bath of a rotary evaporator and carefully evacuated for short periods to remove water while retaining ethylene glycol. The reaction was monitored by ^{1}H and ^{19}F NMR spectroscopy. Once the reaction had gone to completion, the solution was dissolved in CH_2Cl_2 (150 mL) and washed with saturated aqueous $NaHCO_3$ (3 × 150 mL) and brine (3 × 150 mL). The organic layer was dried over $MgSO_4$, then volatiles were removed under reduced pressure to obtain a colourless oil. The residue was purified by flash column chromatography (SiO_2, CH_2Cl_2/triethylamine 98:2 *v/v*) to obtain the final product **7** as a white solid (0.81 g, yield: 66%).
^{1}H NMR (400 MHz, $CDCl_3$) δ 7.22 (dd, *J* = 11.72 Hz, *J* = 2.00 Hz, 1H), 7.16 (dt, *J* = 8.36 Hz, *J* = 1.48 Hz,1H), 6.98 (t, *J* = 8.28 Hz, 1H), 5.73 (s, 1 H), 4.19 (t, *J* = 4.80 Hz, 2H), 4.11 (m, 2H), 4.02 (m, 2H), 3.77 (t, *J* = 4.64, 2H), 3.45 (s, 3H).
^{13}C NMR (101 MHz, $CDCl_3$) δ 154.0–151.5 (d, *J* = 252.5 Hz), 147.7, 131.7, 122.7, 115.1, 114.7–114.5 (d, *J* = 20.2 Hz), 103.0, 71.0, 69.1, 65.4, 59.4.
^{19}F NMR (377 MHz, $CDCl_3$) δ –133.77
HRMS (ESI$^+$) *m/z* calculated for $C_{12}H_{15}FNaO_4$ $[M+H]^+$ 265.0847, found 265.0841.

3. Synthesis and characterization of gold nanoparticles

3.1 AuNP-1

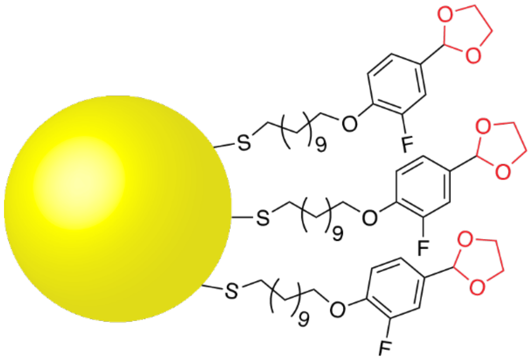

Synthesis
Disulfide $\mathbf{1}_2$ (90 mg, 0.12 mmol) and butylated hydroxytoluene (BHT, 50 mg, 0.21 mmol) were dissolved in DMF/THF (1:9 *v*/*v*, 15 mL) and the mixture was heated to 50 °C while stirring (1200 rpm). A solution of $AuPPh_3Cl$ (0.12 g, 0.24 mmol) in DMF/THF (1:9 *v*/*v*, 2 mL) was added to the reaction mixture. Borane *tert*-butylamine complex (TBAB, 210 mg, 2.40 mmol) was dissolved in DMF/THF (1:9 *v*/*v*, 1 mL) and added rapidly to the reaction mixture. Stirring was continued at 50 °C for 6 h.
The reaction was allowed to cool to room temperature, then quenched by adding Et_2O (5 mL) to achieve nanoparticle precipitation. The mixture was transferred to glass vials then centrifuged (1446 ×g rcf, 4 °C, 10 min). The supernatant was removed using a glass pipette. The black solid was subsequently washed with MeOH using the following procedure: nanoparticles were dispersed in MeOH (7 mL), sonicated for 15 min, centrifuged (1312 ×g rcf, 5 °C, 10 min) and the supernatant was removed using a glass pipette. The same operation was performed in Et_2O (7 mL) and the entire process was repeated two further times. Evaporation under reduced pressure afforded AuNP-**1** as a black solid (36.0 mg). Mean diameter (TEM, Figure S5): 5.6 ± 0.5 nm (dispersity: 9%).

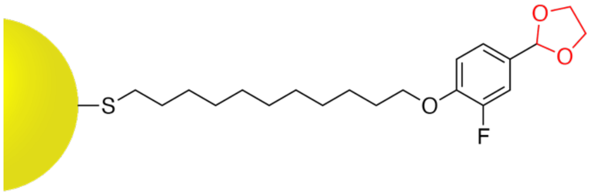

^{1}H NMR (500 MHz, $CDCl_3$): δ 6.92 (bs, 3H), 5.50 (bs, 1H), 3.87 (bs, 6H), 1.42 (bs, 18H)
^{19}F NMR (470 MHz, $CDCl_3$): δ –134.67 (s, 1F)

In situ NMR Characterization of AuNP-1

Solution-state NMR spectroscopy was employed to investigate the molecular composition of the surface-bound monolayer on AuNP-**1** (Figure S2).

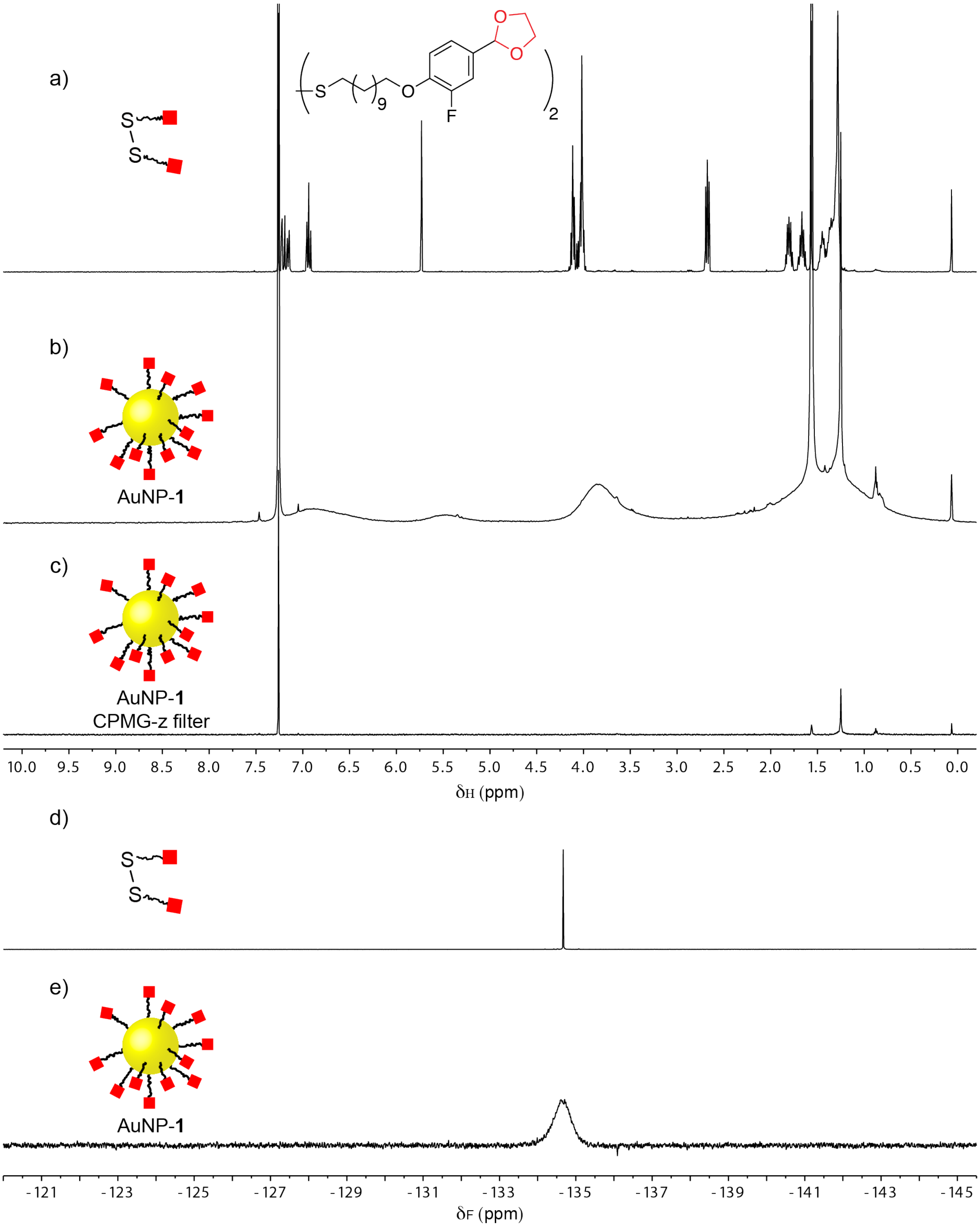

Figure S2. NMR characterization of AuNP-**1**. a) ^{1}H NMR spectrum (500 MHz, $CDCl_3$, 8 scans) of disulfide $\mathbf{1}_2$; b) ^{1}H NMR spectrum (500 MHz, $CDCl_3$, 32 scans) of AuNP-**1**. c) T_2-Filtered ^{1}H NMR spectrum (500 MHz, $CDCl_3$, 32 scans) of AuNP-**1** acquired using the CPMG-z pulse sequence.[2] All sharp peaks can be assigned to residual non-deuterated solvents. d) ^{19}F NMR (470 MHz, $CDCl_3$, 8 scans) spectrum of disulfide $\mathbf{1}_2$. e) ^{19}F NMR spectrum (470 MHz, $CDCl_3$, 32 scans) of AuNP-**1**.

Ex situ NMR Characterization of AuNP-1: oxidative ligand desorption

Ligand desorption using a mild oxidising agent allows analysis of the released molecular species in bulk solution. A colloidal solution of AuNP-**1** (2.0 mg) in $CDCl_3$ was treated with iodine (2 mg) then ^1H and ^{19}F NMR spectra were recorded immediately (Figure S3).

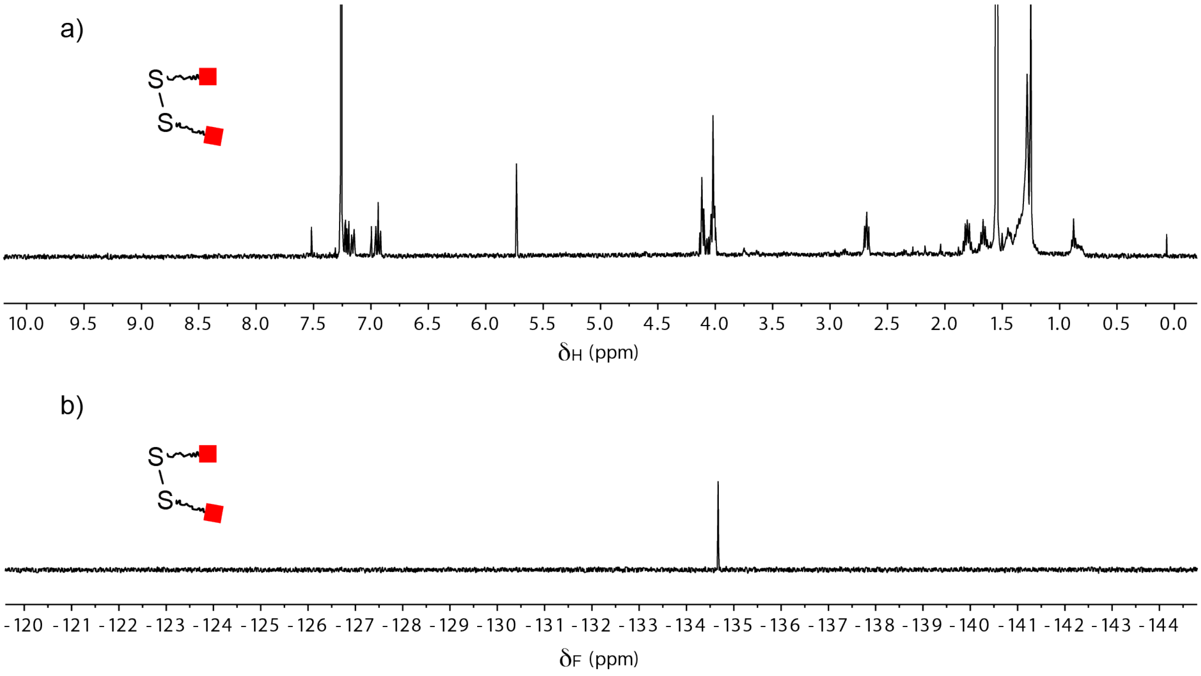

Figure S3. Oxidative ligand desorption performed on purified AuNP-**1**: a) ^1H NMR (400 MHz, $CDCl_3$); b) ^{19}F NMR (470 MHz, $CDCl_3$).

Nanoscale characterization of AuNP-1

Thermal gravimetric analysis (TGA) and differential thermal analysis (DTA) were performed by heating AuNP-**1** powder, under a stream of air (24 mL min^{-1}), 20–900 °C with a 5 min isotherm at 50 °C. A blank was recorded under the same conditions and the mass subtracted to eliminate the buoyancy effect. Thermal decomposition of the surface-bound monolayer resulted in progressive mass loss as temperature increased above the onset temperature (T_m) at 325 °C. Assuming an isotropic gold core (d = 5.6 nm, Figure S5) the organic mass lost between 50–900 °C was used to estimate molar weight and number of ligands per nanoparticle (Figure S4 and Table S1).

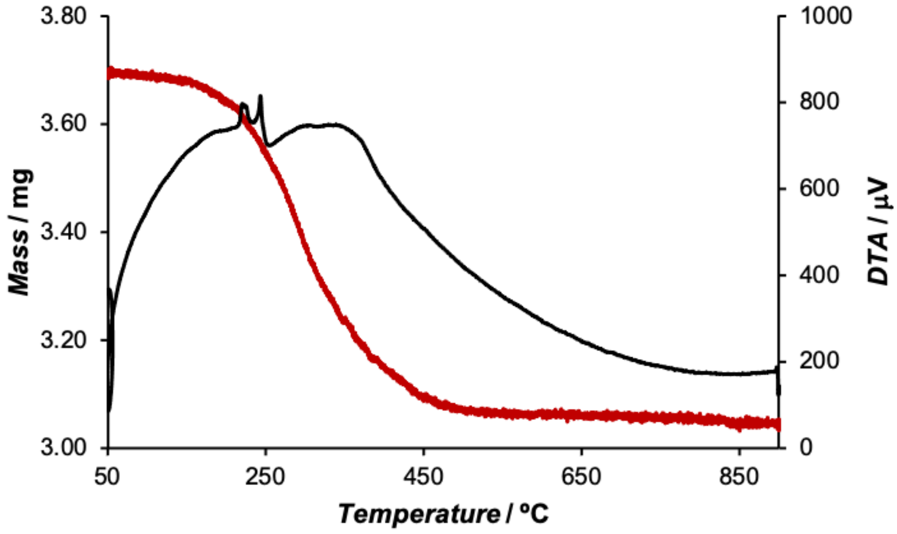

Figure S4. TGA (red curve) and DTA (black curve) plots for AuNP-**1** (3.7 mg). The sample was heated under air at a ramp rate of 5 °C min^{-1} over the range 50–900 °C.

Table S1. AuNP-**1** nanoscale characterisation calculated from TGA, DTA and TEM data.

<*d*> / nm	AuNP-**1** surface area / nm^2	Au % wt	Surface-bound **1** %	Ligand **1** surface area /nm^2	AuNP-**1** M.W. / g mol^{-1}	Molecules **1** per nanoparticle
5.60	98.5	82.2	17.8	0.157	1.30 x 10^6	628

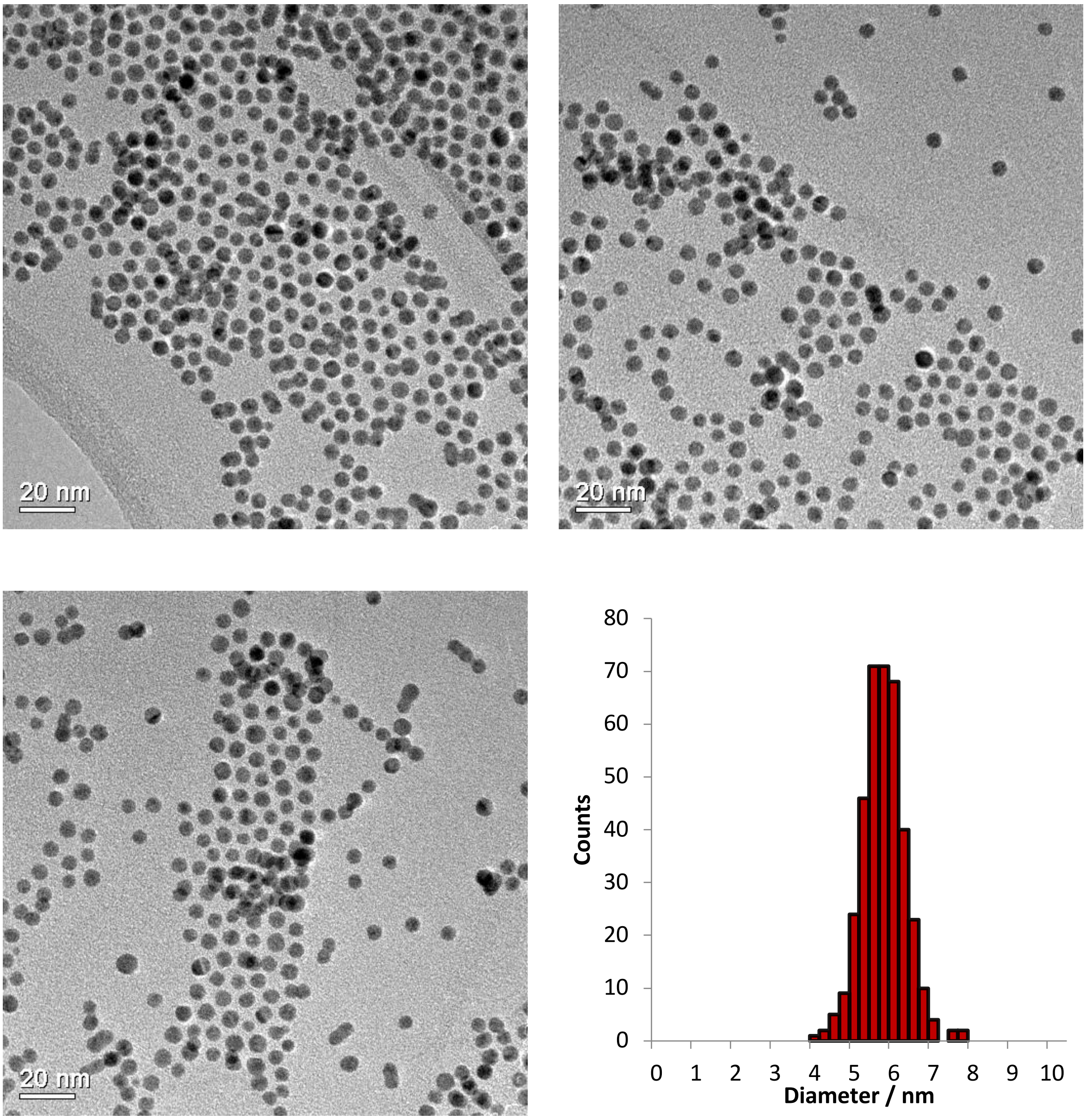

Figure S5. Representative TEM images and histogram of size distribution as found through analysis of multiple images for AuNP-**1** (<*d*> = 5.6 ± 0.5 nm).

3.2 AuNP-2

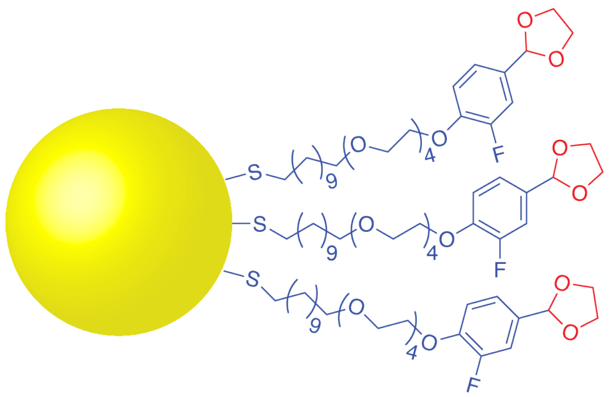

Synthesis

Disulfide **2**$_2$ (66 mg, 61 μmol) and butylated hydroxytoluene (BHT, 21 mg, 10 μmol) were dissolved in distilled DMF/THF (1:9 *v*/*v*, 4 mL) and the mixture was heated to 50 °C while stirring (800 rpm). A solution of $AuPPh_3Cl$ (50 mg, 10 μmol) in distilled DMF/THF (1:9 *v*/*v*, 2 mL) was added to the reaction mixture. Borane *tert*-butylamine complex (TBAB, 88 mg, 1.0 mmol) was dissolved in distilled DMF/THF (1:9 *v*/*v*, 1 mL) and added rapidly to the reaction mixture. Stirring was continued at 50 °C for 6 h.

The reaction was allowed to cool to room temperature, then quenched by adding Et_2O (5 mL) to achieve nanoparticle precipitation. The mixture was transferred to glass vials then centrifuged (1446 ×g rcf, 4 °C, 10 min). The supernatant was removed using a glass pipette. The black solid was subsequently washed with MeOH using the following procedure: nanoparticles were dispersed in MeOH (7 mL), sonicated for 15 min, centrifuged (1312 ×g rcf, 5 °C, 10 min) and the supernatant was removed using a glass pipette. The same operation was performed in Et_2O (7 mL) and the entire process was repeated two further times. Evaporation under reduced pressure afforded AuNP-**2** as a black solid (25.8 mg). Mean diameter (TEM, Figure S9): 4.9 ± 0.6 nm (dispersity: 12 %).

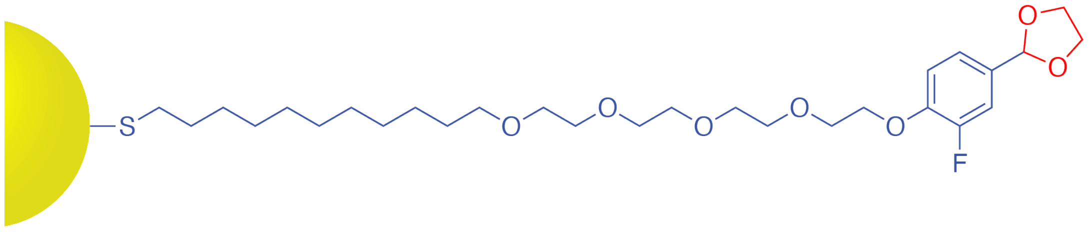

^{1}H NMR (500 MHz, $CDCl_3$): δ 7.08 (bs, 3H), 5.65 (bs, 1H), 3.76 (bs, 22H), 1.42 (bs, 18H)
^{19}F NMR (470 MHz, $CDCl_3$): δ –133.95 (s, 1F)

In situ NMR Characterization of AuNP-2

Solution-state NMR spectroscopy was employed to investigate the molecular composition of the surface-bound monolayer on AuNP-**2** (Figure S6).

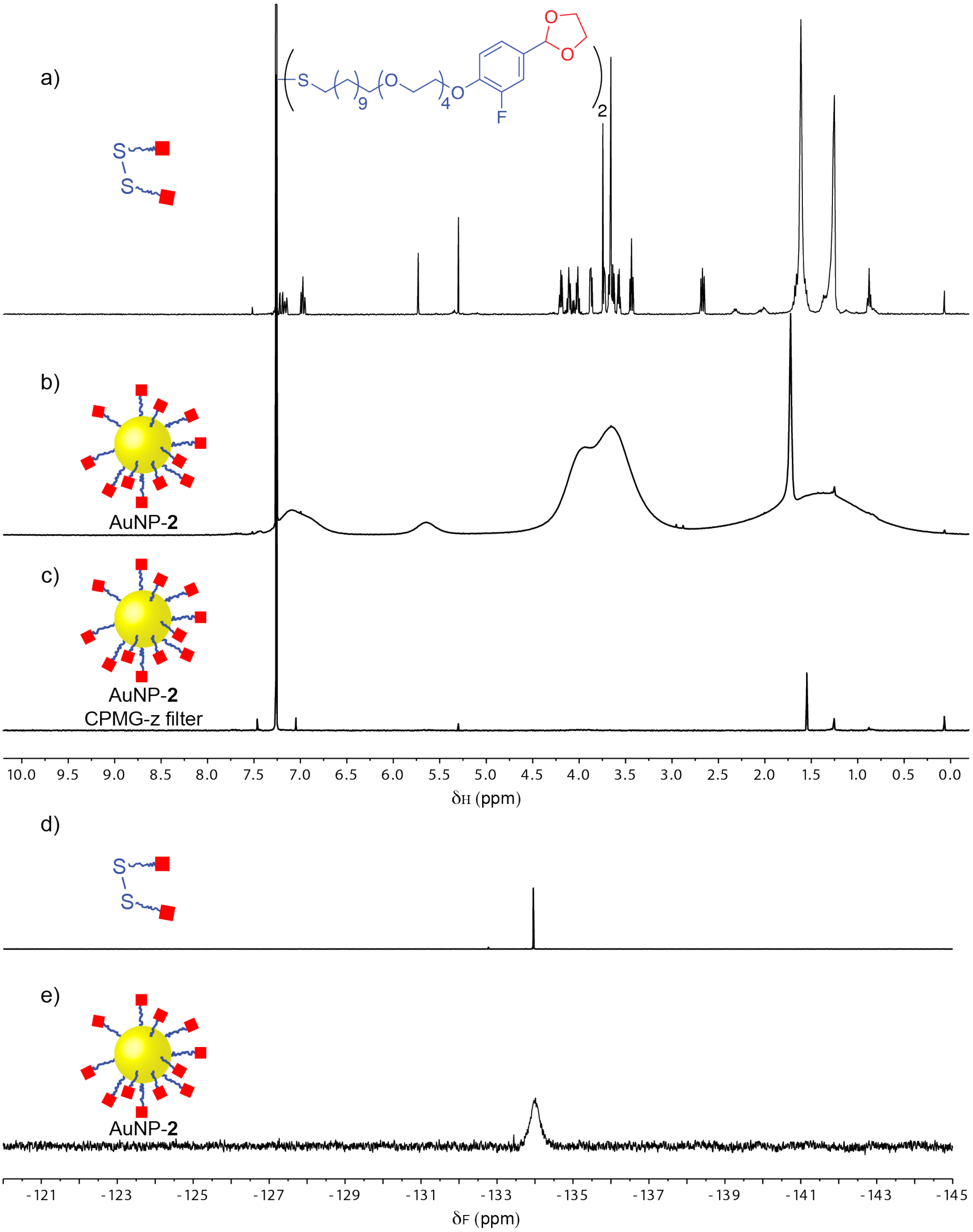

Figure S6. NMR characterization of AuNP-**2**. a) ^{1}H NMR spectrum (500 MHz, $CDCl_3$, 8 scans) of disulfide **2**$_2$; b) ^{1}H NMR spectrum (500 MHz, $CDCl_3$, 32 scans) of AuNP-**2**. c) T_2-Filtered ^{1}H NMR spectrum (500 MHz, $CDCl_3$, 32 scans) of AuNP-**2** acquired using the CPMG-z pulse sequence.[2] All sharp peaks can be assigned to residual non-deuterated solvents and impurities. d) ^{19}F NMR (470 MHz, $CDCl_3$, 8 scans) spectrum of disulfide **2**$_2$. e) ^{19}F NMR spectrum (470 MHz, $CDCl_3$, 32 scans) of AuNP-**2**.

Ex situ NMR Characterization of AuNP-2: oxidative ligand desorption

Ligand desorption using a mild oxidising agent allows analysis of the released molecular species in bulk solution. A colloidal solution of AuNP-**2** (5.6 mg) in $CDCl_3$ was treated with iodine (2 mg), then ^1H and ^{19}F spectra were recorded immediately (Figure S7).

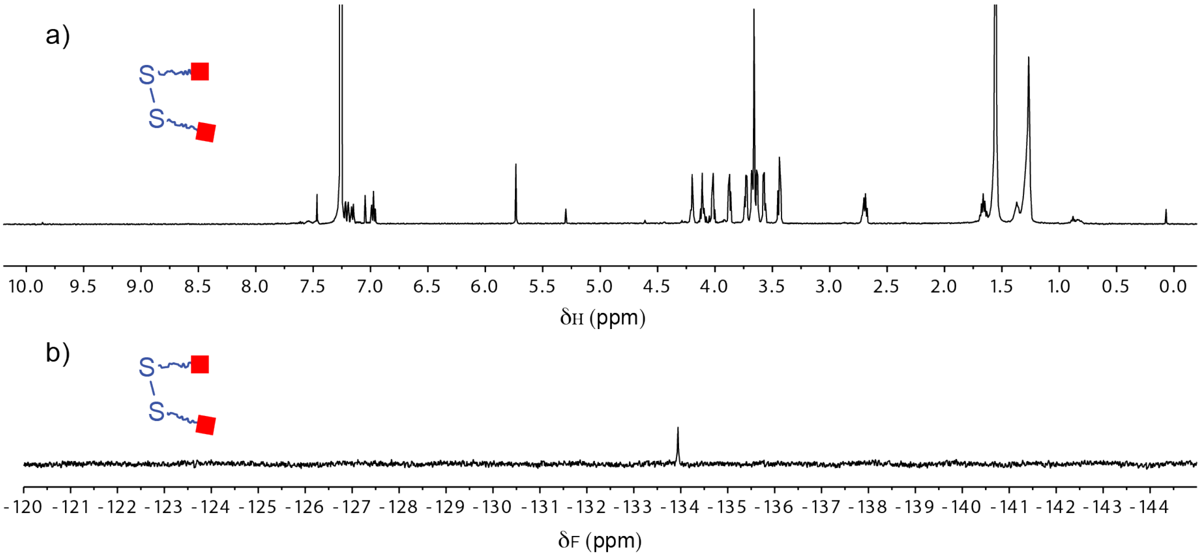

Figure S7. Oxidative ligand desorption performed on a solution of purified AuNP-**2**: a) ^1H NMR (500 MHz, $CDCl_3$); b) ^{19}F NMR (470 MHz, $CDCl_3$).

Nanoscale characterization of AuNP-2

Thermal gravimetric analysis (TGA) and differential thermal analysis (DTA) were performed by heating AuNP-**2** powder, under a stream of air (24 mL min^{-1}), over the range 20–900 °C with a 10 min isotherm at 50 ºC. A blank was recorded under the same conditions and the mass subtracted to eliminate the buoyancy effect. Thermal decomposition of AuNP-**2** surface-bound monolayer resulted in progressive mass loss as temperature increased above the onset temperature (T_m) at 315 °C. Assuming an isotropic gold core (d = 4.9 nm, Figure S9) the organic mass lost between 50–900 °C was used to estimate molar weight and number of ligands per nanoparticle (Figure S8 and Table S2).

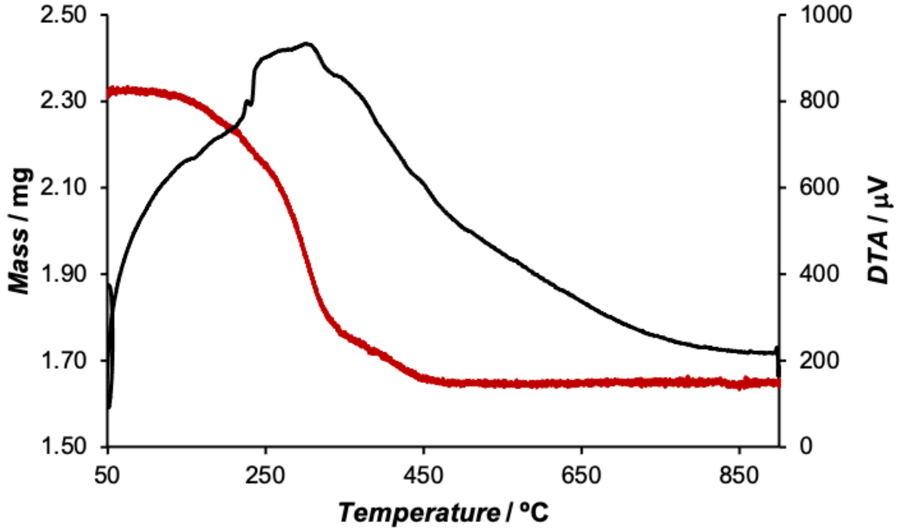

Figure S8. TGA (red curve) and DTA (black curve) plots for AuNP-**2** (2.3 mg). The sample was heated under air at a ramp rate of 5 °C min^{-1} over the range 50–900 °C.

Table S2. AuNP-**2** nanoscale characterisation calculated from TGA, DTA and TEM data.

<*d*> / nm	AuNP-**2** surface area / nm^2	Au % wt	Surface-bound **2** %	Ligand **2** surface area /nm^2	AuNP-**2** M.W. / g mol^{-1}	Molecules **2** per nanoparticle
4.90	75.4	71.7	28.3	0.146	9.98×10^5	517

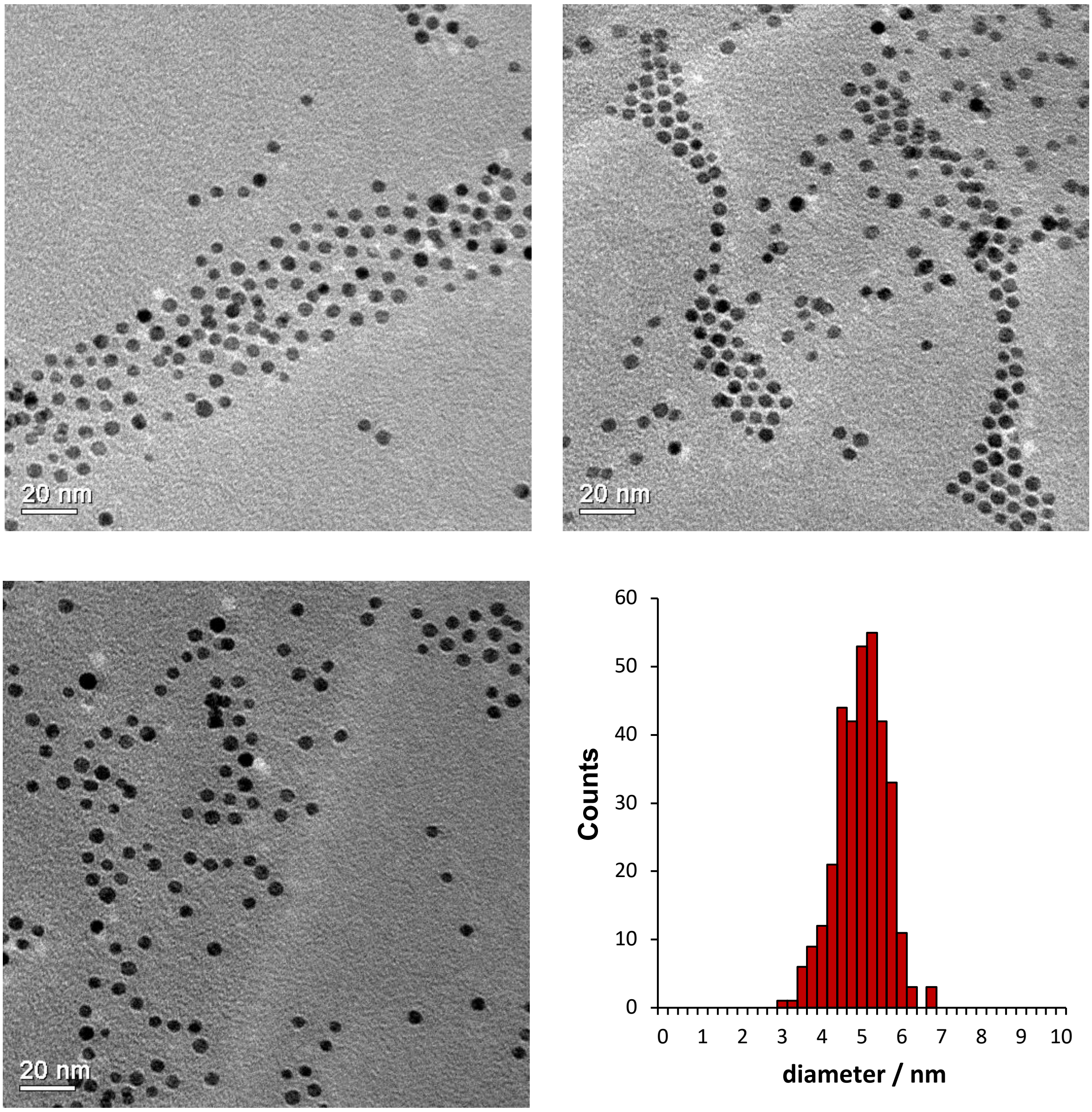

Figure S9. Representative TEM images and histogram of size distribution as found through analysis of multiple images for AuNP-**2** (<*d*> = 4.9 ± 0.6 nm).

4. Identification and elimination of monolayer impurities

Butylated hydroxytoluene (BHT), a radical scavenger, was added to avoid cluster-catalyzed oxidation of acetals by reactive oxygen species that were either endogenous to the THF solvent or generated during the synthetic procedure.[3]

Nevertheless, when characterizing an early batch of AuNP-**1** by ^{19}F NMR spectroscopy (Figure S10), oxidative ligand desorption (procedure as described in Section 3.1) revealed a signal at –134.55 ppm (blue arrow, Figure S10b), which corresponds to neither the acetal disulfide **1**$_2$ nor the analogous aldehyde **S4**.

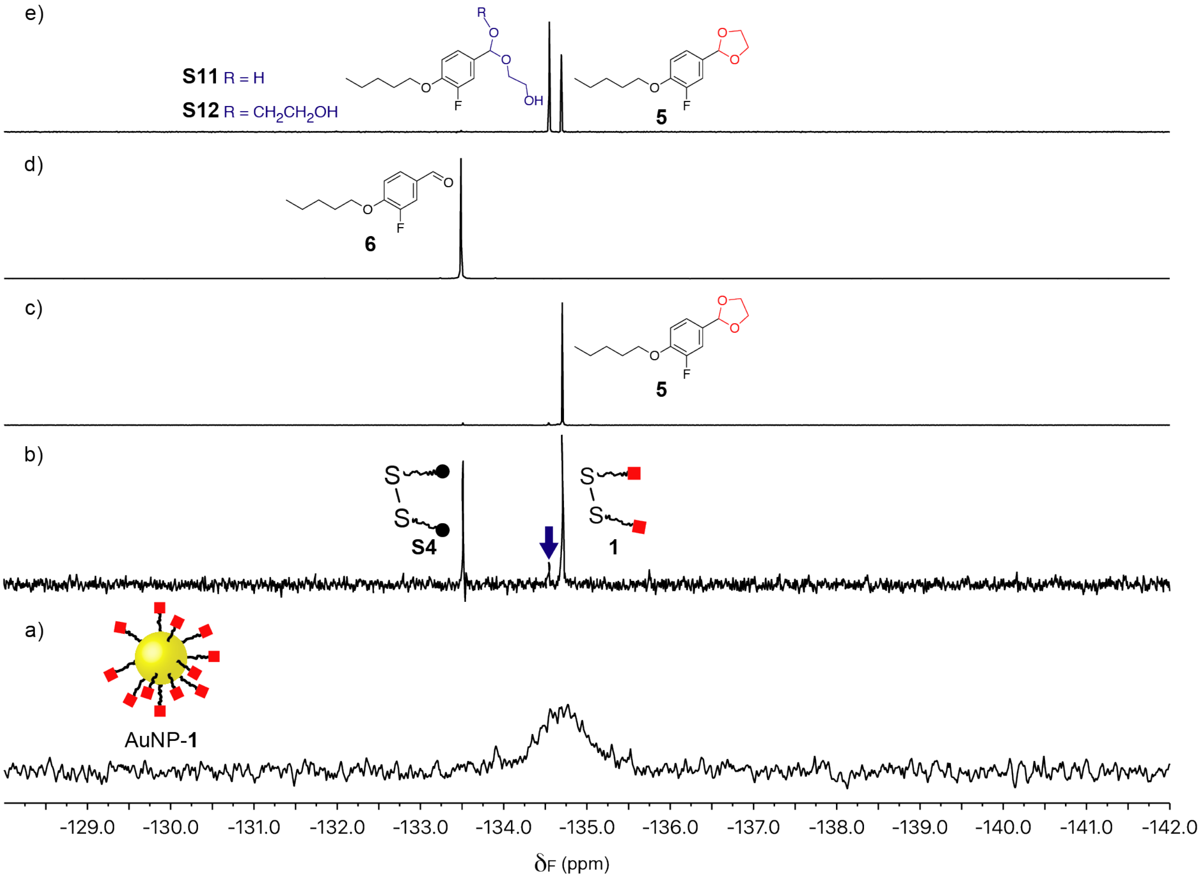

Figure S10. ^{19}F NMR spectra (470 MHz, $CDCl_3$) of: a) AuNP-**1**; b) oxidative ligand desorption performed on a solution of purified AuNP-**1**; c) acetal model compound **5**; d) aldehyde model compound **6**; e) mixture of model acyclic acetal **S11** or **S12** (–134.55 ppm) and cyclic acetal **5** (–134.68 ppm).

In order to identify the source of this unknown peak at –134.55 ppm, acetal formation was studied using model compound **6**. *p*-Toluenesulfonic acid (47 mg, 0.25 mmol) was added to a solution of compound **6** (75 mg, 0.32 mmol) in ethylene glycol (18 mL). The reaction mixture was heated to 50 °C under reduced pressure for 3 h. The reaction was cooled to room temperature and extracted with CH_2Cl_2 (3 × 50 mL) containing Et_3N (1% *v*/*v*). The organics were washed with a saturated solution of $NaHCO_3$ (2 × 40 mL) then dried over $MgSO_4$. Volatiles were removed under reduced pressure to obtain a pale-yellow oil (57 mg).
Analysis by ^{19}F NMR spectroscopy (Figure S10e) revealed two signals, corresponding to the expected acetal model compound **5** (at –134.68 ppm) and an unknown compound at the chemical shift (–134.55 ppm) observed for the impurity in the nanoparticle experiment (Figure 10b).
Although isolation of a pure sample of the unknown compound was unsuccessful, further characterization of the mixture obtained from the model compound experient by ^1H NMR

spectroscopy (Figure S11c) identified the impurity as an acyclic acetal compound, hemi-acetal **S11** or bis-ethan-1-ol acetal **S12**.

This assignment was reinforced by incubation of the mixture with excess of CF_3CO_2H at 25 °C, which resulted in complete disappereance of the resonances for the unknown species after 2 h, with the only new peaks corresponding to the aldehyde model compound **6** and traces of cyclic acetal model compound **5** (Figure S11d).

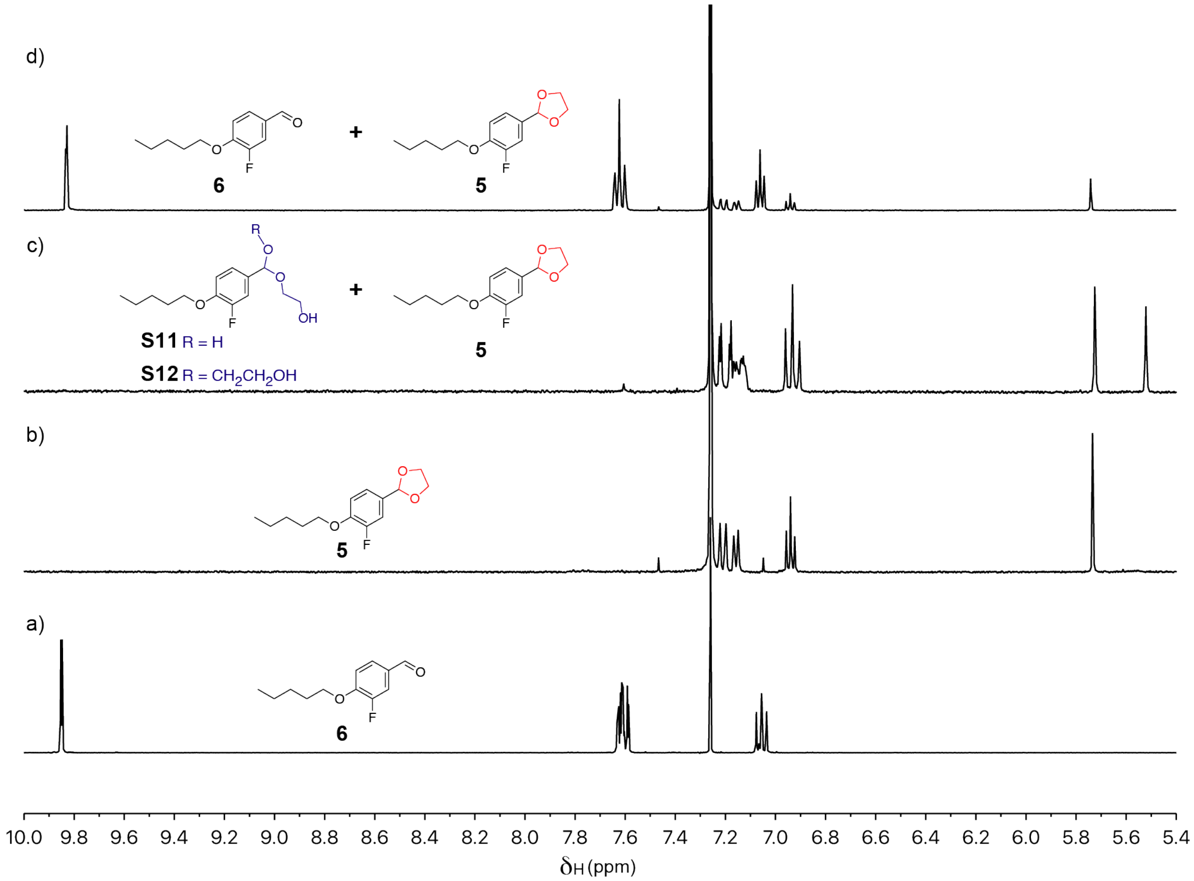

Figure S11. ^{1}H NMR spectra (500 MHz, $CDCl_3$) of: a) aldehyde model compound **6**; b) cyclic acetal model compound **5**; c) mixture of model acyclic acetal **S11** or **S12** and cyclic acetal **5**; d) mixture of cyclic acetal model compound **5** and aldehyde model compound **6** after 2 h incubation of mixture c with CF_3CO_2H at 25 °C.

To avoid formation of the acyclic acetal impurity, it is crucial to limit the duration of the acetal formation procedure to less than 2 h and to maintain a reaction temperature above 80 °C. Following this procedure carefully, eliminated the acyclic acetal impurity from all further batches of acetal functionalized AuNPs.

5. Exhaustive and partial on-nanoparticle acetal hydrolysis

A stock solution of CF_3CO_2H was freshly prepared for each experiment in the reaction solvent mixture with 4-fluorotoluene as internal standard (5.00 mM) and concentration measured by ^{19}F NMR.

5.1 Exhaustive acetal hydrolysis from AuNP-1

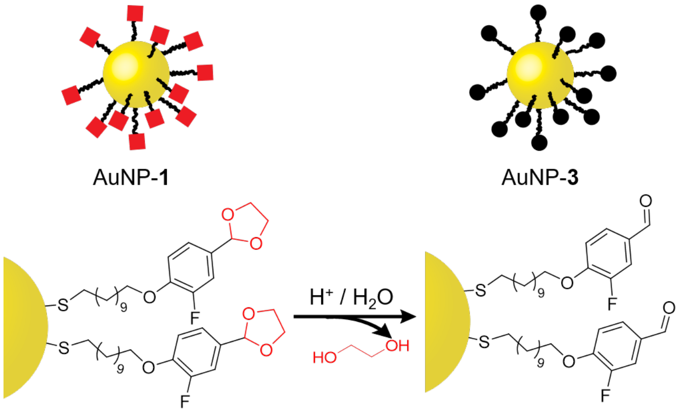

A colloidal solution of AuNP-**1** containing 4-fluorotoluene (5.00 mM) as internal standard was prepared in distilled DMF/D_2O (97:3 *v*/*v*, 600 μL) giving 5.06 mM in terms of surface-bound acetal **1**. This solution was warmed to 50 °C, then an aliquot of the CF_3CO_2H stock solution (11 μL) added, giving a final concentration of CF_3CO_2H of 20.3 mM. This mixture was incubated at 50 °C and the reaction was followed by ^{19}F NMR spectroscopy. A new broad nanoparticle-bound signal appeared at –133.06 ppm, corresponding to nanoparticle-bound aldehyde **3**, which grew in intensity concomitant with disappearance of the signal for nanoparticle-bound acetal **1** (Figure S12). No further changes were observed after 7 h.
Nanoparticles were precipitated by adding Et_2O/EtOH (8:1 *v*/*v*, 10 mL). The black solid recovered was resuspended in Et_2O/EtOH (7:1 *v*/*v*, 8 mL), sonicated for 10 min and recollected by centrifugation (1312 ×g rcf, 4 °C, 20 min). This operation was repeated a further twice. Traces of volatile solvents were removed from the purified residue under a stream of compressed air.

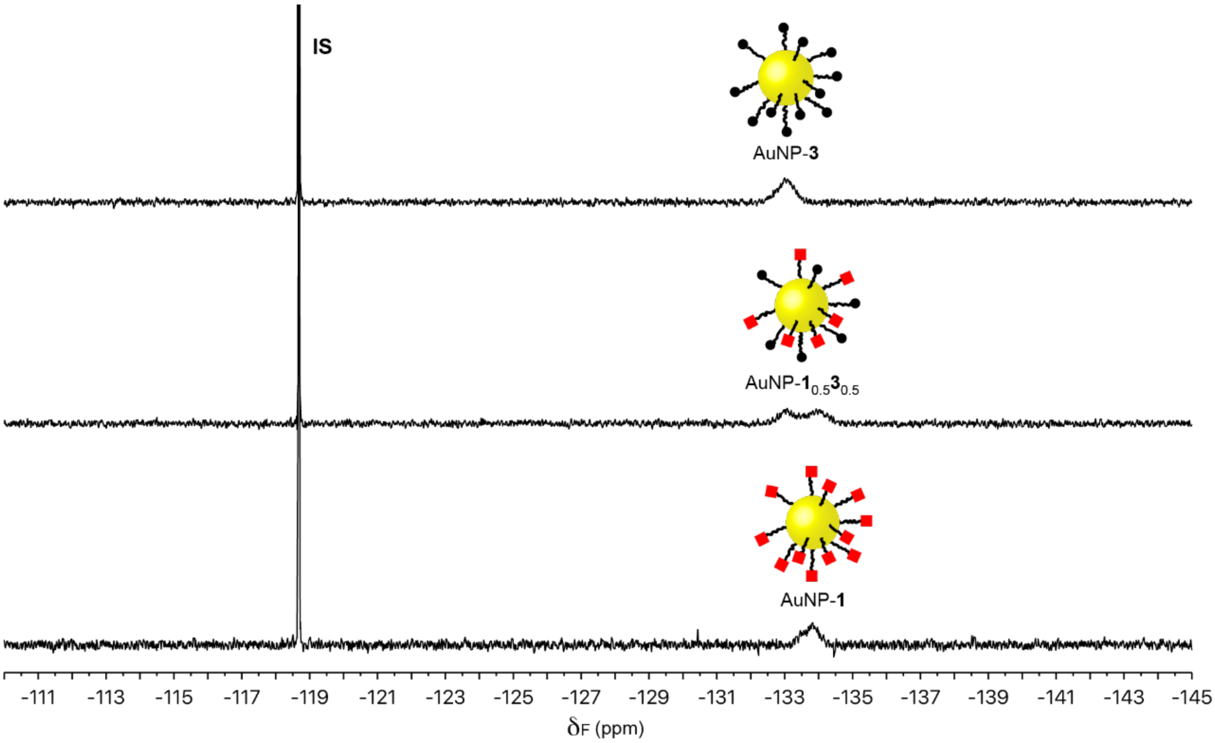

Figure S12. In situ monitoring of acetal hydrolysis from AuNP-**1** to AuNP-**3** by ^{19}F NMR spectroscopy (470 MHz, DMF/D_2O 97:3 *v*/*v*, d1 = 25 s). Bottom: AuNP-**1** (5.06 mM); middle: reaction mixture incubated at 50 °C for 40 min after addition of CF_3CO_2H (22.3 mM), indicating a 1:1 ratio of **1** and **3** surface-bound ligands; top: AuNP-**3** (4.97 mM). IS: internal standard (4-fluorotoluene, 5.00 mM).

5.2 Exhaustive acetal hydrolysis from AuNP-2

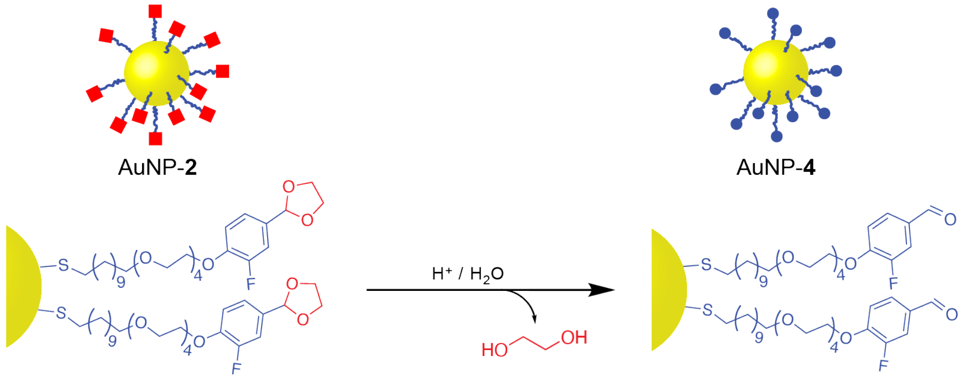

A colloidal solution of AuNP-**2** containing 4-fluorotoluene (5.00 mM) as internal standard was prepared in distilled DMF/D_2O (97:3 *v*/*v*, 600 μL) giving 5.14 mM in terms of surface-bound acetal **2**. This solution was heated to 50 °C, then an aliquot of the CF_3CO_2H stock solution (1.062 M, 11.5 μL) added, giving final concentrations of AuNP-**2** (5.05 mM) and CF_3CO_2H (20.1 mM). This mixture was incubated at 50 °C and the reaction was followed by ^{19}F NMR spectroscopy. A new broad nanoparticle-bound signal appeared at –133.04 ppm, corresponding to nanoparticle-bound aldehyde **4** (Figure S13). No further changes were observed after 6 h.

Nanoparticles were precipitated by adding Et_2O/EtOH (8:1 *v*/*v*, 10 mL). The black solid recovered was resuspended in Et_2O/EtOH (7:1 *v*/*v*, 8 mL), sonicated for 10 min and recollected by centrifugation (1312 ×g rcf, 4 °C, 20 min). This operation was repeated a further twice. Traces of volatile solvents were removed from the purified residue under a stream of compressed air.

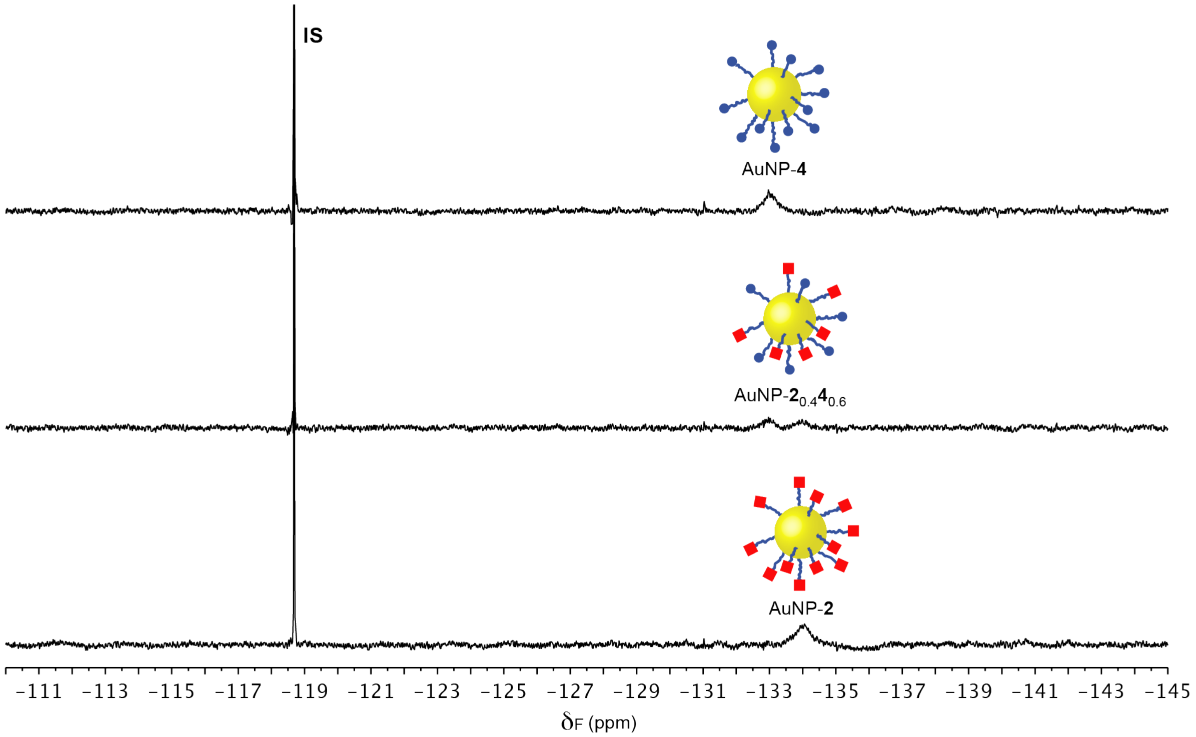

Figure S13. In situ monitoring of acetal hydrolysis from AuNP-**2** to AuNP-**4** by ^{19}F NMR spectroscopy (470 MHz, DMF/D_2O 97:3 *v*/*v*, d1 = 25 s). Bottom: AuNP-**2** (5.05 mM); middle: reaction mixture incubated at 50 °C for 40 min after addition of CF_3CO_2H (20.1 mM), indicating a 0.4:0.6 ratio of **2** and **4** surface-bound ligands. Top: AuNP-**4** (5.05 mM). IS: internal standard (4-fluorotoluene, 5.00 mM).

5.3 Partial acetal hydrolysis from AuNP-1

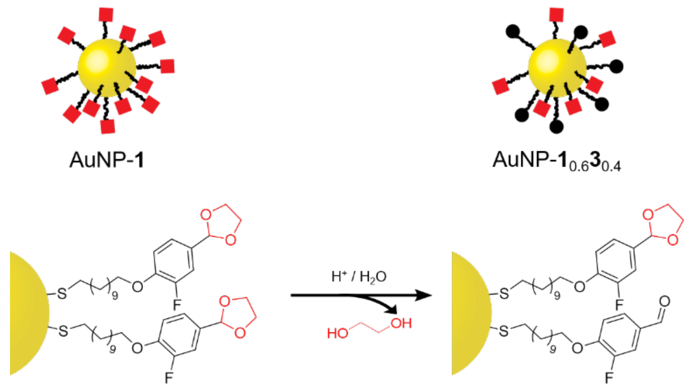

A colloidal solution of AuNP-**1** containing 4-fluorotoluene as internal standard (5.00 mM) was prepared in THF/D_2O (9:1 *v*/*v*, 600 μL) giving 5.90 mM in terms of surface-bound acetal **1**. This solution was heated to 50 °C, then an aliquot of the CF_3CO_2H stock solution (13.5 μL) was added, giving a final concentration of AuNP-**1** (5.77 mM) and CF_3CO_2H (23.2 mM). This mixture was incubated at 50 °C and the reaction was followed by ^{19}F NMR spectroscopy. A new broad nanoparticle-bound signal appeared at –133.07 ppm, corresponding to nanoparticle-bound aldehyde **3** (Figure S14). After 2 h, the solution was removed from heating, and triethylamine (2.4 μL) was added to neutralise CF_3CO_2H.

Nanoparticles were precipitated by adding Et_2O/MeOH (4:1 *v*/*v*, 10 mL). The black solid recovered was resuspended in Et_2O/MeOH (4:1 *v*/*v*, 15 mL), sonicated for 10 min, then recollected by centrifugation (1446 ×g rcf, 4 °C, 10 min). This operation was repeated a further twice. Traces of volatile solvents were removed from the purified residue under a stream of compressed air.

The composition of the nanoparticle-bound monolayer was determined by deconvolution of the relevant signals in the in situ ^{19}F NMR spectrum (Figure S14), revealing 60% acetal and 40% aldehyde ligands (AuNP-$\mathbf{1}_{0.6}\mathbf{3}_{0.4}$).

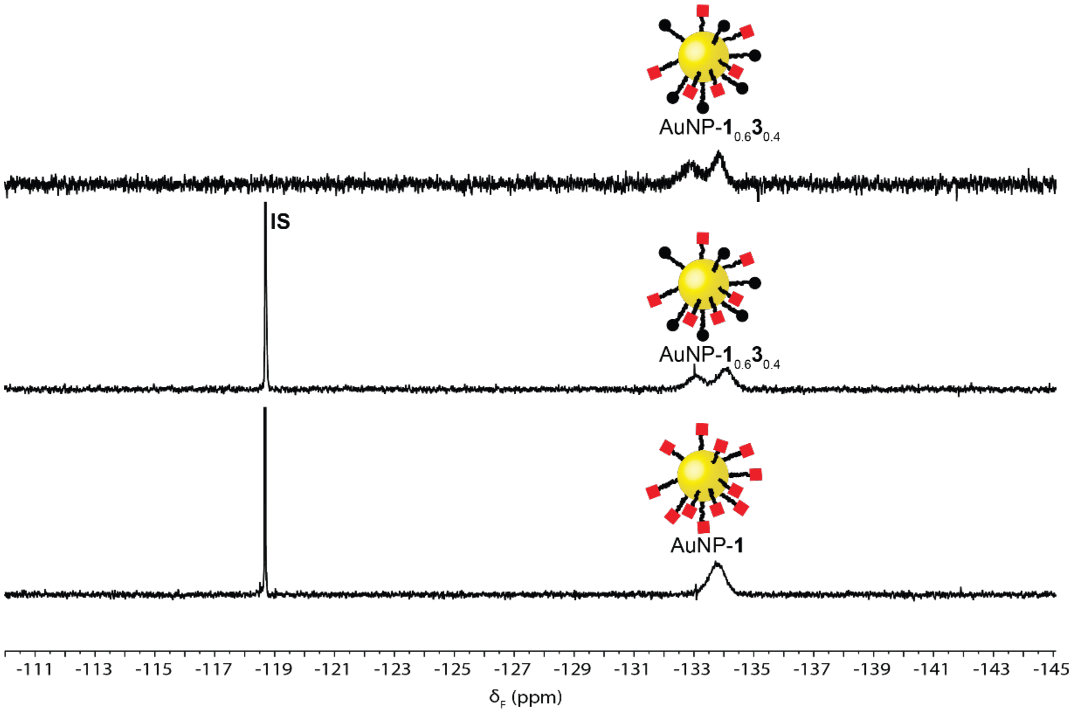

Figure S14. In situ monitoring of acetal deprotection from AuNP-**1** to AuNP-$\mathbf{1}_{0.6}\mathbf{3}_{0.4}$ by ^{19}F NMR spectroscopy (470 MHz, THF/D_2O 9:1 *v*/*v*, d1 = 25 s). Bottom: AuNP-**1** (5.90 mM); middle: Reaction mixture incubated at 50 °C for 2 h after addition of CF_3CO_2H (22.3 mM), indicating a 0.6:0.4 ratio of **1** and **3** surface-bound functionalities; top: Purified AuNP-$\mathbf{1}_{0.6}\mathbf{3}_{0.4}$. IS: internal standard (4-fluorotoluene, 5.00 mM).

5.4 Partial acetal hydrolysis from AuNP-2

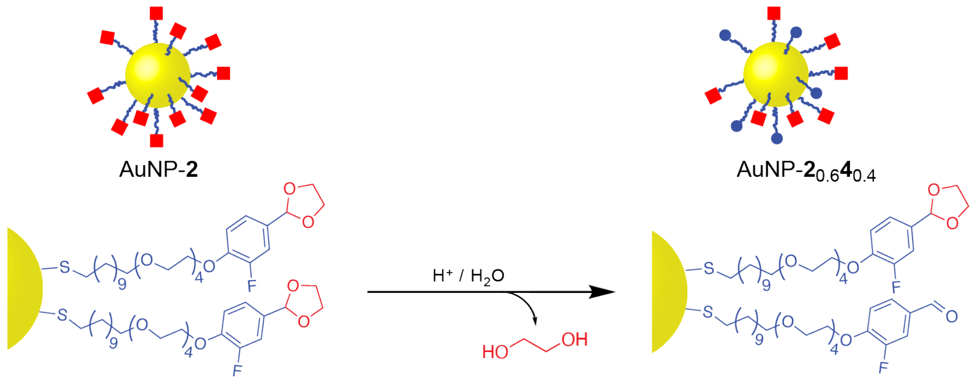

A stock solution of CF_3CO_2H was freshly prepared in distilled DMF/D_2O 9:1 *v*/*v* with 4-fluorotoluene as internal standard (5.00 mM) and concentration measured by ^{19}F NMR
A colloidal solution of AuNP-**2** containing 4-fluorotoluene (5.00 mM) was prepared in distilled DMF/D_2O (9:1 *v*/*v*, 600 µL) giving 7.33 mM in terms of surface-bound acetal **2**. Then, an aliquot of the CF_3CO_2H stock solution (1.022 M, 11.7 µL) was added, giving a final concentration of AuNP-**2** (7.26 mM) and CF_3CO_2H (20.1 mM).
This mixture was incubated at room temperature and the reaction was followed by ^{19}F NMR spectroscopy. A new broad nanoparticle-bound signal appeared at –132.95 ppm, corresponding to nanoparticle-bound aldehyde **4** (Figure S15). After 100 min, the nanoparticles were precipitated by adding Et_2O/MeOH (4:1 *v*/*v*, 10 mL) to stop the hydrolysis reaction. The black solid was recovered by centrifugation (1446 ×g rcf, 4 °C, 10 min), then resuspended in Et_2O/MeOH (4:1 *v*/*v*, 15 mL), sonicated for 10 min and recollected by

centrifugation. This operation was repeated further twice. Traces of volatile solvents were removed from the purified residue under a stream of compressed air.
The composition of the nanoparticle-bound monolayer was determined by deconvolution of the relevant signals in the in situ ^{19}F NMR spectrum (Figure S15), revealing 60% acetal and 40% aldehyde ligands (AuNP-$\mathbf{2}_{0.6}\mathbf{4}_{0.4}$).

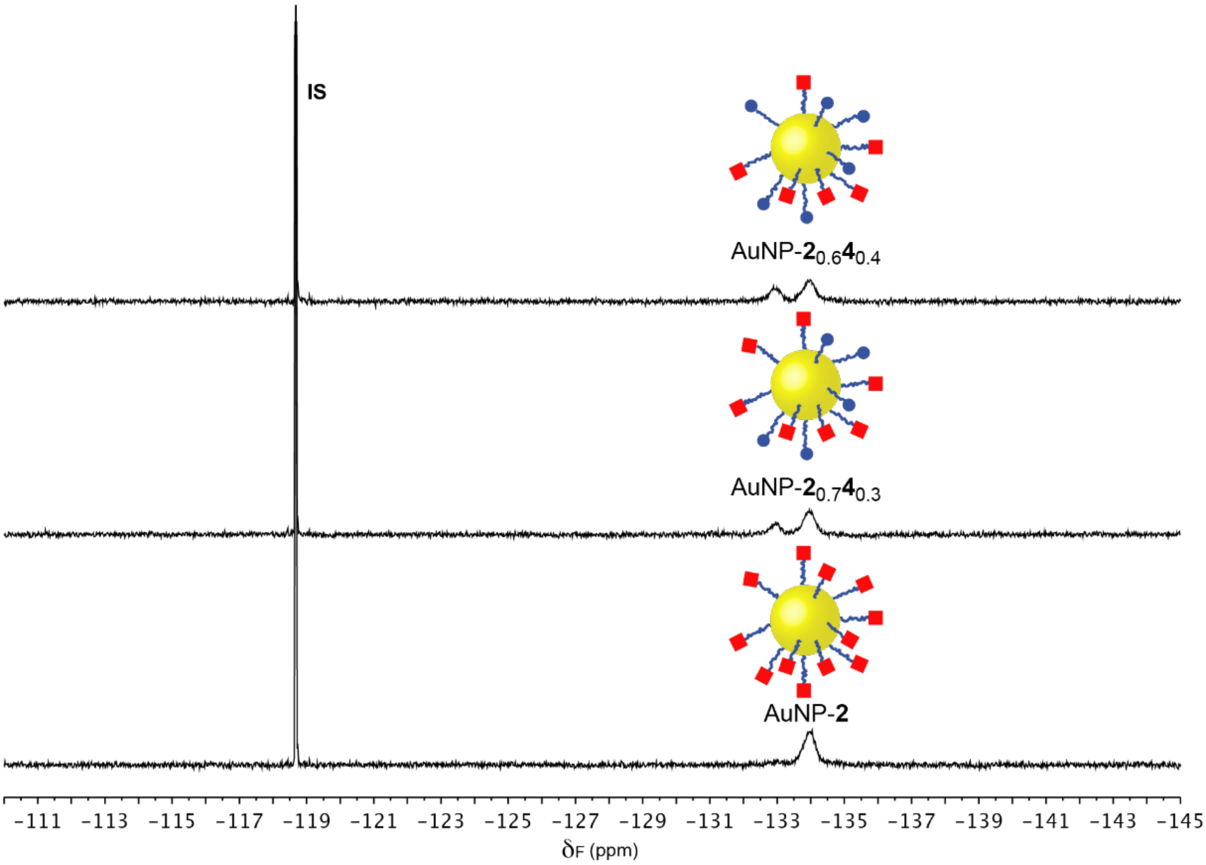

Figure S15. In situ monitoring of acetal deprotection from AuNP-**2** to AuNP-$\mathbf{2}_{0.6}\mathbf{4}_{0.4}$ by ^{19}F NMR spectroscopy (470 MHz, DMF/D_2O 9:1 *v*/*v*, d1 = 25 s). Bottom: AuNP-**2** (7.26 mM); middle: Reaction mixture incubated at room temperature for 60 min after addition of CF_3CO_2H (20.1 mM), indicating a 0.7:0.3 ratio of **2** and **4** surface-bound ligands; top: Reaction mixture incubated at room temperature for 100 min after addition of CF_3CO_2H (20.1 mM), indicating a 0.6:0.4 ratio of **2** and **4** surface-bound ligands IS: internal standard (4-fluorotoluene, 5.00 mM).

6. Hydrolysis of acetal model compounds

A stock solution of CF_3CO_2H was freshly prepared for each experiment in distilled DMF/D_2O 97:3 *v/v* with 4-fluorotoluene as internal standard (5.00 mM) and concentration measured by ^{19}F NMR

6.1 Hydrolysis of acetal model compound 5

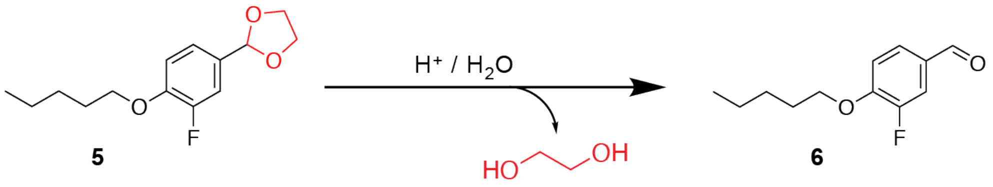

A solution of compound **5** containing 4-fluorotoluene (5.00 mM) as internal standard was prepared in freshly distilled DMF/D_2O (97:3 *v/v*, 600 µL). This mixture was heated to 50 °C, then an aliquot of the CF_3CO_2H stock solution (1.059 M, 11.5 µL) added, giving final concentrations of **5** (4.86 mM) and CF_3CO_2H (20.4 mM). This mixture was incubated at 50 °C and the reaction was followed by ^{19}F NMR spectroscopy. A new signal appeared at –133.97 ppm, corresponding to aldehyde **6** (Figure S16). No further changes were observed after 1.5 h.

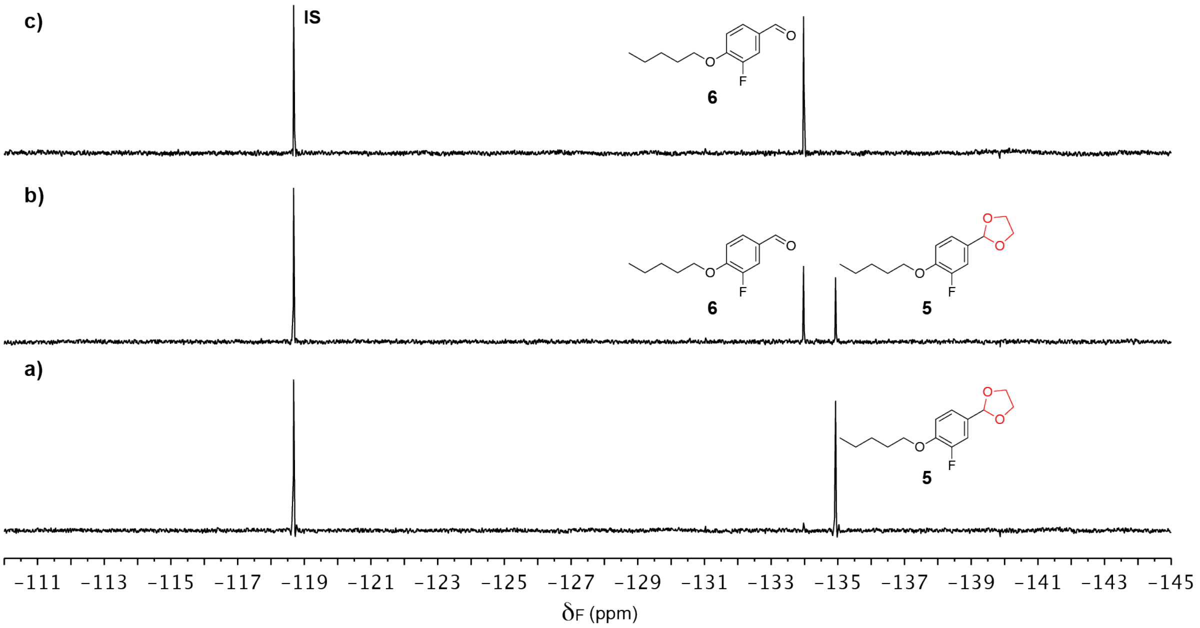

Figure S16. In situ monitoring of acetal hydrolysis from compound **5** to compound **6** by ^{19}F NMR spectroscopy (470 MHz, DMF/D_2O 97:3 *v/v*, d1 = 25 s). a) Compound **5** (4.86 mM); b) reaction mixture incubated at 50 °C for 10 min after addition of CF_3CO_2H (20.4 mM), indicating a 1:1 ratio of **5** and **6**; c) compound **6** (4.86 mM). IS: internal standard (4-fluorotoluene, 5.00 mM).

6.2 Hydrolysis of acetal model compound 7

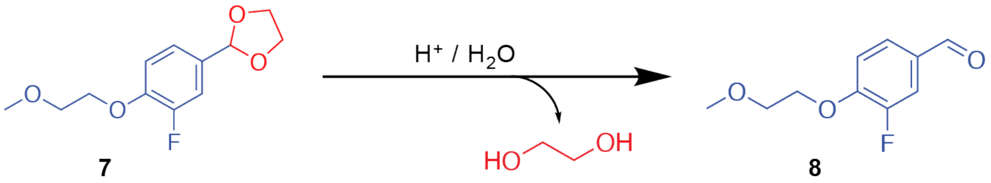

A solution of compound **7** containing 4-fluorotoluene (5.00 mM) as internal standard was prepared in freshly distilled DMF/D_2O (97:3 *v*/*v*, 600 µL). This mixture was heated to 50 °C, then an aliquot of the CF_3CO_2H stock solution (1.020 M, 12.5 µL) added, giving final concentrations of **7** (5.50 mM) and CF_3CO_2H (20.4 mM). This mixture was incubated at 50 °C and the reaction was followed by ^{19}F NMR spectroscopy. A new signal appeared at –133.67 ppm, corresponding to aldehyde **8** (Figure S17). No further changes were observed after 1.5 h.

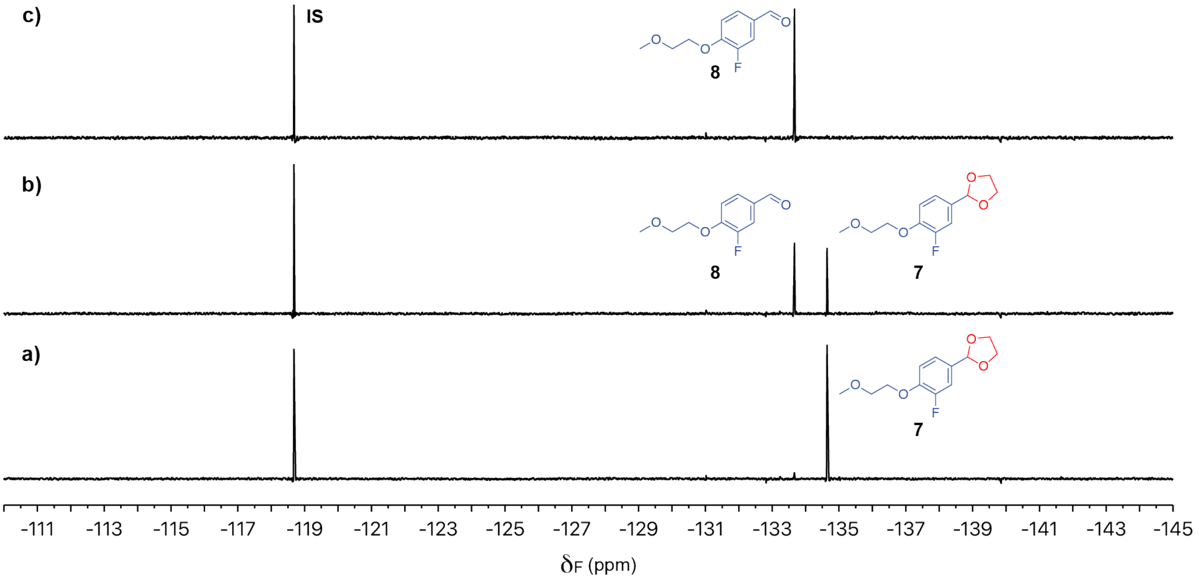

Figure S17. In situ monitoring of acetal hydrolysis from compound **7** to compound **8** by ^{19}F NMR spectroscopy (470 MHz, DMF/D_2O 97:3 *v*/*v*, d1 = 25 s). a) compound **7** (5.50 mM); b) reaction mixture incubated at 50 °C for 10 min after addition of CF_3CO_2H (20.4 mM), indicating a 1:1 ratio of **7** and **8**; c) compound **8** (5.50 mM). IS: internal standard (4-fluorotoluene, 5.00 mM).

7. Kinetic studies of acetal hydrolysis

7.1 Experimental protocol for kinetic experiments

Reactions were carried out in mixtures of freshly distilled DMF and D_2O, at 50 °C in an NMR tube. The reaction solvent mixture was first prepared, and 4-fluorotoluene added as an internal standard of known concentration (5.00 mM). Concentrations of all fluorine-containing species were determined by quantitative ^{19}F NMR spectroscopy by comparison to the signal for 4-fluorotoluene.

A stock solution of CF_3CO_2H was freshly prepared in the reaction solvent containing 4-fluorotoluene as internal standard (5.00 mM) and concentration measured by quantitative ^{19}F NMR spectroscopy.

A solution of AuNP or model compound of concentration ca. 5 mM was prepared in the reaction solvent (600 μL). This solution was incubated at 50 °C for 10 min before hydrolysis was initiated by adding an aliquot of the CF_3CO_2H stock solution so as to reach the desired concentration of acid. Reactions were followed by quantitative ^{19}F NMR spectroscopy until the acetal peak was no longer detectable.

7.2 Summary of kinetic data

Table S3. Pseudo-first-order rate constants for acetal hydrolysis on model compounds and AuNPs. Conditions: $[Acetal]_0$ ~ 5 mM; $[CF_3CO_2H]$ = 20 mM; 50 °C; distilled DMF/D_2O *v/v* 97:3.

Replicate	Compound **5** k / h^{-1}	AuNP-**1** k / h^{-1}	Compound **7** k / h^{-1}	AuNP-**2** k / h^{-1}
1	3.88 ± 0.06	0.61 ± 0.02	3.71 ± 0.06	0.67 ± 0.01
2	3.69 ± 0.06	0.64 ± 0.02	3.74 ± 0.09	0.70 ± 0.03
3	3.60 ± 0.07	0.71 ± 0.02	3.47 ± 0.10	0.65 ± 0.02
Average	3.72 ± 0.06	0.65 ± 0.02	3.64 ± 0.08	0.67 ± 0.02

Increasing the water concentration to 10% D_2O/DMF, the hydrolysis of AuNP-**2** was complete after only 100 min at 50 °C (Figure S18). Quantitative analysis afforded a rate constant (Table S4) that is consistent with a first-order dependence on water concentration. Likewise, nanoparticle-bound acetal hydrolysis kinetics also showed a first-order dependence on acid concentration (Figure S19, Table S5).

Table S4. Effect of water concentration on pseudo-first-order rate constants for acetal hydrolysis on AuNP-**2**. Conditions: $[Acetal]_0$ ~ 5 mM; $[CF_3CO_2H]$ = 20 mM; 50 °C.

Solvent mixture	k / h^{-1}
DMF/D_2O 97:3 *v/v*	0.67 ± 0.02
DMF/D_2O 90:10 *v/v*	2.43 ± 0.03

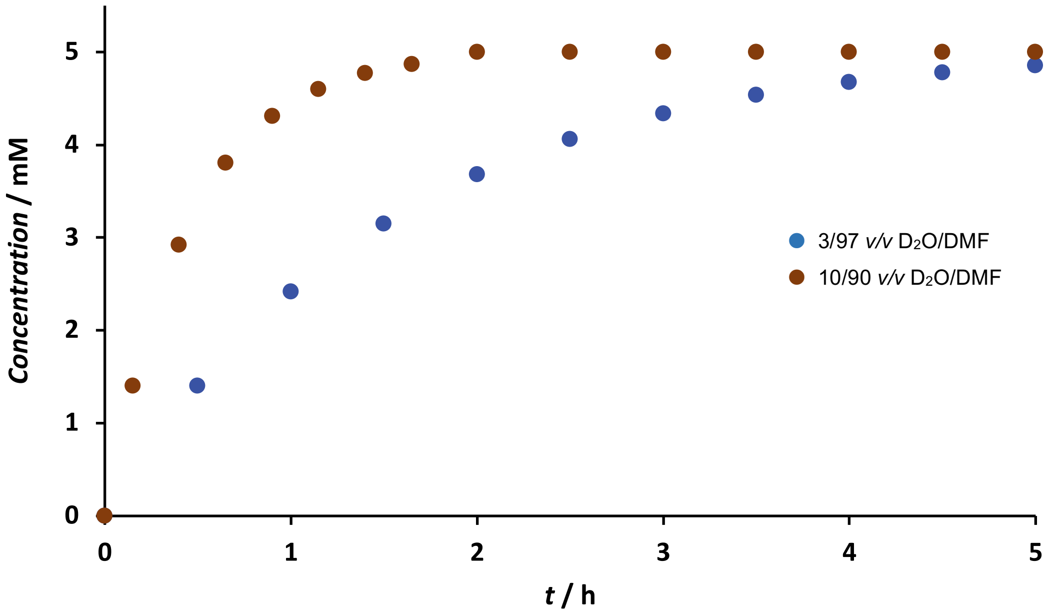

Figure S18. Kinetic profiles comparing nanoparticle-bound acetal hydrolysis at different water concentrations in the solvent media. Conditions: $[Acetal]_0$ ~ 5 mM; $[CF_3CO_2H]$ = 20 mM; 50 °C. Blue circles (●): 3% D_2O in DMF. Brown circles (●), 10% D_2O in DMF.

Table S5. Effect of acid concentration on pseudo-first-order rate constants for acetal hydrolysis on AuNP-**2**. Conditions: $[\text{Acetal}]_0$ ~ 5 mM; 50 °C; distilled DMF/D_2O 97:3 *v*/*v*.

$[CF_3CO_2H]$ / mM	k / h^{-1}
12.0 mM	0.45 ± 0.01
20.0 mM	0.67 ± 0.02

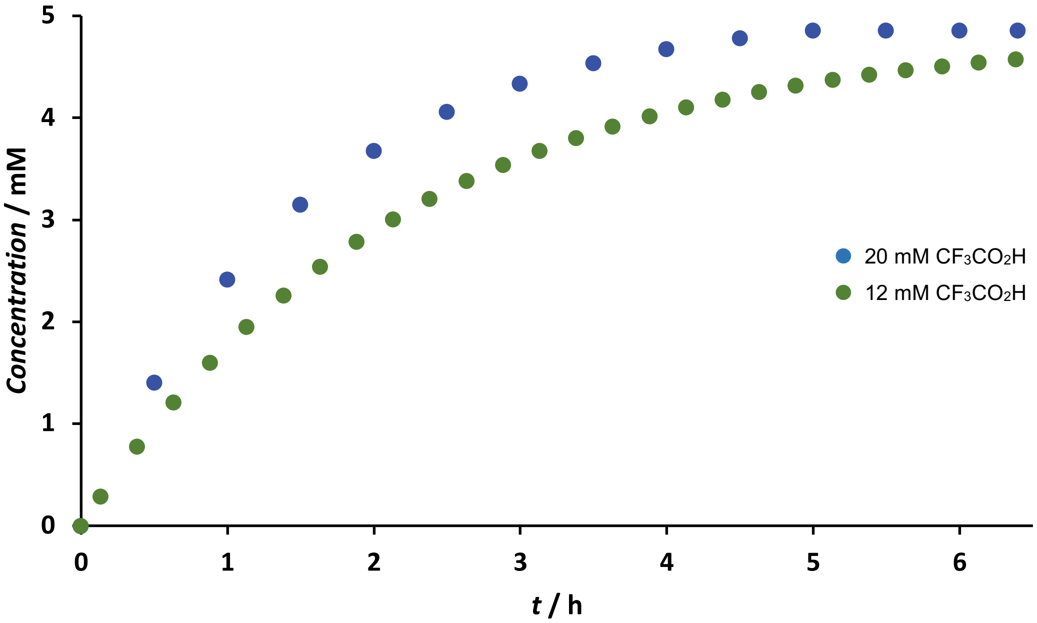

Figure S19. Kinetic profiles for comparing nanoparticle-bound acetal hydrolysis at different acid concentrations. Conditions: $[\text{Acetal}]_0$ ~5 mM; D_2O/DMF 3:97 *v*/*v*; 50 °C. Blue circles (●): $[CF_3CO_2H]$ = 20 mM (ca. 4 equiv. with respect to **2**). Green circles (●): $[CF_3CO_2H]$ = 12 mM (ca. 2.5 equiv. with respect to **2**).

8. One-step dynamic covalent modification of acetal-functionalized nanoparticles with nucleophilic modifiers

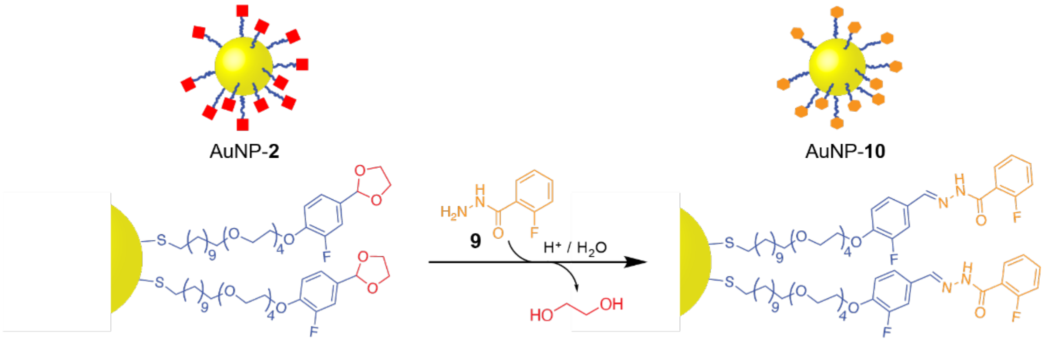

Concentrations of all fluorine-containing species were determined by quantitative ^{19}F NMR in the presence of 4-fluorotoluene as an internal standard of known concentration.
A stock solution of CF_3CO_2H was prepared in 9:1 *v*/*v* distilled DMF/D_2O with 4-fluorotoluene as internal standard (5.00 mM) and concentration measured by ^{19}F NMR.

Generation of AuNP-10
A colloidal solution of AuNP-**2** (7.29 mM) containing 4-fluorotoluene (5.00 mM) as internal standard was prepared in distilled DMF/D_2O (9:1 *v*/*v*, 600 μL). Then, an aliquot of hydrazide **9** stock solution (1.00 M, 27 μL) is added, followed by an aliquot of the CF_3CO_2H stock solution (1.388 M, 8.65 μL), giving final concentrations of hydrazide **9** (11.2 mM) and CF_3CO_2H (21.5 mM). This mixture was incubated at room temperature and the reaction was followed by ^{19}F NMR spectroscopy (Figure S20). No further changes were observed after 24 h.
Nanoparticles were precipitated by adding Et_2O/EtOH (8:1 *v*/*v*, 10 mL). The black solid recovered was resuspended in Et_2O/EtOH (7:1 *v*/*v*, 8 mL), sonicated for 10 min and recollected by centrifugation (1312 ×g rcf, 4 °C, 20 min). This operation was repeated a further twice. Traces of volatile solvents were removed from the purified residue under a stream of compressed air.

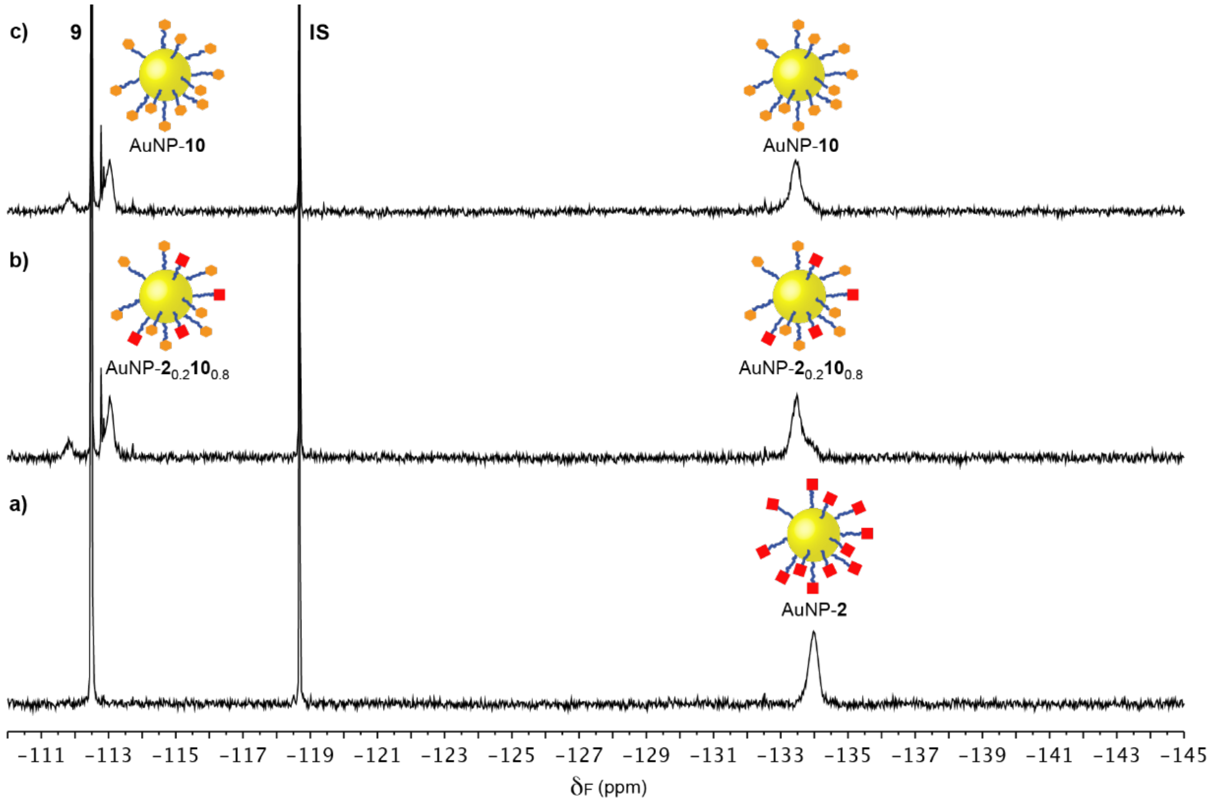

Figure S20. In situ monitoring of dynamic transformation from AuNP-**2** to AuNP-**10** by ^{19}F NMR spectroscopy (470 MHz, distilled DMF/D_2O 9:1 *v*/*v*, d1 = 25 s). a) Starting mixture of AuNP-**2** (7.29 mM) and hydrazide **9** (11.2 mM); b) Reaction mixture incubated at room temperature for 21 h after addition of CF_3CO_2H (21.5 mM), indicating a 2:8 ratio of **2** and **10** surface-bound acetal and hydrazone ligands, respectively; c) reaction mixture 24 h after addition of CF_3CO_2H, indicating quantitative conversion to AuNP-**10** (7.25 mM). IS: internal standard (4-fluorotoluene, 5.00 mM). Two signals are assigned to surface-bound hydrazone **10** corresponding to two hydrazone rotamers.

9. Two-step dynamic covalent modification of acetal-functionalized nanoparticles with nucleophilic modifiers

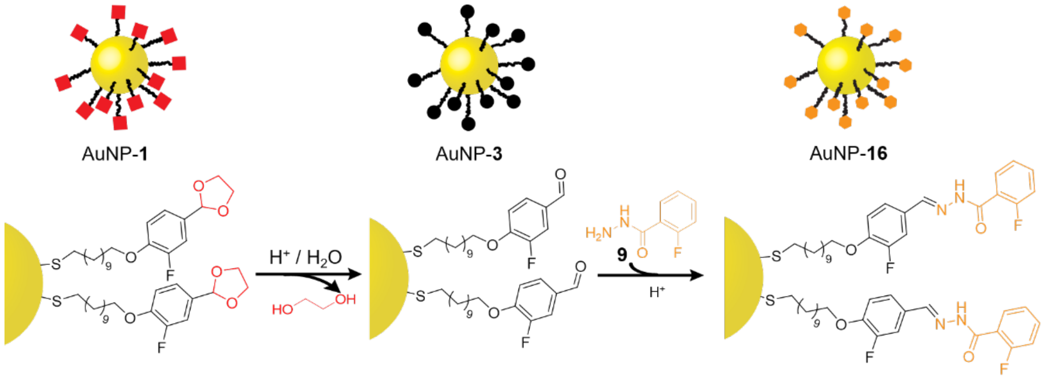

Step 1

Stock solutions containing 4-fluorotoluene (5.00 mM) as internal standard were prepared in D_2O/DMF (3:97 *v*/*v*) and concentrations of fluorine-containing species determined by quantitative ^{19}F NMR spectroscopy.

Solution **A**: $[CF_3CO_2H]$ = 1030 mM.

Solution **B**: [AuNP-**1**] = 7.93 mM in terms of surface bound acetal **1**

Solution **B** (600 μL) was heated to 50 °C then an aliquot of solution **A** (18.5 μL) was added, giving final concentrations of [AuNP-**1**] = 7.69 mM and $[CF_3CO_2H]$ = 30.8 mM. The mixture was held at 50 °C and monitored by ^{19}F NMR spectroscopy at regular intervals. A new broad nanoparticle-bound signal appeared at –133.07 ppm, corresponding to nanoparticle-bound aldehyde **3** (Figure S21). No further changes were observed after 9 h.

Nanoparticles were precipitated by adding Et_2O/MeOH (4:1 *v*/*v*, 10 mL). The black solid recovered was resuspended in Et_2O/MeOH (4:1 *v*/*v*, 15 mL), sonicated for 10 min and recollected by centrifugation (1446 ×g rcf, 4 °C, 10 min). This operation was repeated a further twice. Traces of volatile solvents were removed from the purified residue under a stream of compressed air. Oxidative ligand desorption confirmed the composition of nanoparticle-bound monolayer as AuNP-**3**.

Step 2

Stock solutions containing 4-fluorotoluene (5.00 mM) as internal standard were prepared in DMF and concentrations determined by quantitative ^{19}F NMR spectroscopy.

Solution **C**: $[CF_3CO_2H]$ = 190 mM.

Solution **D**: [2-fluorobenzohydrazide, **9**] = 513 mM.

Solution **E**: [AuNP-**3**] = 3.75 mM in terms of surface bound aldehyde **3**.

Solution **E** was incubated at 50 °C then aliquots of solution **C** (11.8 μL) and **D** (13.2 μL) were added, giving final concentrations of [AuNP-**3**] = 3.60 mM, $[CF_3CO_2H]$ = 3.60 mM and [2-fluorobenzohydrazide] = 10.8 mM. The mixture was held at 50 °C and monitored by ^{19}F NMR spectroscopy at regular intervals. Two new broad nanoparticle-bound signals appeared at –133.46 ppm and –112.83 ppm, corresponding to the two hydrazone rotamers of nanoparticle-bound **16** (Figure S21). No further changes were observed after 5 h.

Nanoparticles were precipitated by adding Et_2O/MeOH (4:1 *v*/*v*, 10 mL). The black solid recovered was resuspended in Et_2O/MeOH (4:1 *v*/*v*, 15 mL), sonicated for 10 min and recollected by centrifugation (1446 ×g rcf, 4 °C, 10 min). This operation was repeated further twice. Traces of volatile solvents were removed from the purified residue under a stream of compressed air. Oxidative ligand desorption revealed that the composition of nanoparticle-bound monolayer corresponded to hydrazone **16** (Figure S22).

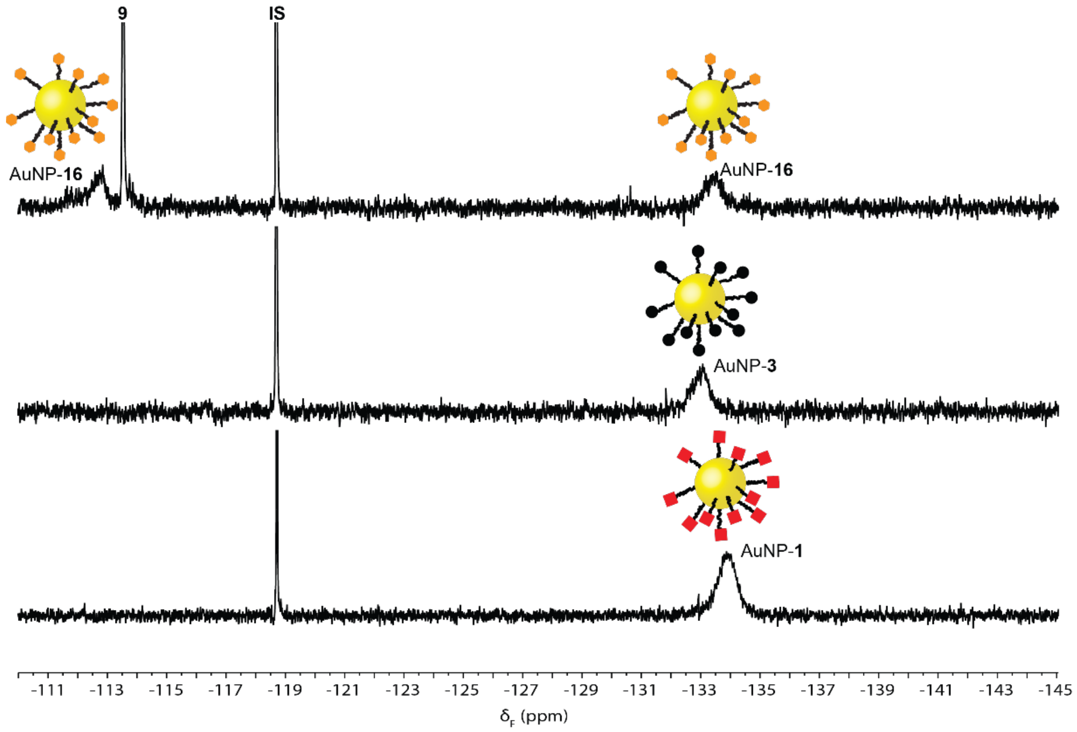

Figure S21. In situ monitoring of two-step transformation from AuNP-**2** to AuNP-**16** via AuNP-**3** by ^{19}F NMR spectroscopy (470 MHz, DMF/D_2O 97/3 *v*/*v*, d1 = 25 s). Bottom: AuNP-**1** (3.75 mM); middle: reaction mixture incubated at 50 °C for 9 h after addition of CF_3CO_2H; top: reaction mixture incubated at 50 °C for 5 h after mixing AuNP-**3** (3.60 mM), CF_3CO_2H (3.60 mM) and 2-fluorobenzohydrazide **9** (10.8 mM). IS: internal standard (4-fluorotoluene, 5.00 mM).

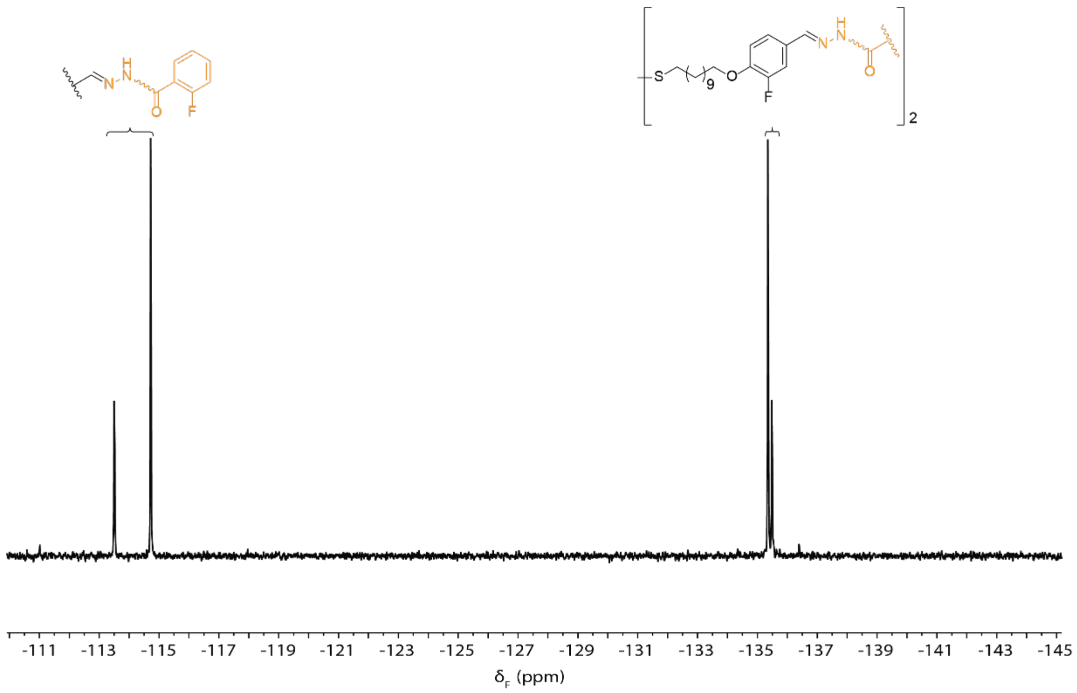

Figure S22. ^{19}F NMR spectrum (470 MHz, DMF-d_7) of oxidative ligand desorption performed on a solution of purified AuNP-**16**.

10. Imine constitutional reorganization on AuNP-3

Stock solutions containing 4-fluorotoluene as internal standard (5.00 mM) were prepared in DMF/THF (1:1 *v*/*v*). Concentrations of fluorine-containing species were determined by quantitative ^{19}F NMR spectroscopy, and other species gravimetrically.

Solution **A**: [CF_3CO_2H] = 476 mM.

Solution **B**: [CF_3CO_2H] = 47.6 mM (prepared by diluting a portion of solution **A** 10 times)

Solution **C**: [octylamine] = 500 mM

Solution **D**: [1,8-Diazabicyclo[5.4.0]undec-7-ene] = 500 mM

Solution **E**: [AuNP-**3**] = 8.24 mM (in terms of nanoparticle-bound aldehyde **3**).

Solution **E** (600 µL) was incubated at 50 °C then neat 4-fluorobenzylamine (1.1 µL) and aliquots of solution **B** (10.3 µL) and **C** (19.7 µL) were added, giving final concentrations of [AuNP-**3**] = 7.83 mM, [octylamine, **11**] = 15.6 mM, [4-fluorobenzylamine, **12**] = 17.1 mM and [CF_3CO_2H] = 0.780 mM. This mixture was incubated at 50 °C and the reaction followed by ^{19}F NMR spectroscopy. A new broad nanoparticle-bound signal appeared at –133.66 ppm, corresponding to the benzylidene portion of both AuNP-bound imines formed by octylamine and 4-fluorobenzylamine. Another broad signal appeared at –116.38 ppm, corresponding to the terminal fluorine environment of the AuNP-bound 4-fluorobenzylamine imine, allowing calculation of the relative concentration of each nanoparticle-bound imine by signal area deconvolution (Table S6, Figure 2b). No further changes were observed after 38 h.

An aliquot of solution **A** (27.9 µL) was then added to the reaction mixture, to give a total of 2.8 molar equivalents CF_3CO_2H (with respect to nanoparticle-bound **3**). After incubation at 50 °C for a further 1 h, ^{19}F NMR analysis revealed that the broad peak at –116.38 ppm had increased in intensity, indicating the increase of 4-fluorobenzylamine imine (and decrease of octylamine imine) in the monolayer. No further changes were observed after 1 h. After this time, solution **D** (26.6 µL, 2.7 mol equiv.) was added to the reaction mixture. The broad peak at –116.38 ppm in the ^{19}F NMR spectrum recorded after 1 h at 50 °C had decreased, indicating the increase of octylamine imine (and decrease of 4-fluorobenzylamine imine) in the monolayer.

The switching cycle to high and then low acid concentrations was repeated a further once (Figure S23).

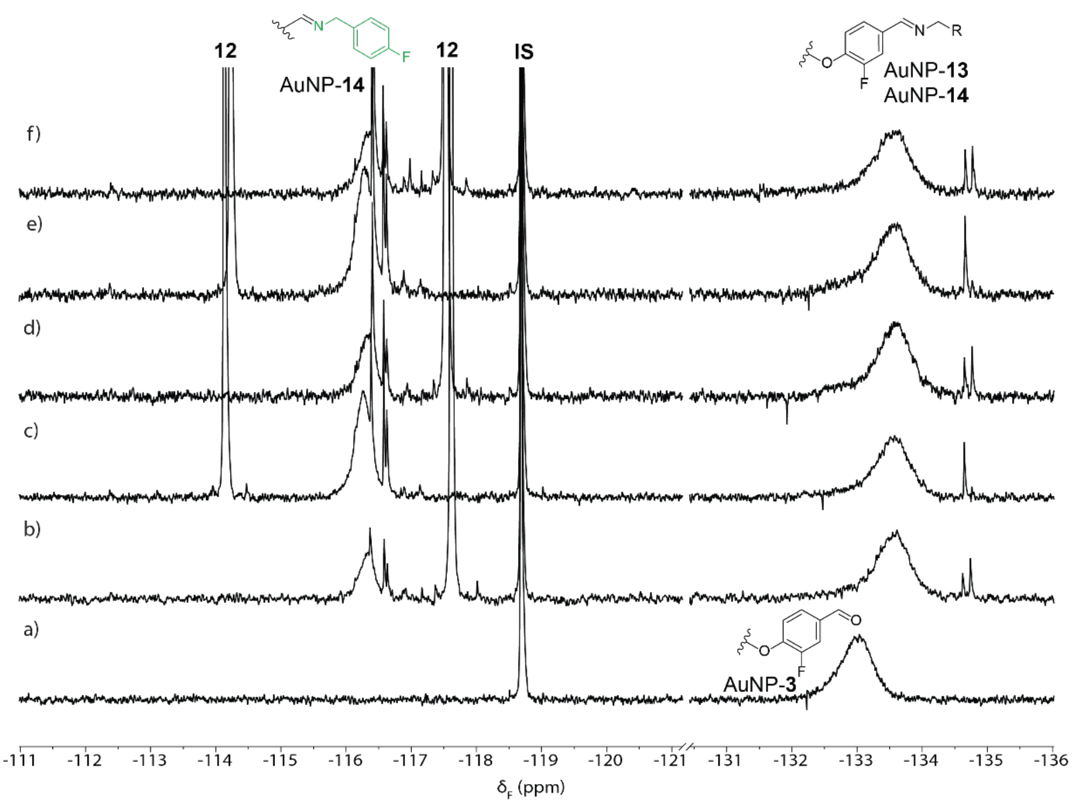

Figure S23. In situ monitoring of AuNP-bound imine exchange by ^{19}F NMR spectroscopy (470 MHz, DMF/THF 1:1 *v*/*v*, d1 = 25 s). (a) AuNP-**3** (8.24 mM); (b) reaction mixture incubated at 50 °C for 38 h after addition of CF_3CO_2H (0.1 equiv.), 4-fluorobenzylamine (2 equiv.) and octylamine (2 equiv.); (c) reaction mixture incubated at 50 °C for 1 h after addition of CF_3CO_2H (2.7 equiv.); (d) reaction mixture incubated at 50 °C for 1 h after addition of DBU (2.7 equiv.); (e) reaction mixture incubated at 50 °C for 1 h after addition of CF_3CO_2H (2.5 equiv.); (f) reaction mixture incubated at 50 °C for 1 h after addition of DBU (2.5 equiv.). IS: internal standard (4-fluorotoluene, 5.00 mM).

11. Imine constitutional reorganization in model system

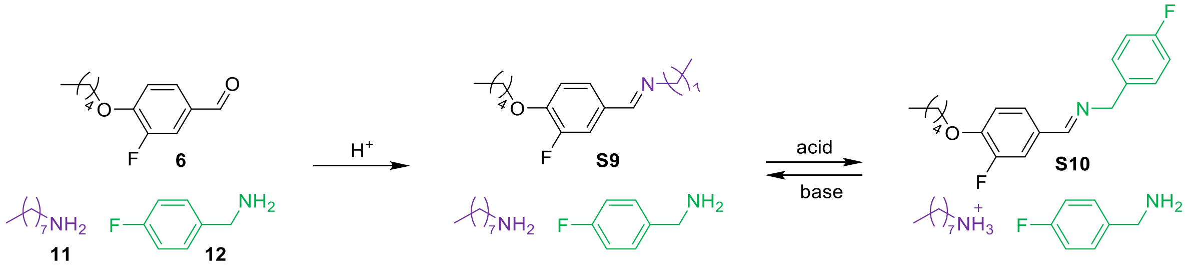

Stock solutions containing 4-fluorotoluene as internal standard (5.00 mM) were prepared in DMF/THF (1:1 *v*/*v*). Concentrations of fluorine-containing species were determined by quantitative ^{19}F NMR spectroscopy, and other species gravimetrically.

Solution **A**: [CF_3CO_2H] = 448 mM.

Solution **B**: [CF_3CO_2H] = 44.8 mM (prepared by diluting a portion of solution **A** 10 times).

Solution **C**: [4-fluorobenzylamine] = 541 mM.

Solution **D**: [octylamine] = 500 mM.

Solution **E**: [1,8-diazabicyclo[5.4.0]undec-7-ene, DBU] = 500 mM.

Solution **F**: [aldehyde] = 7.29 mM (in terms of aldehyde compound **6**).

Solution **F** (600 µL) was incubated at 50 °C then aliquots of solution **B** (9.8 µL), solution **C** (16.2 µL) and solution **D** (17.5 µL) were added, giving final concentrations of aldehyde **6** (6.80 mM), 4-fluorobenzylamine (13.6 mM), octylamine (13.6 mM) and CF_3CO_2H (0.680 mM). This mixture was incubated at 50 °C and the reaction followed by ^{19}F NMR spectroscopy. A new signal appeared at –134.80 ppm, corresponding to the benzylidene portion of compound **S9**. Two new signals appeared at –134.68 ppm and –116.64 ppm, corresponding to the benzylidene portion and the terminal fluorine environment of compound **S10**, allowing calculation of the relative concentration of each imine by signal area deconvolution (Table S6, Figure S24). No further changes were observed after 19 h.

An aliquot of solution **A** (24.4 µL) was then added to the reaction mixture, to give a total of 2.6 molar equivalents CF_3CO_2H (with respect to **6**). After incubation at 50 °C for a further 0.5 h, ^{19}F NMR analysis revealed that the signals at –134.68 ppm and –116.64 ppm have increased in intensity (while the peak at –134.80 decreased in intensity), indicating the increase of **S10** (and decrease of **S9**) in the solution. No further changes were observed after 0.5 h. After this time, solution **E** (21.9 µL, 2.5 mol equiv.) was added to the reaction mixture. The signals peak at –134.68 ppm and –116.64 ppm in the ^{19}F NMR spectrum recorded after 0.5 h at 50 °C had decreased, (while the peak at –134.80 increased), indicating the increase of **S9** (and decrease of **S10**) in the solution.

The switching cycle to high and then low acid concentrations was repeated a further once (Figure S25).

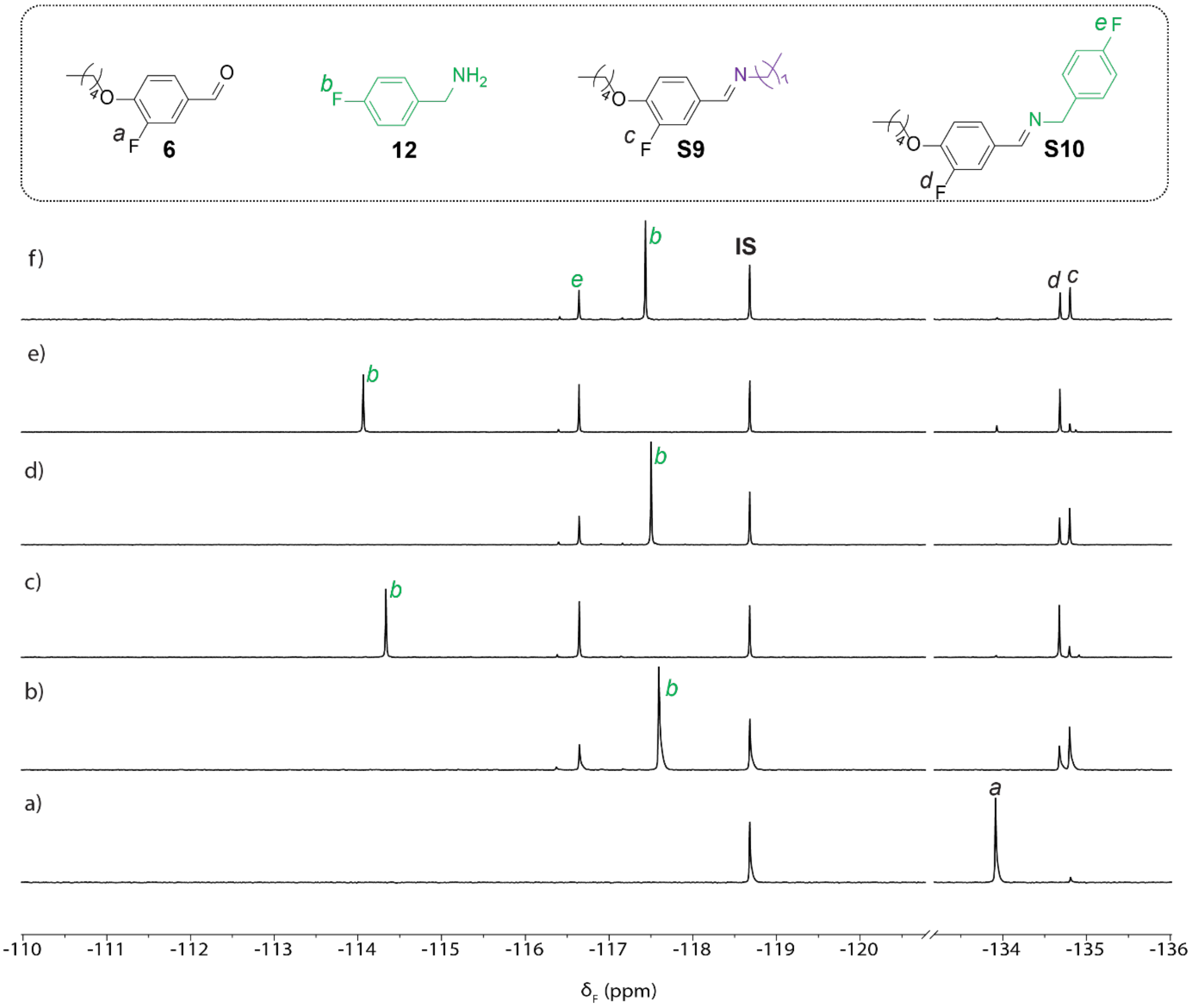

Figure S24. In situ monitoring of solution-phase imine exchange by ^{19}F NMR spectroscopy (470 MHz, DMF/THF 1:1 *v*/*v*, d1 = 25 s): a) Model aldehyde compound **6** (7.29 mM); b) reaction mixture incubated at 50 °C for 19 h after addition of CF_3CO_2H (0.1 equiv.), 4-fluorobenzylamine (2 equiv.) and octylamine (2 equiv.); c) reaction mixture incubated at 50 °C for 0.5 h after addition of CF_3CO_2H (2.5 equiv.); d) reaction mixture incubated at 50 °C for 0.5 h after addition of DBU (2.5 equiv.); e) reaction mixture incubated at 50 °C for 0.5 h after addition of CF_3CO_2H (2.5 equiv.); f) reaction mixture incubated at 50 °C for 0.5 h after addition of DBU (2.5 equiv.). IS: internal standard (4-fluorotoluene, 5.00 mM).

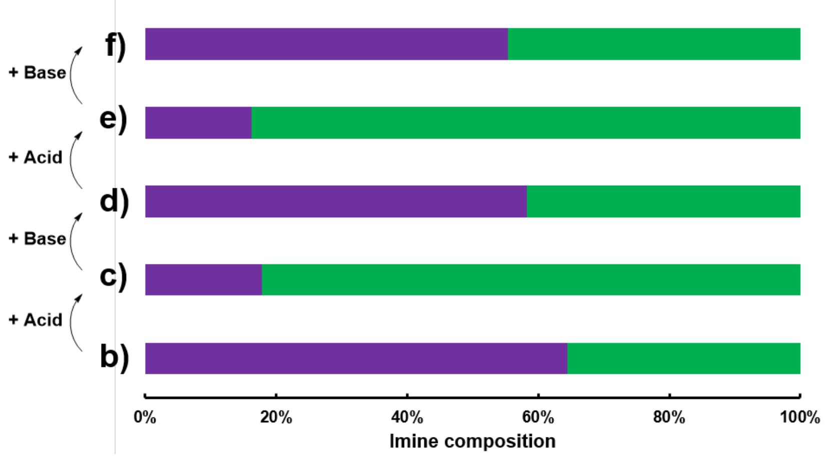

Figure S25. Imine composition in response to changes concentration of acid and base. b) Reaction mixture incubated at 50 °C for 19 h after addition of CF_3CO_2H (0.1 equiv.), 4-fluorobenzylamine (2 equiv.) and octylamine (2 equiv.); c) reaction mixture incubated at 50 °C for 0.5 h after addition of CF_3CO_2H (2.5 equiv.); d) reaction mixture incubated at 50 °C for 0.5 h after addition of DBU (2.5 equiv.); e) reaction mixture incubated at 50 °C for 0.5 h after addition of CF_3CO_2H (2.5 equiv.); f) reaction mixture incubated at 50 °C for 0.5 h after addition of DBU (2.5 equiv.).

Table S6. Adaptive imine mixture compositions observed in bulk solution and nanoparticle-bound environments.

Cycle	Bulk solution<br>**S9**:**S10**	Nanoparticle-bound<br>AuNP-**13** : AuNP-**14**
b)	0.65 : 0.35	0.70 : 0.30
c)	0.18 : 0.82	0.20 : 0.80
d)	0.58 : 0.42	0.62 : 0.38
e)	0.16 : 0.84	0.16 : 0.84
f)	0.55 : 0.45	0.54 : 0.46

12. Generation and characterization of negatively charged AuNP-15

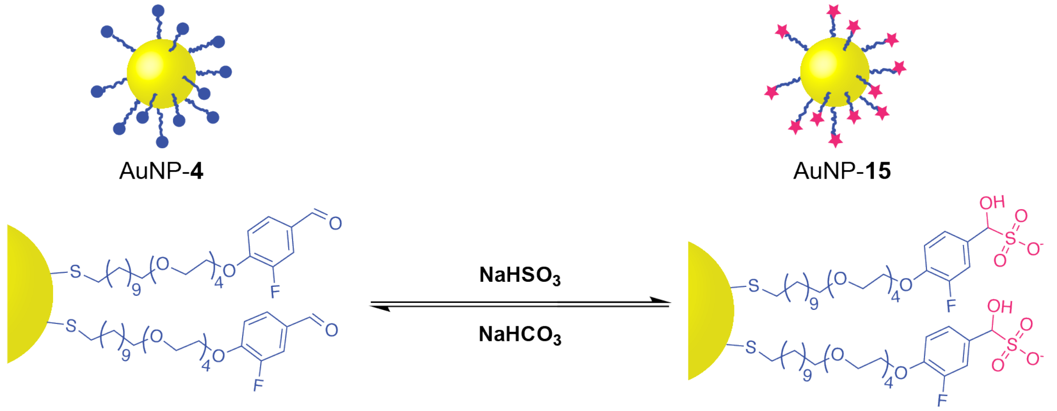

12.1 Rapid reversible solvophilicity switching in one phase

AuNP-**4** (ca. 1 mg) was placed in a glass vial with water (ca. 1 mL), in which they remained insoluble. Solid $NaHSO_3$ was added (ca. 2 mg) followed by sonication for 5 min, resulting in the dissolution of all nanoparticle material, consistent with the formation of negatively charged bisulfite adduct AuNP-**15**. Rapid decomposition back to the starting aldehyde was achieved on addition of solid $NaHCO_3$ (4 mg per 1 mg of AuNPs), resulting in complete re-precipitation of all nanoparticle material after 5 min. After washing with water to remove the excess of salts, the resulting black solid was solubilized organic solvent (CH_2Cl_2) indicating the recovery of the starting material AuNP-**4**.

12.2 Rapid reversible biphasic switching

AuNP-**4** (ca. 2 mg) was dissolved in CH_2Cl_2 (ca. 1 mL) in a glass vial and water (ca. 1 mL) was added with the consequent formation of two phases (left-hand image, Figure 3b). Solid $NaHSO_3$ (ca. 5.0 mg) was then added and the mixture was shaken by hand for 5 min, resulting in the transfer of the nanoparticles to the aqueous phase, indicating the formation of negatively charged bisulfite adduct AuNP-**15** (middle image, Figure 3b). Finally, solid $NaHCO_3$ (ca. 10 mg) was added, the biphasic mixture was shaken by hand (5 min), resulting in the transfer of the nanoparticles back to the organic phase, indicating the recovery of the starting material AuNP-**4** (right-hand image, Figure 3b).

12.3 NMR characterization of reversible biphasic switching

AuNP-**4** (ca. 5 mg) was dissolved in $CDCl_3$ (600 μL), and a ^1H NMR spectrum recorded (Figure S26a). The mixture was transferred to a glass vial and D_2O (600 μL) added with the consequent formation of two phases. Solid $NaHSO_3$ (11.0 mg) was then added and the mixture was shaken by hand for 5 min, followed by 5 min sonication, resulting in formation of an emulsion. Evaporating the organic solvent under a stream of air produced a clear dark reddish/brownish solution of AuNPs in D_2O. Analysis by ^1H NMR spectroscopy indicated formation of the bisulfite adduct AuNP-**15** (Figure S26b).
To the solution of AuNP-**15** in D_2O was then added $CDCl_3$ (600 μL), followed by solid $NaHCO_3$ (19 mg). The mixture was shaken by hand (5 min) followed by 5 min of sonication, resulting in formation of an emulsion.
Both solvents were evaporated under a stream of air. The black solid was washed with deionised water to eliminate the excess of salts, dried under a stream of air, then solubilized in $CDCl_3$ (600 μL). The ^1H NMR spectrum of the resulting solution (Figure S26c) indicated the presence of AuNP-**4**, confirming the reversibility of the switching process.

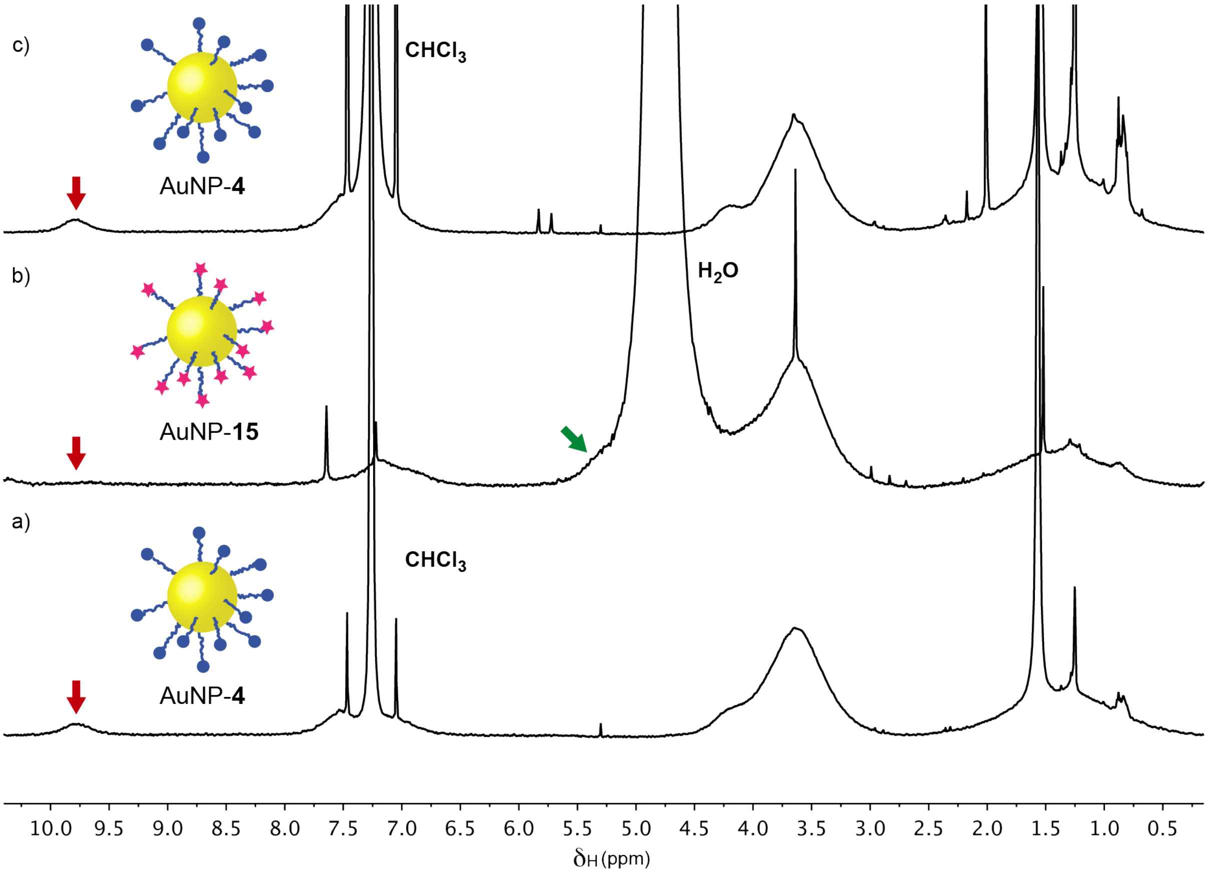

Figure S26. In situ monitoring of reversible solubility switching by ^1H NMR (500 MHz). a) AuNP-**4** in $CDCl_3$; b) AuNP-**15** in D_2O (green arrow at 5.45 ppm indicates the appearance of a new broad peak corresponding to the hydroxyl proton); c) AuNP-**4** in $CDCl_3$. Red arrow at 9.8 ppm indicates the disappearance and the reappearance of the proton aldehyde proton.

13. ^{1}H and ^{13}C NMR spectra of organic compounds

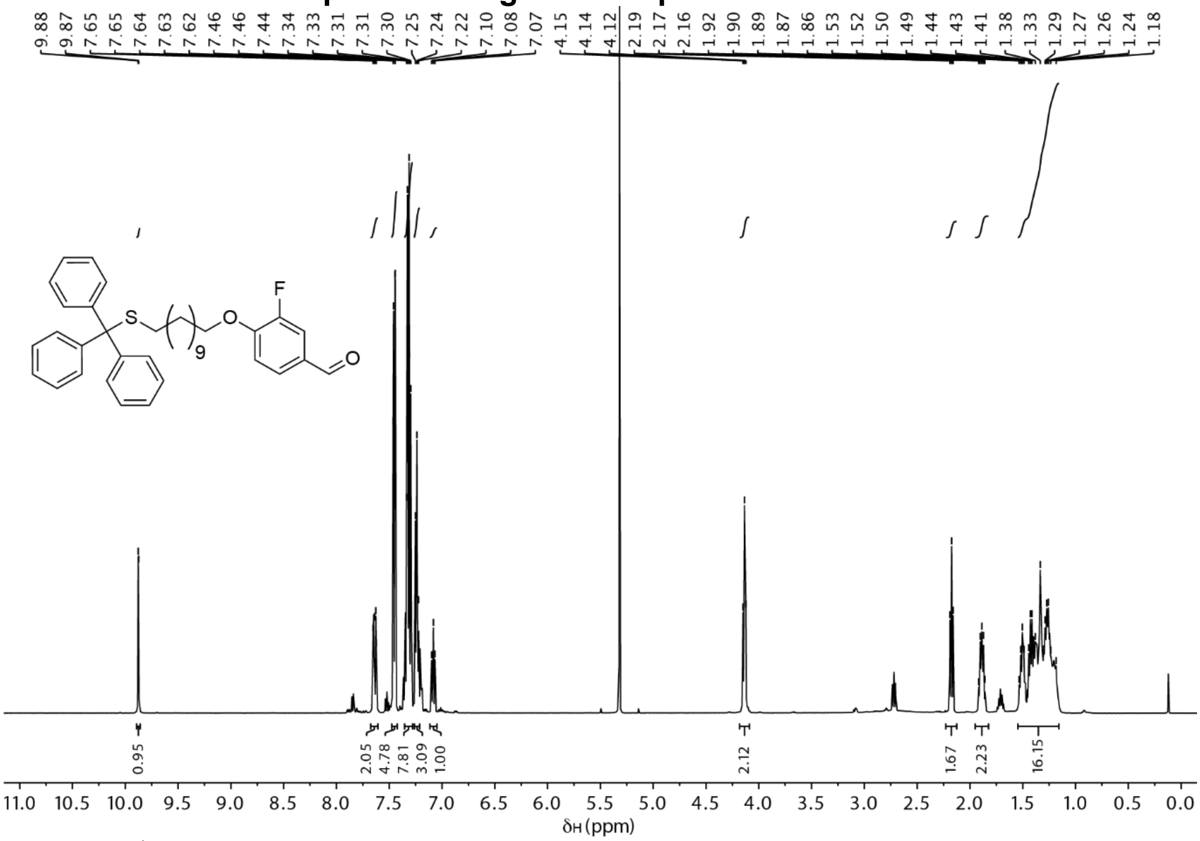

Figure S27. ^{1}H NMR spectrum of compound **S3** (400 MHz, $CDCl_3$).

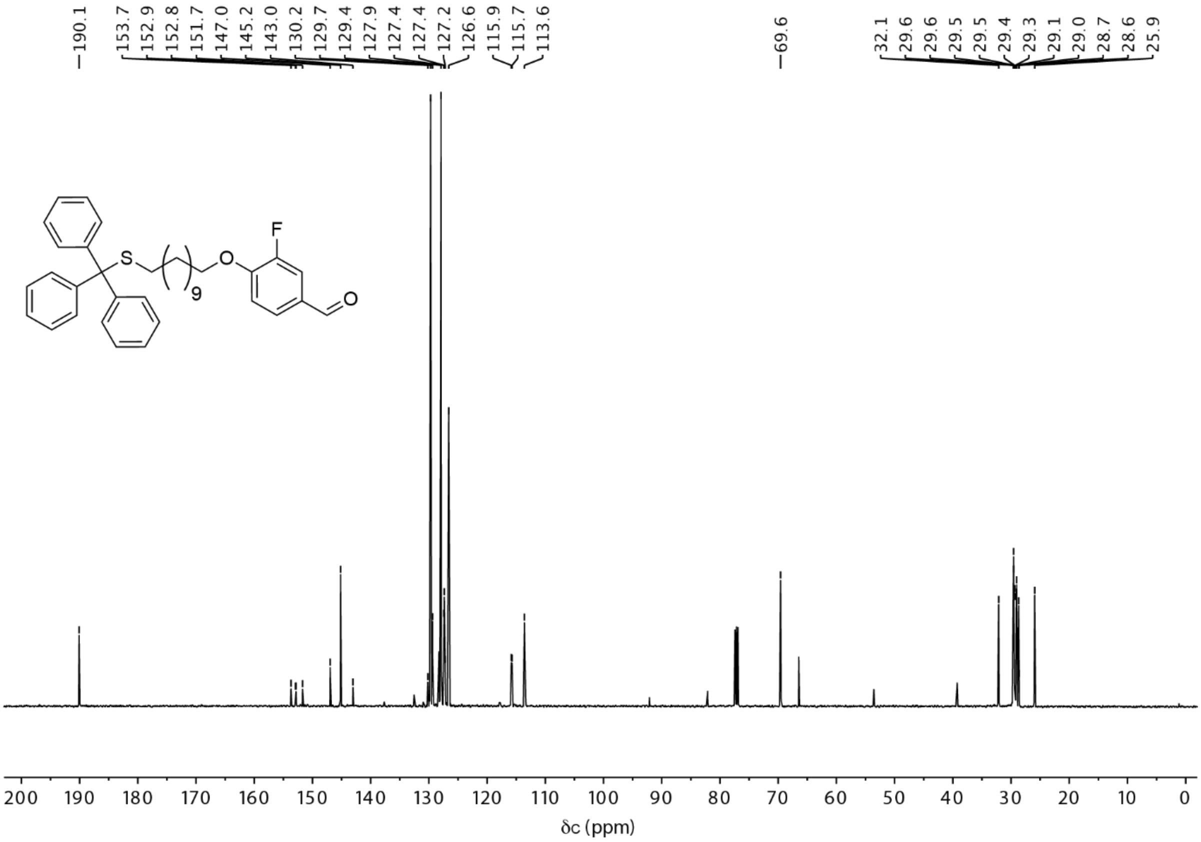

Figure S28. ^{13}C NMR spectrum of compound **S3** (126 MHz, $CDCl_3$).

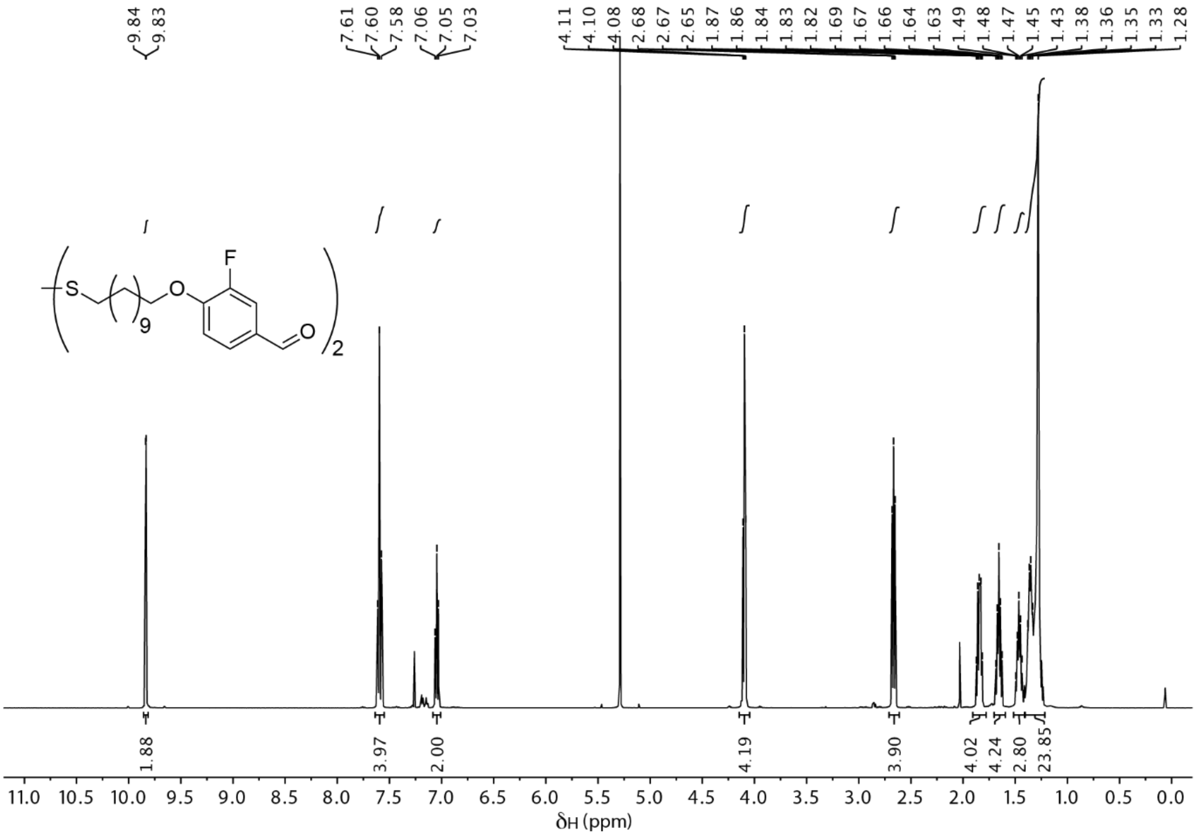

Figure S29. ^{1}H NMR spectrum of compound **S4** (500 MHz, $CDCl_3$).

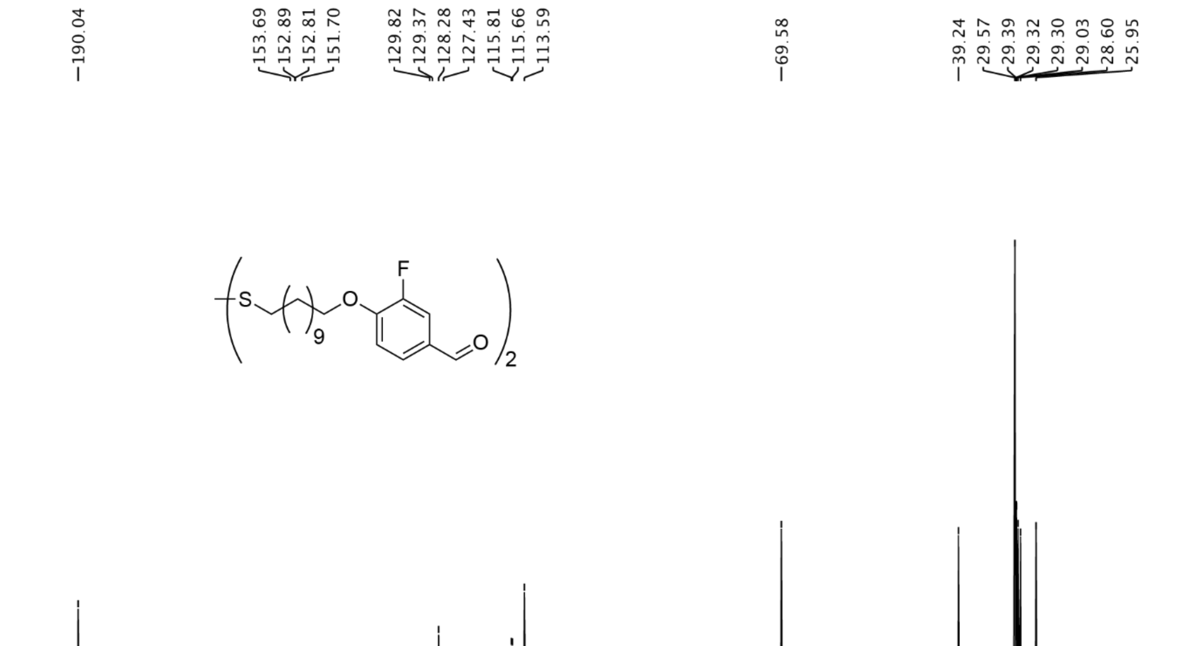

Figure S30. ^{13}C NMR spectrum of compound **S4** (126 MHz, $CDCl_3$).

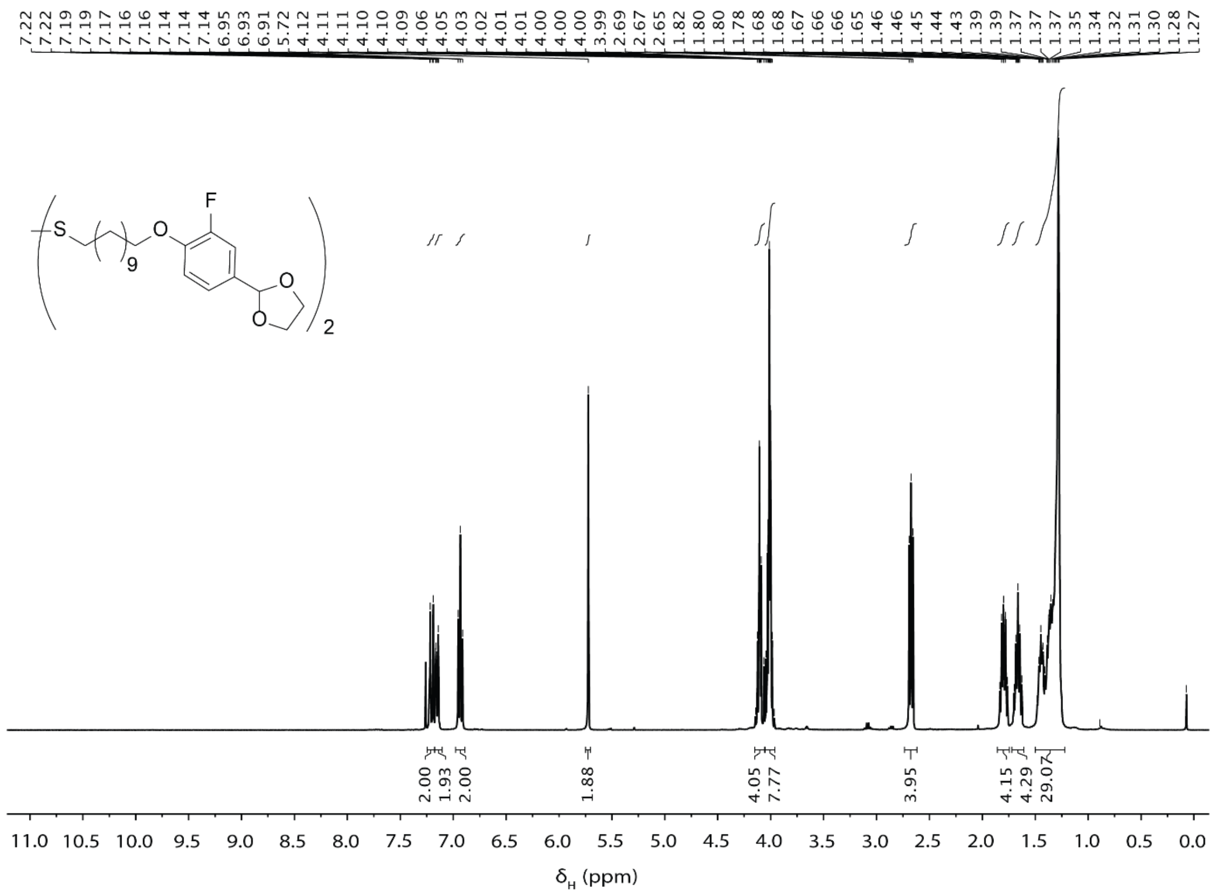

Figure S29. ^{1}H NMR spectrum of compound $\mathbf{1}_2$ (400 MHz, $CDCl_3$).

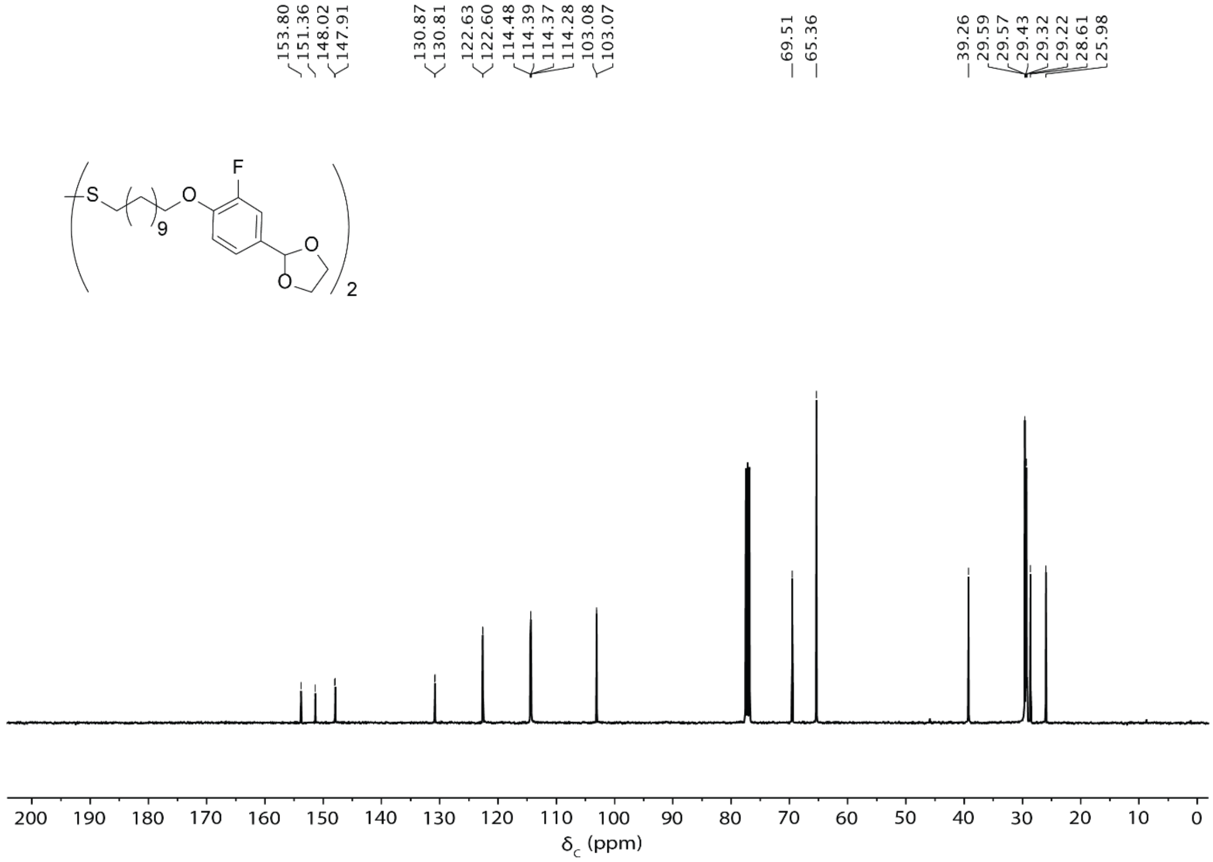

Figure S30. ^{13}C NMR spectrum of compound **1**$_2$ (101 MHz, $CDCl_3$).

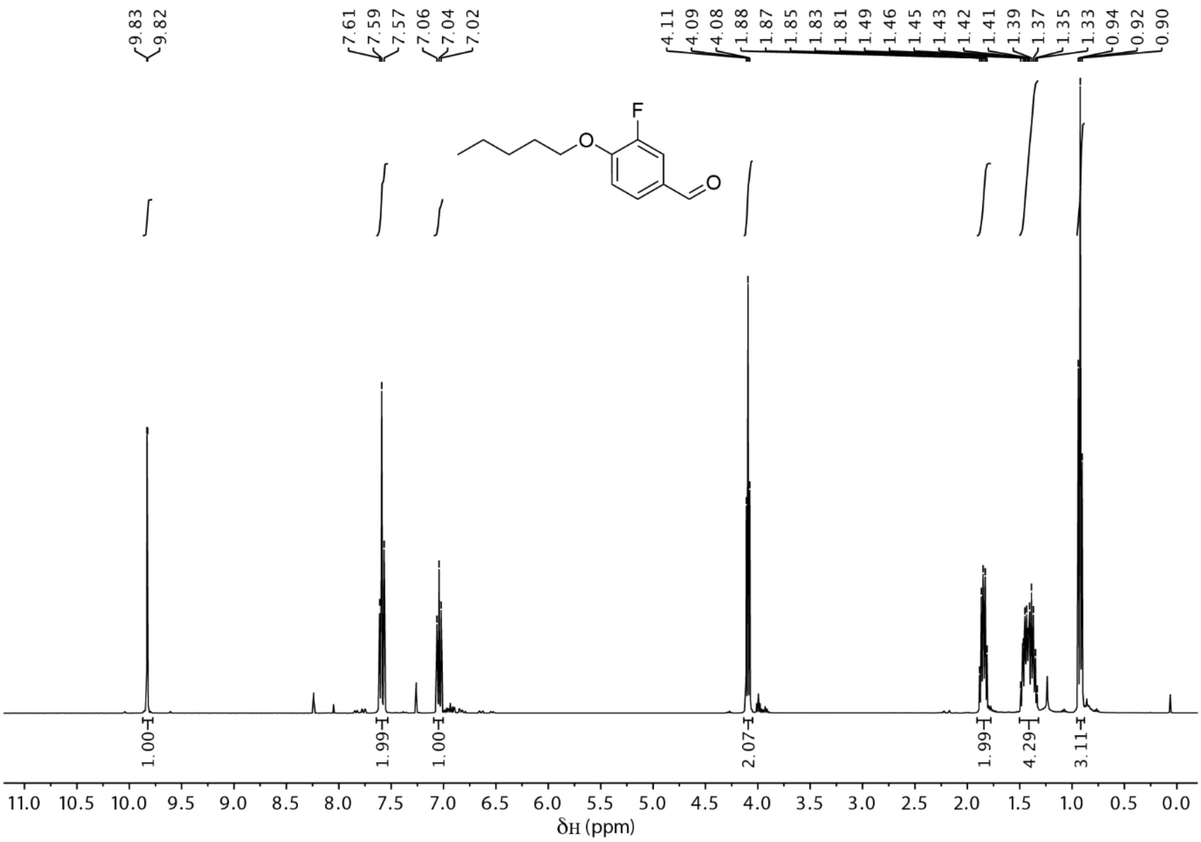

Figure S31. ^{1}H NMR spectrum of compound **6** (400 MHz, $CDCl_3$).

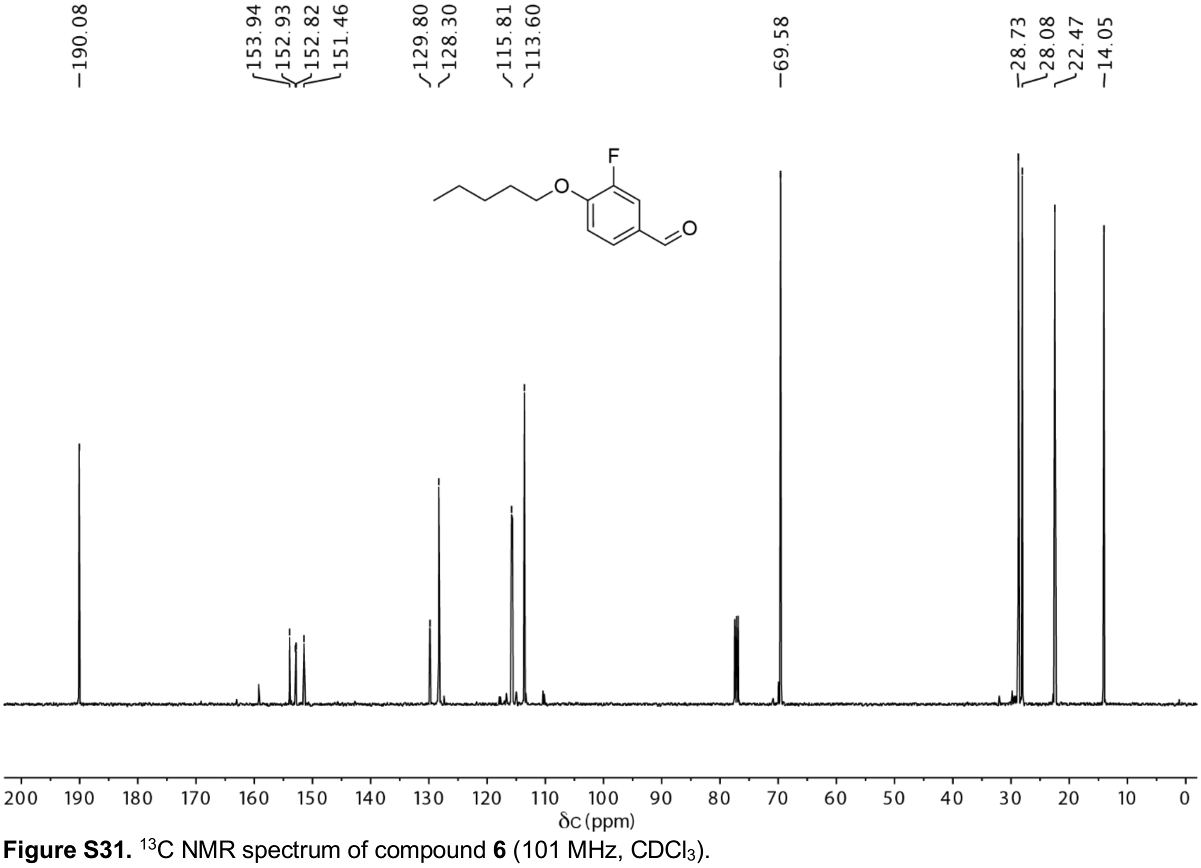

Figure S31. ^{13}C NMR spectrum of compound **6** (101 MHz, $CDCl_3$).

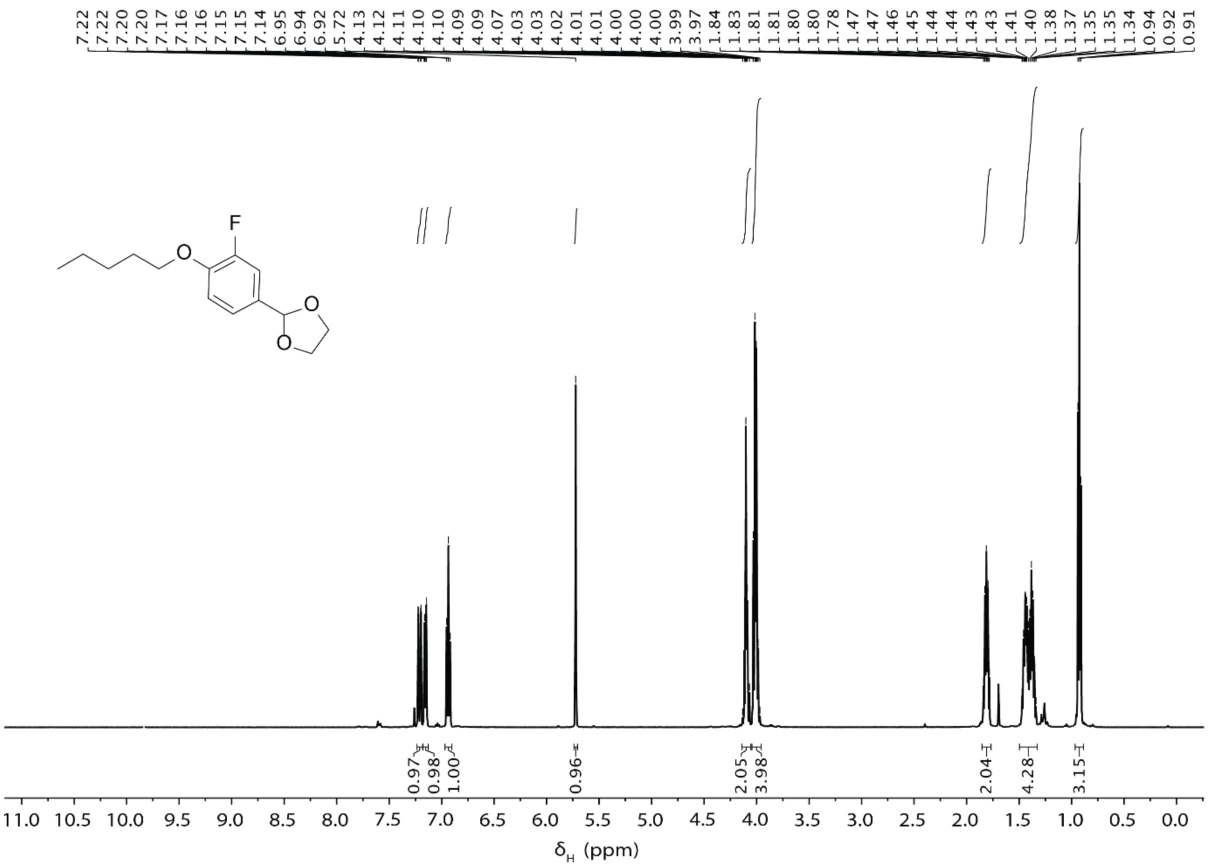

Figure S33. ^{1}H NMR spectrum of compound **5** (400 MHz, $CDCl_3$).

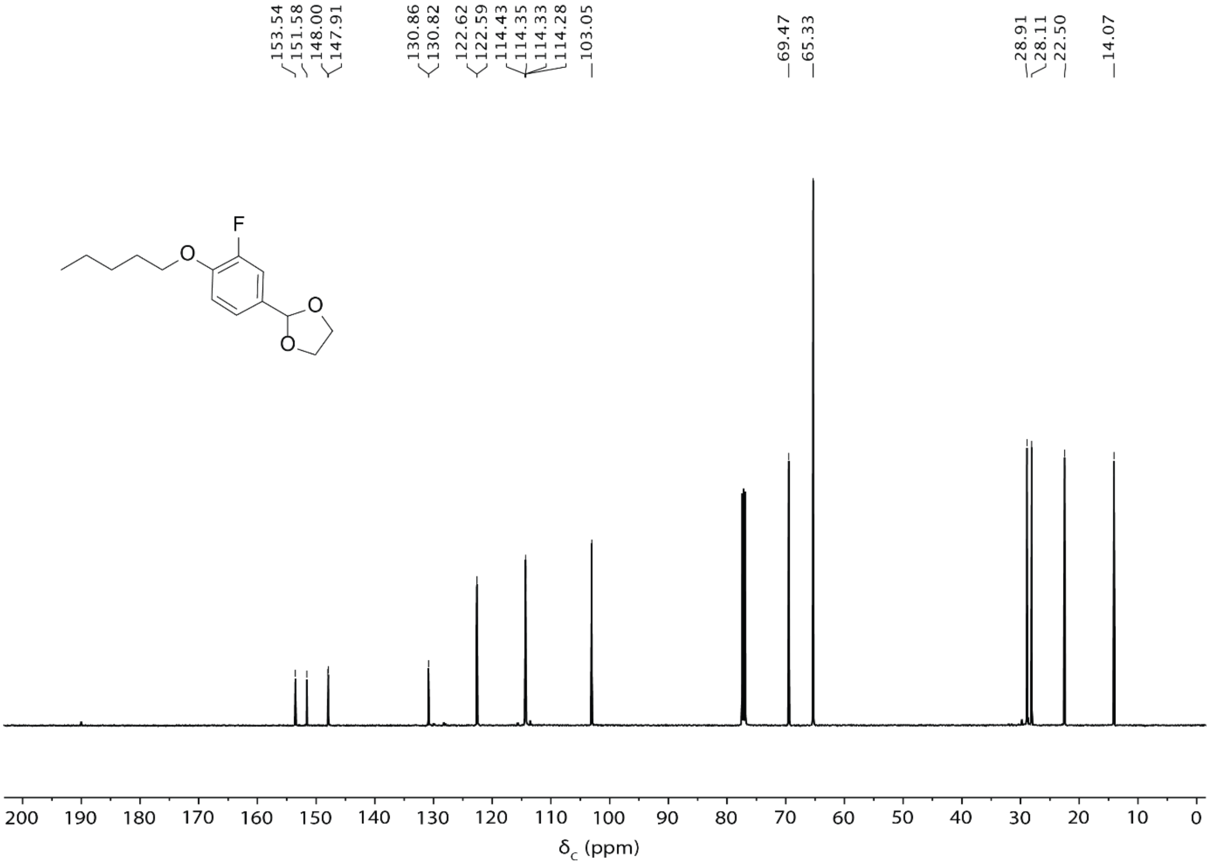

Figure S34. ^{13}C NMR spectrum of compound **5** (101 MHz, $CDCl_3$).

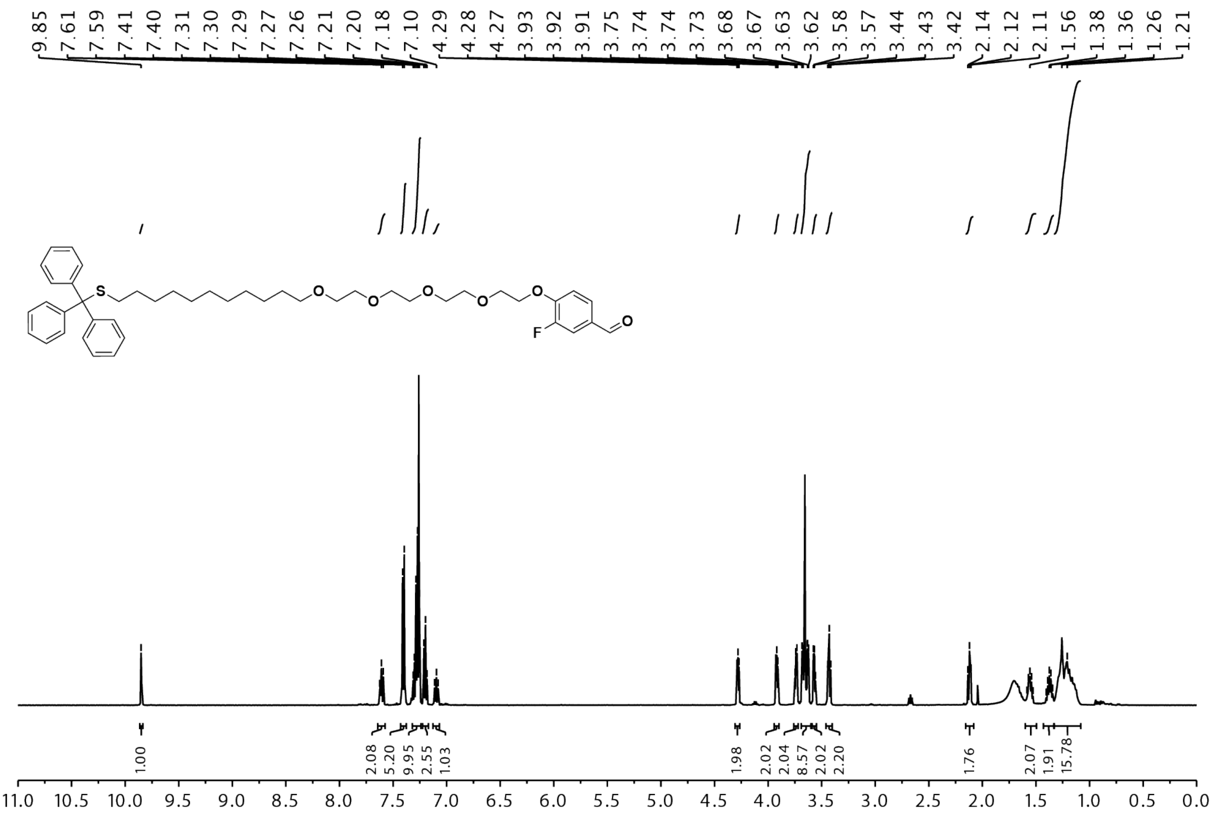

Figure S32. ^{1}H NMR spectrum of compound **S7** (500 MHz, $CDCl_3$).

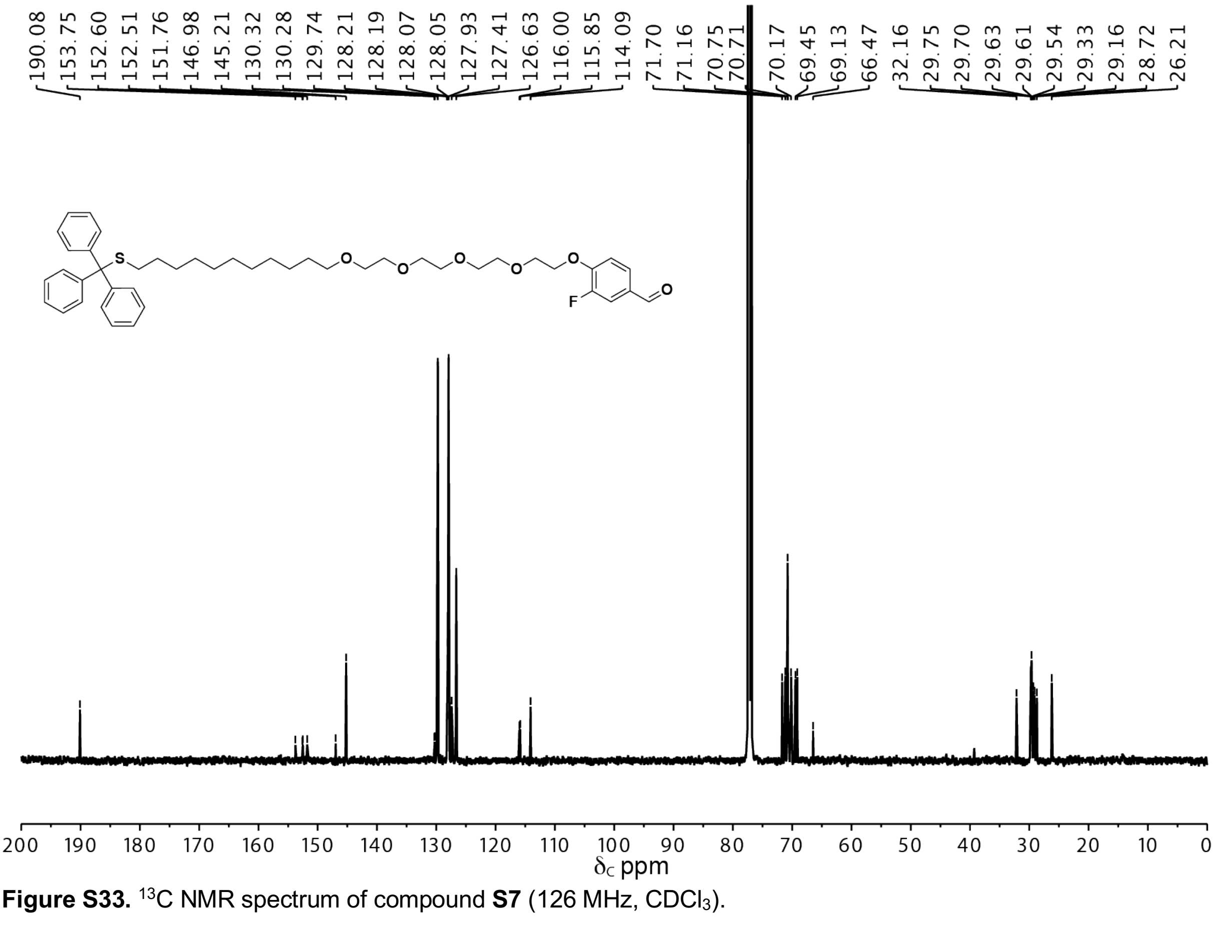

Figure S33. ^{13}C NMR spectrum of compound **S7** (126 MHz, $CDCl_3$).

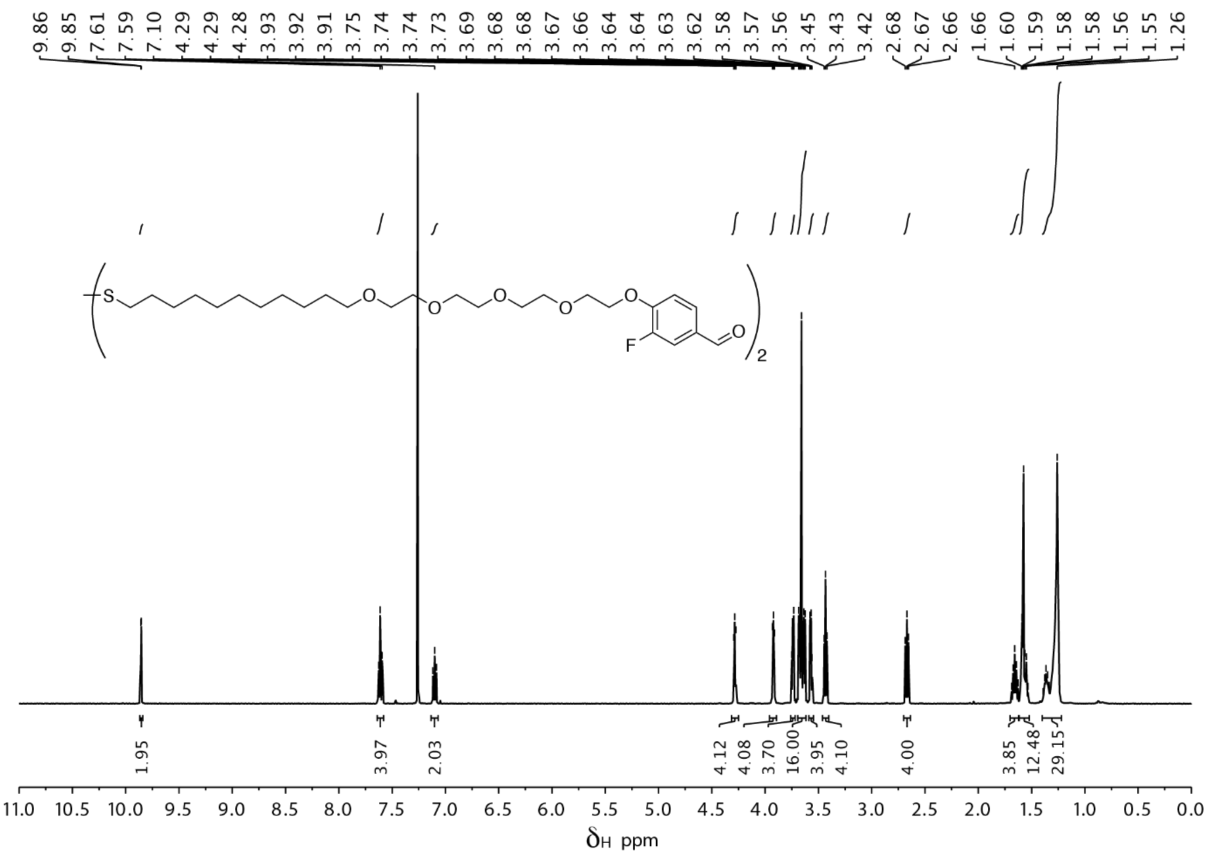

Figure S34. ^{1}H NMR spectrum of compound **S8** (500 MHz, $CDCl_3$).

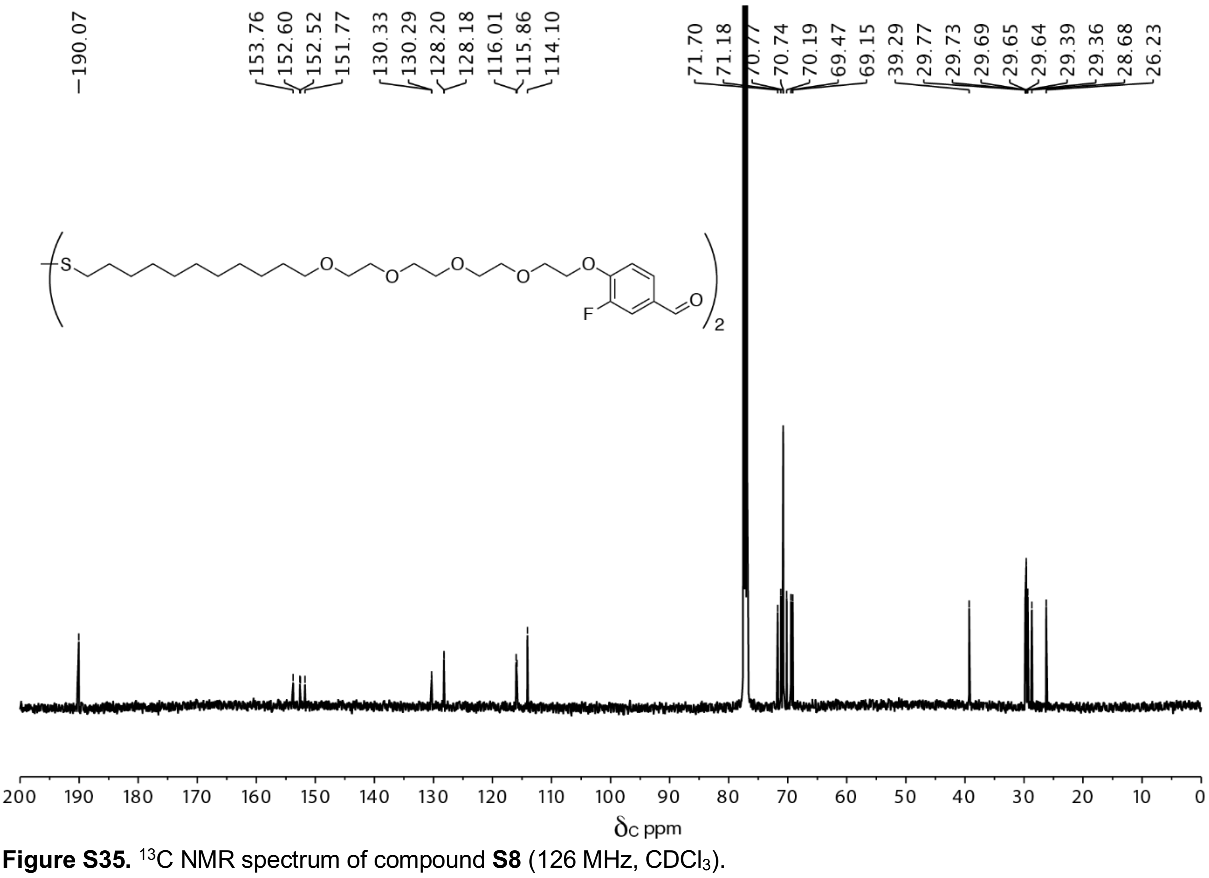

Figure S35. ^{13}C NMR spectrum of compound **S8** (126 MHz, $CDCl_3$).

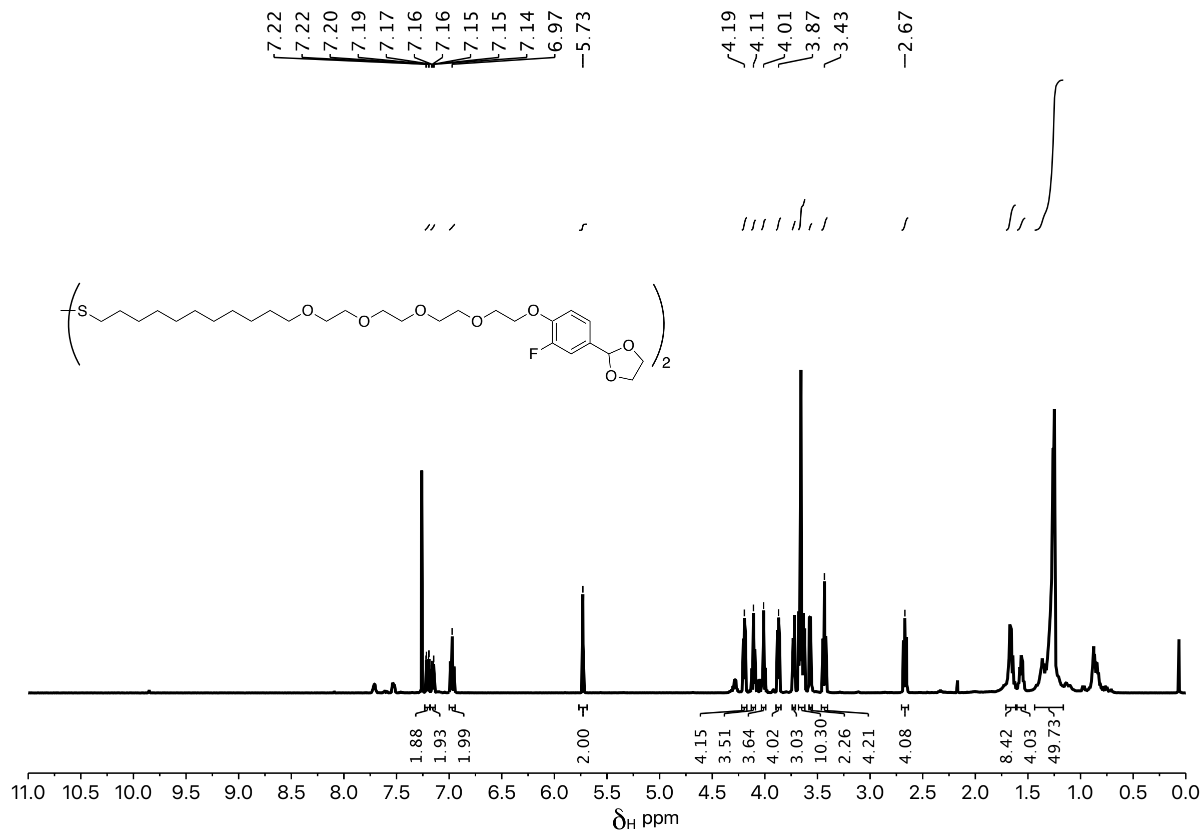

Figure S36. ^{1}H NMR spectrum of compound **2**$_{2}$ (500 MHz, $CDCl_3$).

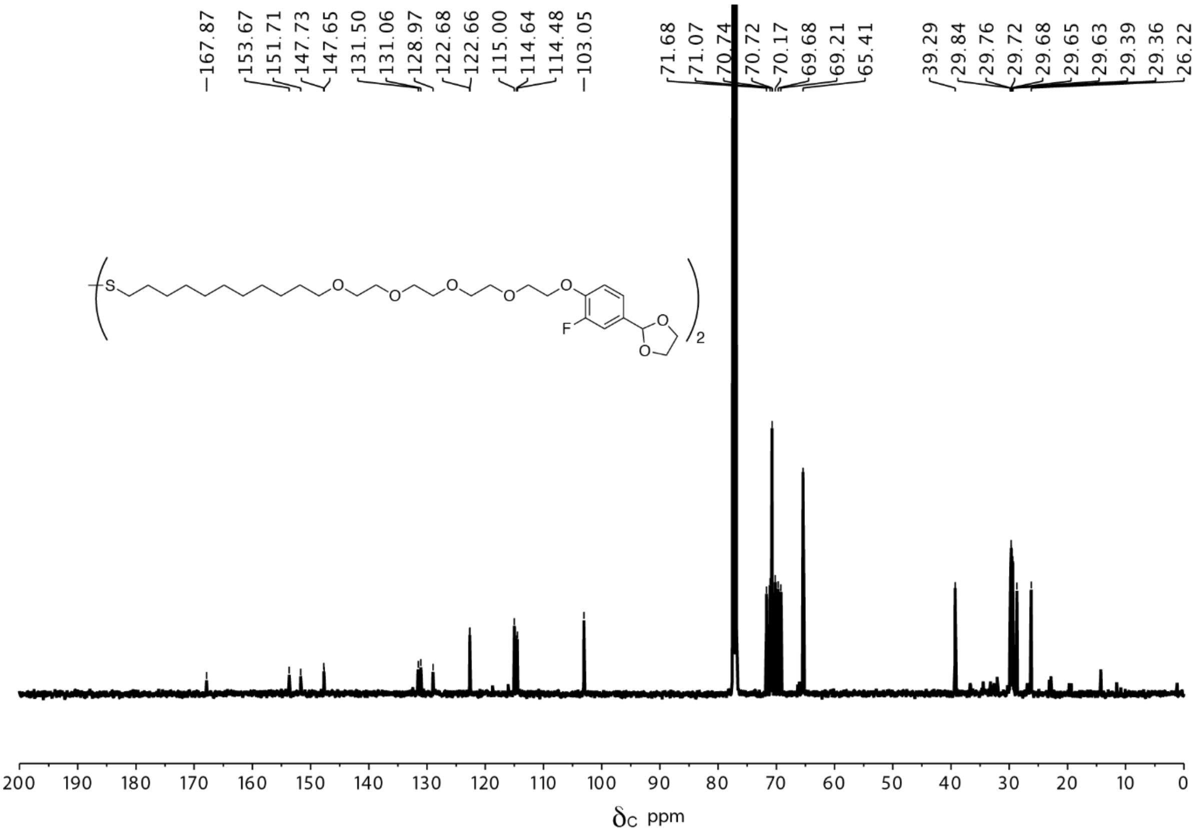

Figure S37. ^{13}C NMR spectrum of compound **2**$_{2}$ (126 MHz, $CDCl_3$).

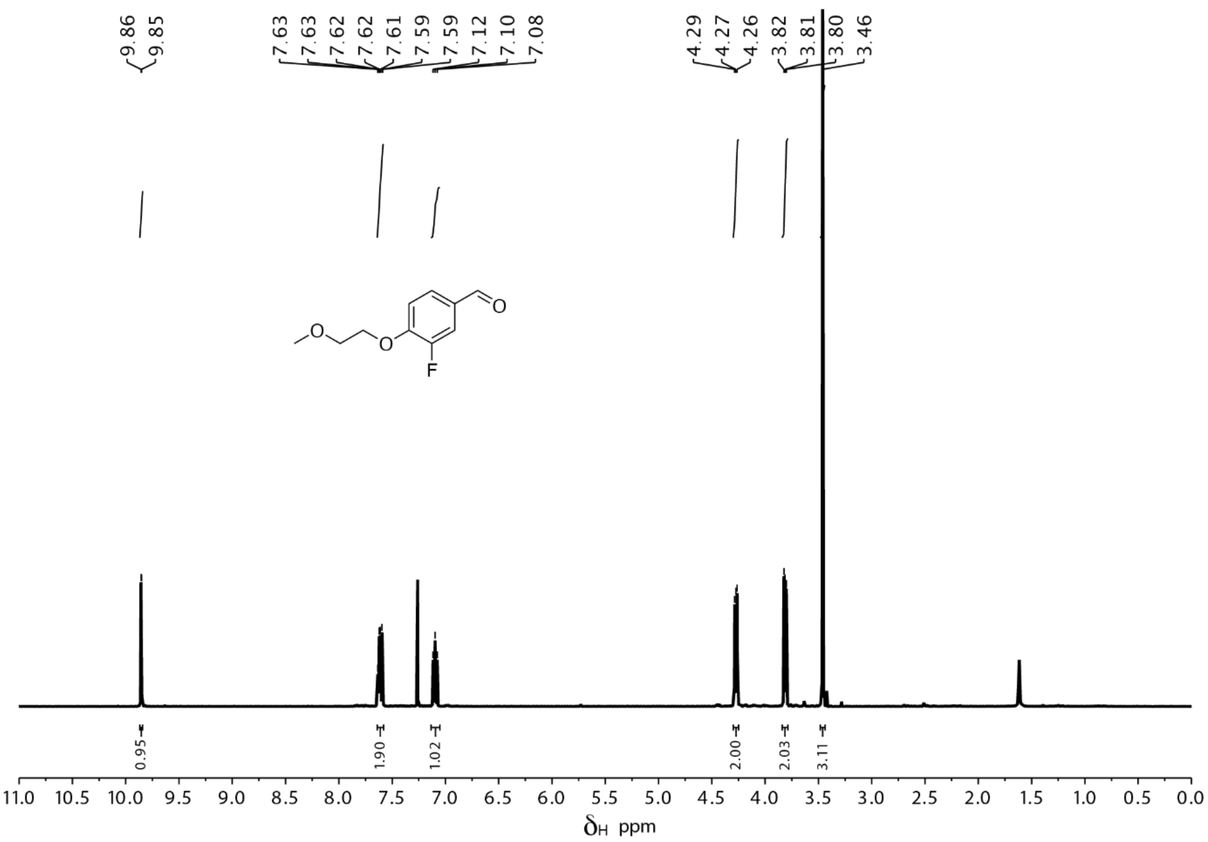

Figure S38. ^{1}H NMR spectrum of compound **8** (500 MHz, $CDCl_3$).

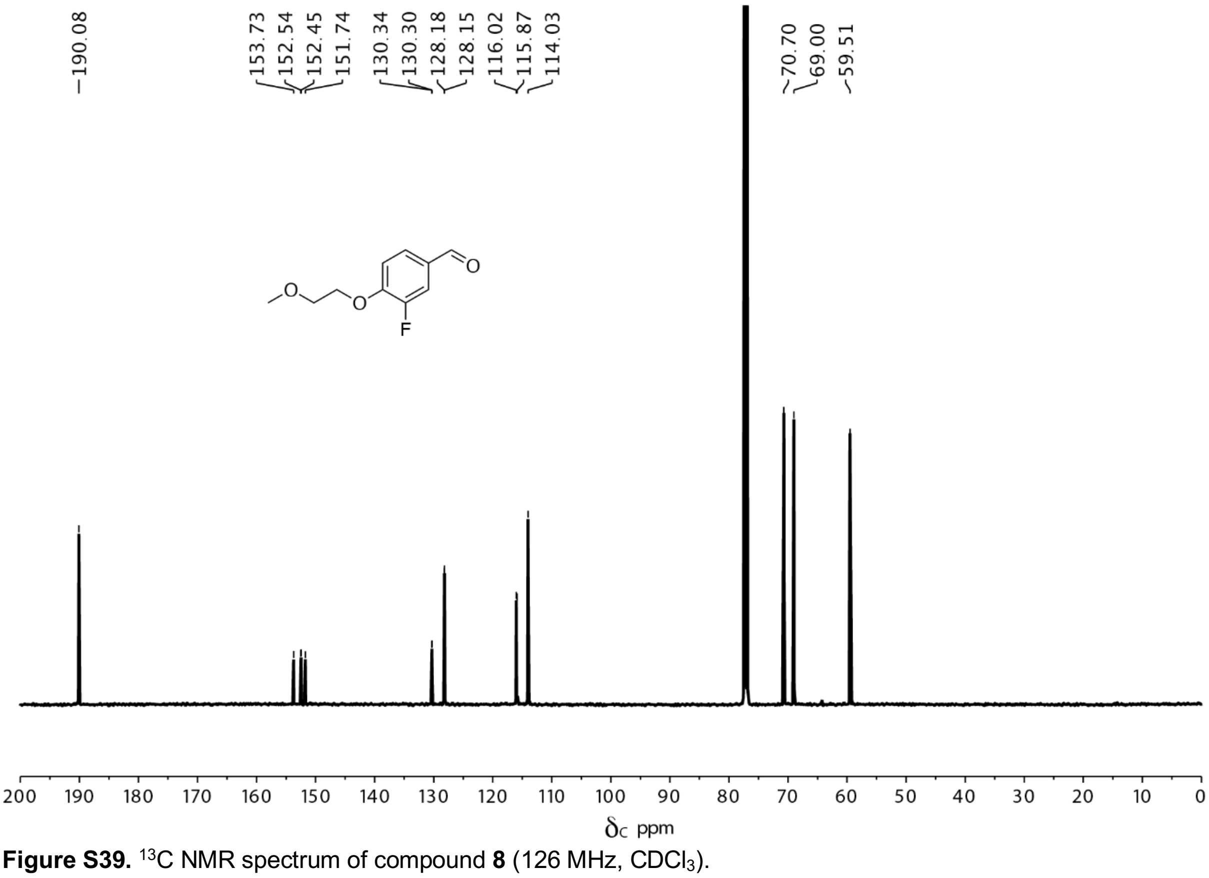

Figure S39. ^{13}C NMR spectrum of compound **8** (126 MHz, $CDCl_3$).

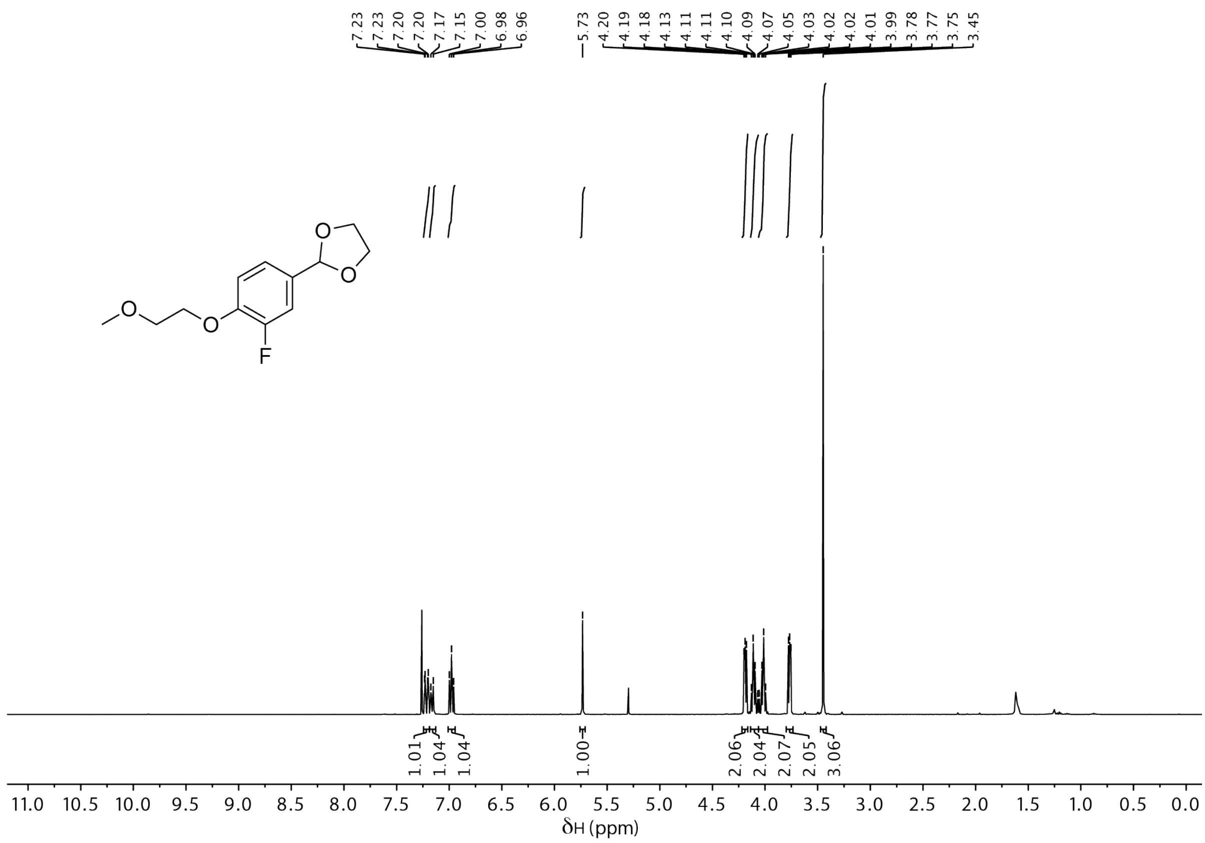

Figure S40. ^{1}H NMR spectrum of compound **7** (500 MHz, $CDCl_3$).

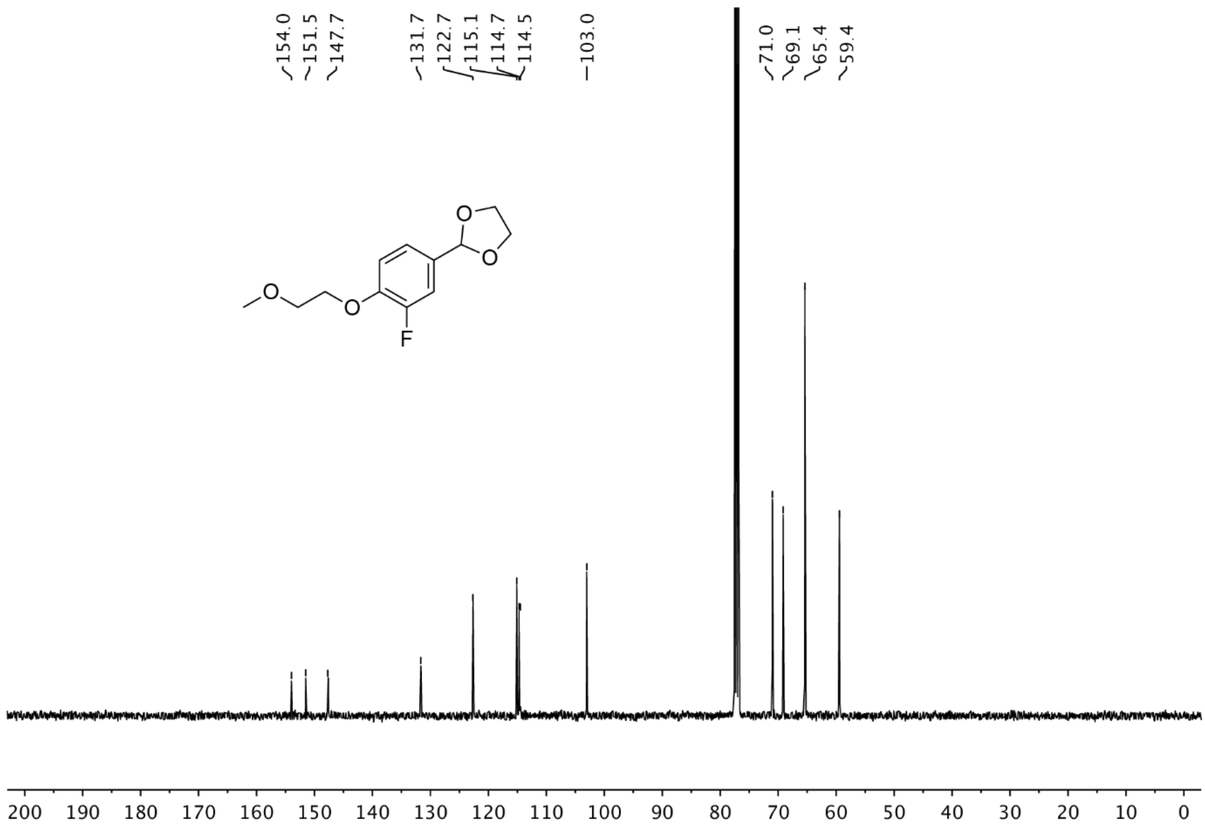

Figure S41. ^{13}C NMR spectrum of compound **7** (126 MHz, $CDCl_3$).

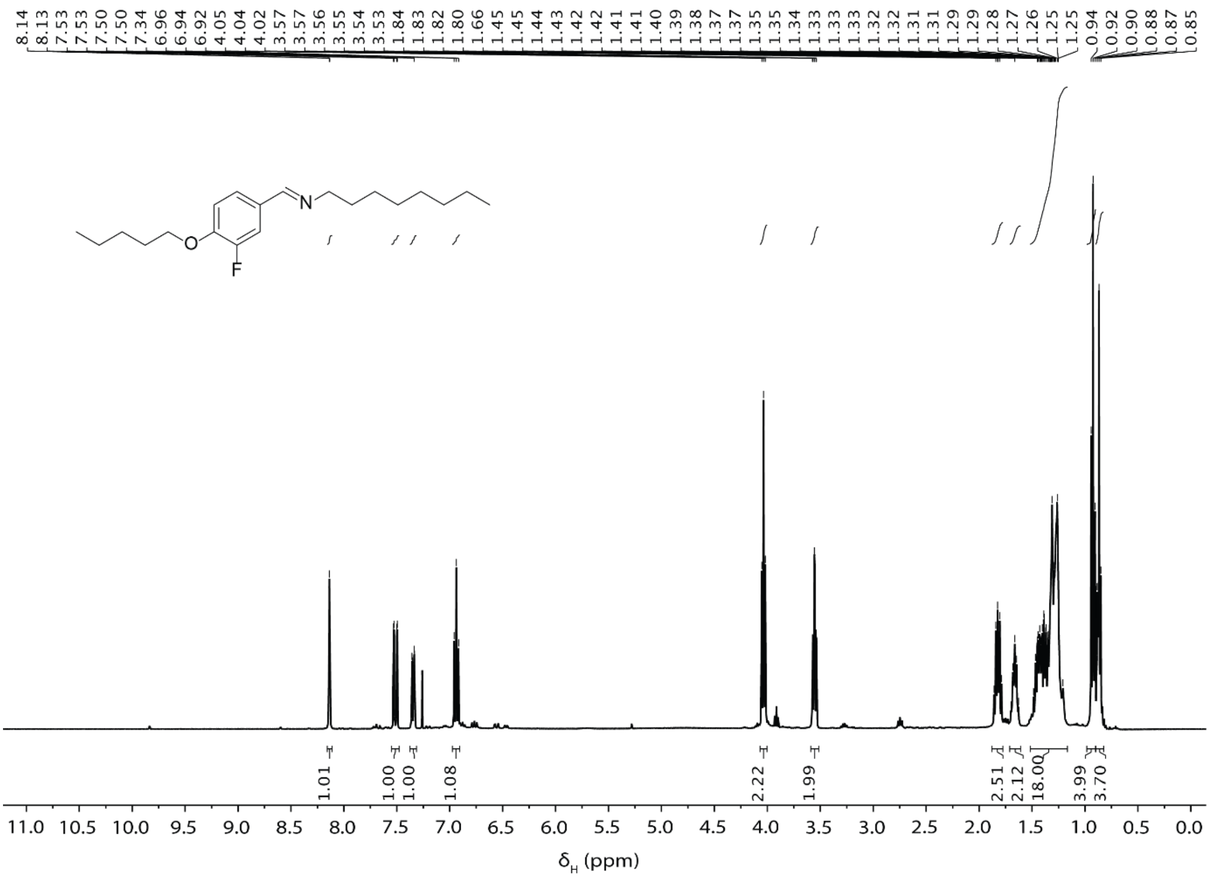

Figure S42. ^{1}H NMR spectrum of compound **S9** (400 MHz, $CDCl_3$).

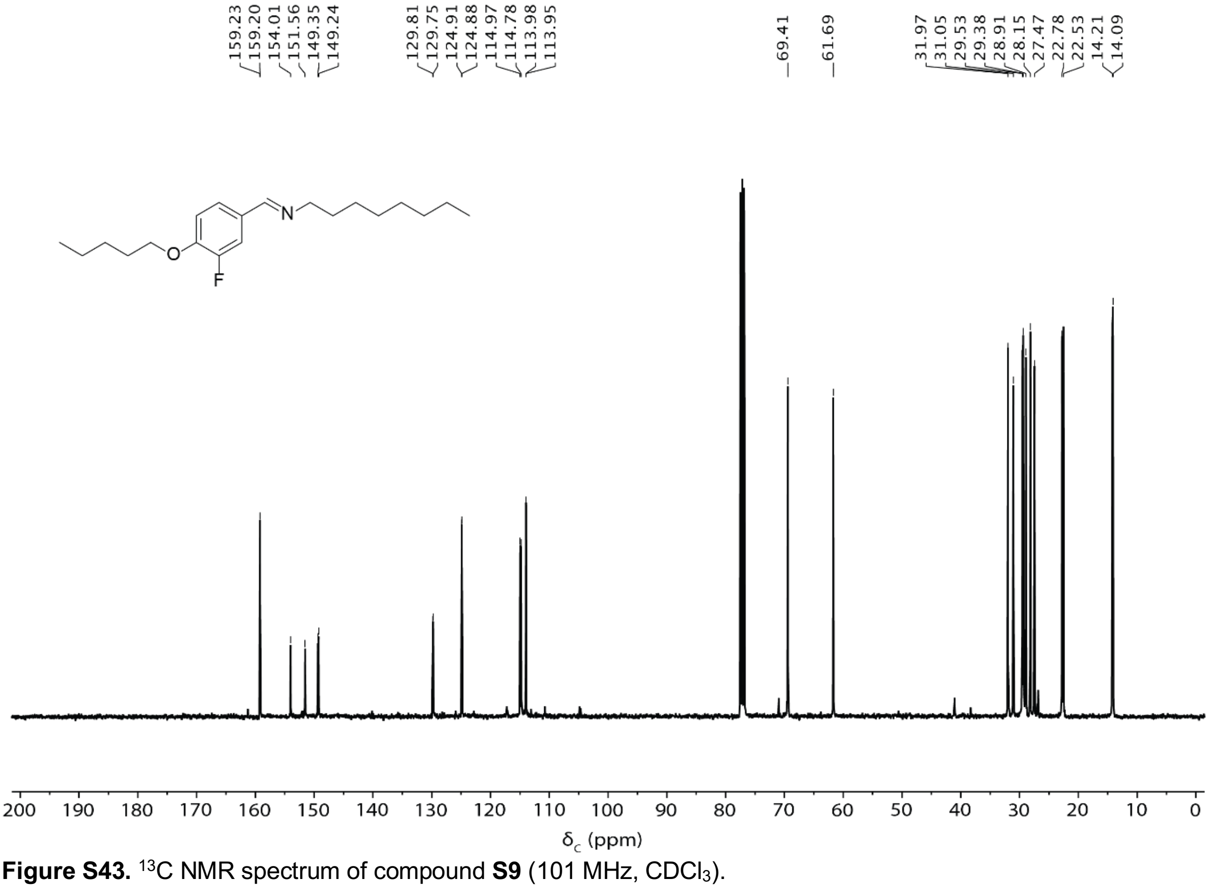

Figure S43. ^{13}C NMR spectrum of compound **S9** (101 MHz, $CDCl_3$).

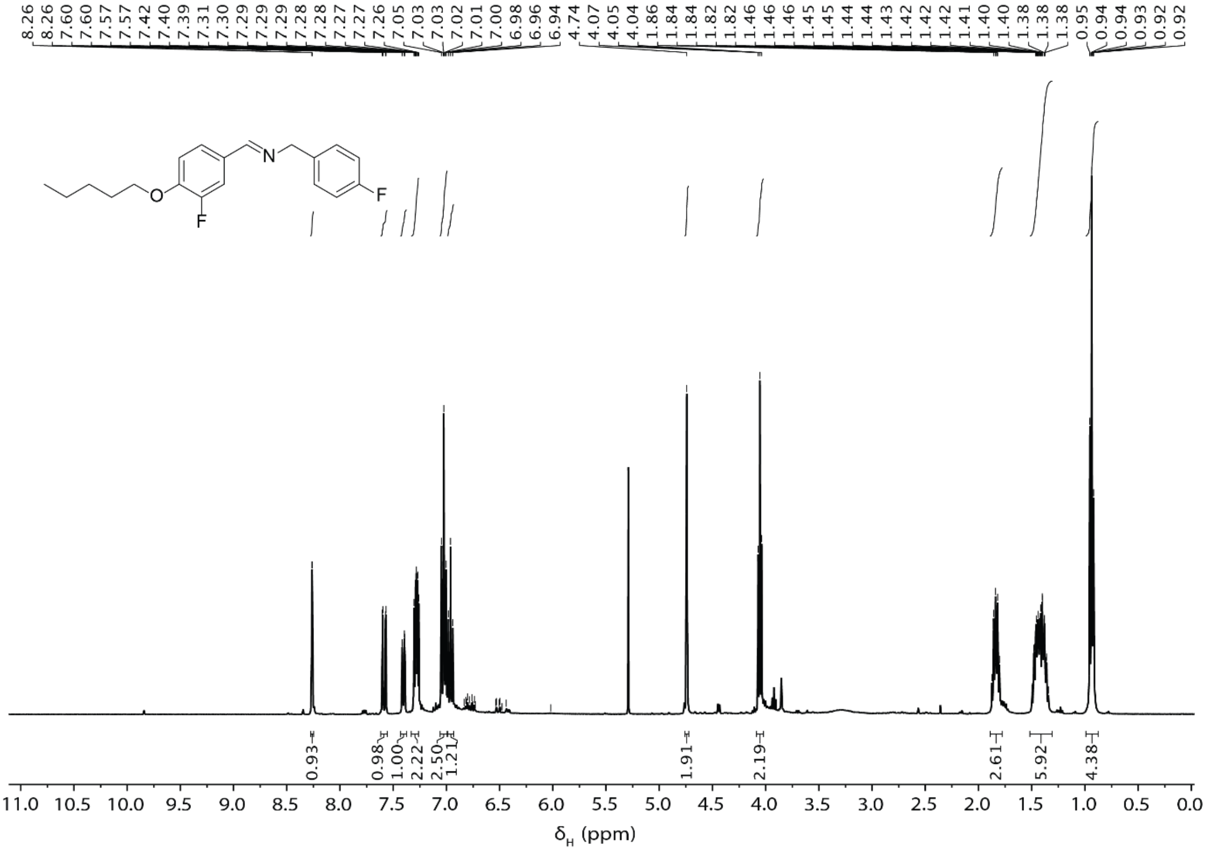

Figure S44. ^{1}H NMR spectrum of compound **S10** (400 MHz, $CDCl_3$).

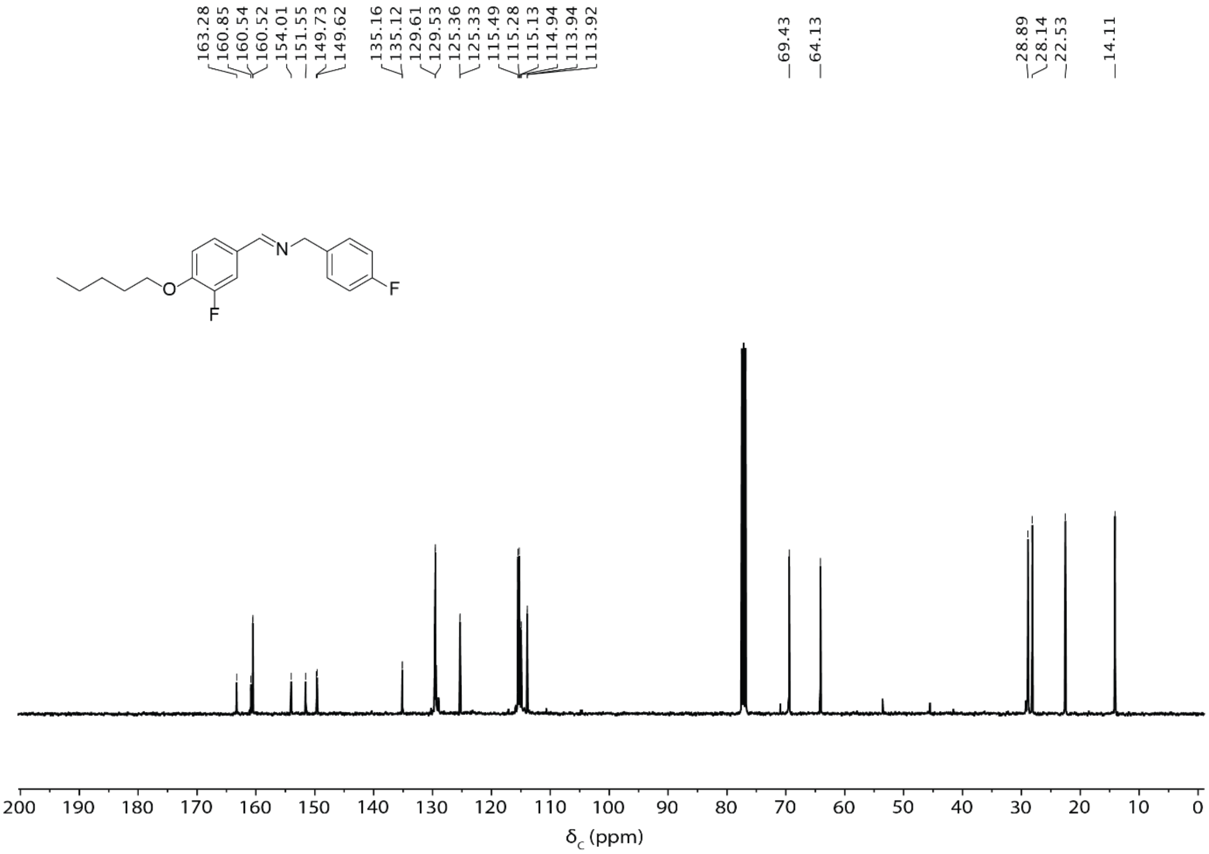

Figure S45. ^{13}C NMR spectrum of compound **S10** (101 MHz, $CDCl_3$).

14. Supplementary references.

[1] F. della Sala, E. R. Kay, *Angew. Chem. Int. Ed.* **2015**, *54*, 4187–4191; *Angew. Chem.* **2015**, *127*, 4261–4265.

[2] F. Rastrelli, S. Jha, F. Mancin, *J. Am. Chem. Soc.* **2009**, *131*, 14222–14224.

[3] M. Kim, Y. Su, A. Fukuoka, E. J. M. Hensen, K. Nakajima, *Angew. Chem. Int. Ed.* **2018**, *57*, 8235–8239; *Angew. Chem.* **2018**, *130*, 8367–8371 .
